# WHO Environmental Noise Guidelines for the European Region: A Systematic Review on Environmental Noise and Effects on Sleep

**DOI:** 10.3390/ijerph15030519

**Published:** 2018-03-14

**Authors:** Mathias Basner, Sarah McGuire

**Affiliations:** Division of Sleep and Chronobiology, Department of Psychiatry, Perelman School of Medicine at the University of Pennsylvania, Philadelphia, PA 19104, USA; smcgu@upenn.edu

**Keywords:** sleep, transportation noise, wind turbine noise, hospital noise

## Abstract

To evaluate the quality of available evidence on the effects of environmental noise exposure on sleep a systematic review was conducted. The databases PSYCINFO, PubMed, Science Direct, Scopus, Web of Science and the TNO Repository were searched for non-laboratory studies on the effects of environmental noise on sleep with measured or predicted noise levels and published in or after the year 2000. The quality of the evidence was assessed using GRADE criteria. Seventy four studies predominately conducted between 2000 and 2015 were included in the review. A meta-analysis of surveys linking road, rail, and aircraft noise exposure to self-reports of sleep disturbance was conducted. The odds ratio for the percent highly sleep disturbed for a 10 dB increase in L_night_ was significant for aircraft (1.94; 95% CI 1.61–2.3), road (2.13; 95% CI 1.82–2.48), and rail (3.06; 95% CI 2.38–3.93) noise when the question referred to noise, but non-significant for aircraft (1.17; 95% CI 0.54–2.53), road (1.09; 95% CI 0.94–1.27), and rail (1.27; 95% CI 0.89–1.81) noise when the question did not refer to noise. A pooled analysis of polysomnographic studies on the acute effects of transportation noise on sleep was also conducted and the unadjusted odds ratio for the probability of awakening for a 10 dBA increase in the indoor L_max_ was significant for aircraft (1.35; 95% CI 1.22–1.50), road (1.36; 95% CI 1.19–1.55), and rail (1.35; 95% CI 1.21–1.52) noise. Due to a limited number of studies and the use of different outcome measures, a narrative review only was conducted for motility, cardiac and blood pressure outcomes, and for children’s sleep. The effect of wind turbine and hospital noise on sleep was also assessed. Based on the available evidence, transportation noise affects objectively measured sleep physiology and subjectively assessed sleep disturbance in adults. For other outcome measures and noise sources the examined evidence was conflicting or only emerging. According to GRADE criteria, the quality of the evidence was moderate for cortical awakenings and self-reported sleep disturbance (for questions that referred to noise) induced by traffic noise, low for motility measures of traffic noise induced sleep disturbance, and very low for all other noise sources and investigated sleep outcomes.

## 1. Introduction

Sleep is a biological imperative and a very active process that serves several vital functions [1]. Undisturbed sleep of sufficient length is essential for daytime alertness and performance, quality of life, and health [2]. Noise has been shown to fragment sleep, reduce sleep continuity, and reduce total sleep time [3,4]. Numerous experimental studies have demonstrated that sleep restriction causes, among others, changes in glucose metabolism and appetite regulation, an attenuated immune response to vaccination, impaired memory consolidation, and dysfunction of blood vessels [5,6,7,8,9,10]. These are precursors for manifest diseases like obesity, diabetes, high blood pressure, and probably also dementia [11,12]. The epidemiologic evidence that chronically disturbed or curtailed sleep is associated with the negative health outcomes mentioned above is overwhelming [1,13]. For these reasons, noise-induced sleep disturbance is considered one of the most important non-auditory effects of environmental noise exposure [14].

Sleep and the effects of noise on sleep can be measured in multiple ways [15]. The gold standard for measuring sleep is polysomnography, which is the simultaneous measurement of (at least) brain electrical potentials (electroencephalogram, EEG), eye movements (electrooculogram, EOG), and muscle tone (electromyogram, EMG). The night is usually divided into 30-s epochs and a sleep stage (or awake) is assigned to each epoch based on typical patterns in the EEG, EOG, and EMG and according to standard criteria [16,17]. Rapid eye movement (or REM) sleep is differentiated from non-REM stages S1 through S4 (or N1 through N3 according to the newer AASM criteria [17]). Stages S3 and S4 (or N3) are also called deep or slow wave sleep (SWS). Continuous bouts of SWS and REM sleep are important for memory consolidation and sleep recuperation, while superficial sleep stage S1 and wake time do not relevantly contribute to sleep recuperation [18]. Polysomnography is currently the only methodology that provides detailed information on sleep stages, sleep structure, and shorter cortical arousals. However, it is somewhat invasive, and trained personnel are needed to attach and detach electrodes and to visually score sleep stages (with known inter-rater variability [19]). This restricts the sample size and generalizability of polysomnographic studies. Simpler methods with similar informative value compared to polysomnography are needed to increase generalizability of noise-effects studies [20].

Other less invasive but typically less sensitive methods include actigraphy and signaled awakenings. Actigraphy infers sleep or wake from wrist movements measured with a watch-like device that is usually worn for 24 h [21]. These devices have been introduced to the consumer market and have become more and more popular over the past years, with potential avenues for future noise-effects research. In studies using signaled awakenings, participants are asked to push a button whenever they wake up during the night, which requires both waking consciousness and the motivation of the subject to push the button, which explains the low sensitivity of this methodology. Finally, questionnaires may be used to ask about awakenings, sleep latency and other aspects of sleep quality. They can refer to the last night or to longer time periods. As humans are unconscious for most of the sleep period, subjective assessments of sleep may not agree with objective measurements, and misattributions are possible (e.g., a subject wakes up spontaneously, regains consciousness, and then perceives a noise event). Also, the subject may use his/her answer to make a political statement if the question explicitly asks about the effect of a noise source. Regardless of the limitations outlined above, self-assessments of sleep disturbance are nevertheless important endpoints for studies on the effects of noise on sleep, and they have been used successfully to describe exposure-response relationships and inform analyses on the burden of environmental noise on disease [14,22]. The different methods for measuring sleep are discussed in greater detail in Basner et al. [15].

**Figure 1:**
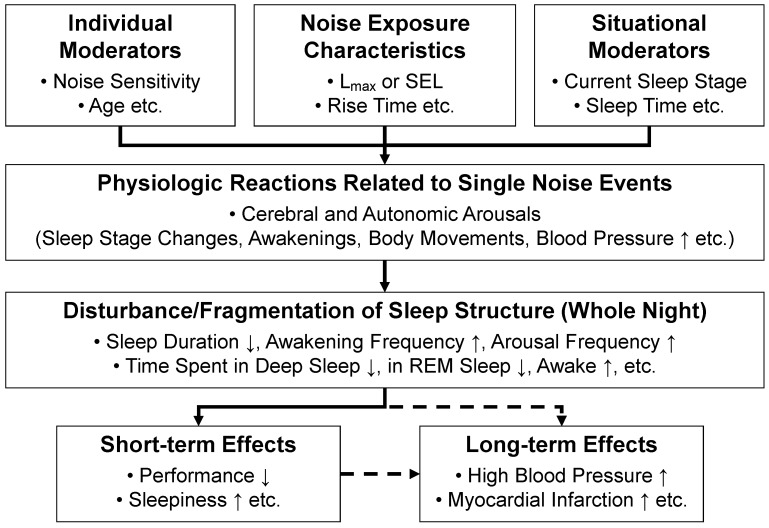
Effects of noise on sleep. It is hypothesized that health consequences will develop if sleep is relevantly disturbed by noise over long time periods (dashed lines; figure reproduced from Basner et al. [25]).

The auditory system has a watchman function and constantly scans the environment for potential threats. Humans perceive, evaluate and react to environmental sounds even while asleep [23]. At the same sound pressure level, meaningful noise events are therefore more likely to cause arousals from sleep than less meaningful events. During the night, noise can often be described as intermittent (i.e., discrete noise events rather than a constant background noise level). In this case, the effects on sleep are primarily determined by the number and acoustical properties (e.g., maximum SPL, spectral composition) of single noise events (Figure 1). Noise may be accompanied by vibrations (e.g., rail noise), and the combination of noise and vibration induces higher degrees of sleep disturbance than noise alone [24]. Whether or not noise will disturb sleep also depends on situational (e.g., depth of sleep phase [25], background noise level [26]) and individual (e.g., noise sensitivity) moderators [23]. Repeated noise-induced arousals impair sleep quality and recuperation through changes in sleep structure including reduced sleep continuity [27], delayed sleep onset and early awakenings, less deep and REM sleep, and more time spent awake and in superficial sleep stages (Figure 1) [25,28]. Noise may also prevent a subject from falling asleep again after a spontaneous or noise-induced awakening. Deep and REM sleep have been shown to be important for sleep recuperation in general and memory consolidation specifically [10].

Non-acoustic factors can also affect sleep: external (e.g., high temperature and humidity) and internal (e.g., sleep disorders, nightmares) factors may induce arousals from sleep, complicating the unequivocal attribution of arousals from sleep to noise [29]. At the same time, classical indicators of fragmented sleep (e.g., awakenings, body movements) are part of the physiological sleep process and occur multiple times throughout the night in healthy sleepers and environments without external stressors, with no pathologic consequences. For example, a healthy adult briefly awakens ca. 20 times during an 8 h bed period (most of these awakenings are too short to be remembered the next morning) [30]. It is currently unclear how many additional noise-induced awakenings are acceptable and without consequences for sleep recuperation and health, especially given the large inter-individual differences in the susceptibility to noise. Although compensatory mechanisms have been observed [28], it is unclear at what point these mechanisms are exhausted or what biological cost they carry. In typical noise scenarios, noise-induced sleep-disturbance is usually less severe than, e.g., that observed in clinical sleep disorders like obstructive sleep apnea [31].

Short-term effects of noise-induced sleep disturbance include impaired mood, subjectively and objectively increased daytime sleepiness, and impaired cognitive performance [32,33]. It is hypothesized that noise-induced sleep disturbance contributes to the increased risk of cardiovascular disease if individuals are exposed to relevant noise levels over months and years (dashed lines in Figure 1). Recent epidemiologic studies indicate that nocturnal noise exposure may be more relevant for the genesis of long-term health outcomes like cardiovascular disease than daytime noise exposure, probably also due to the fact that people more consistently are at home during the night than during the day [34]. Given the many vital biological functions of sleep, and the fact that acutely curtailed or fragmented sleep has immediate consequences for next day alertness and performance, the effects of noise on sleep should not solely be judged based on long-term health consequences. Sleeping satisfies a basic need and is pleasurable if undisturbed and of sufficient length (very much like eating when hungry). Sufficient sleep increases, among others, alertness, mood, productivity, and creativity [2]. Therefore, sleep disturbance (induced by noise or other external or internal factors) needs to be minimized even without clearly established links to long-term health consequences.

One of the main goals of noise effects research is to derive exposure-response functions that can then be used for health impact assessments and ultimately to inform political decision making [3]. Numerous studies have associated several transportation noise sources (e.g., road, rail, and aircraft noise) with awakenings, briefer brain activations, and vegetative arousals (e.g., increases in heart rate and blood pressure) in both laboratory and field settings [25]. Unfortunately, sample sizes and response rates of the studies that are the basis for exposure-response functions were usually low, which restricts generalizability of the latter. These functions are usually sigmoidal (s-shaped) and show monotonically increasing reaction probabilities with increasing maximum sound pressure levels (SPL) or sound exposure levels (SEL). Maximum SPLs as low as 33 dBA induce physiological reactions during sleep, i.e., once the organism is able to differentiate a noise event from the background, physiologic reactions can be expected (albeit with a low probability at low noise levels) [35]. This reaction threshold should not be confused with limit values used in legislative and policy settings, which are usually considerably higher. As exposure-response functions are typically without a clearly discernible sudden increase in sleep disturbance at a specific noise level and because of individual variation in noise sensitivity, defining limit values is not a straightforward task. It usually involves expert judgement of the existing evidence (e.g., Night Noise Guidelines [36]), and political weighing of negative health consequences of noise and societal benefits of the noise source.

Equivalent noise levels are often used in surveys and epidemiologic studies as long-term average exposure metrics, and are therefore also often found in legislative and policy contexts. For example, the Night Noise Guidelines for Europe of the World Health Organization (WHO) define effects of nocturnal noise based on annual average outdoor L_night_ ranges [36]. The value of equivalent noise levels in describing the effects of noise on sleep is more limited, as different noise scenarios may calculate to the same equivalent noise level, but differ substantially in their sleep disturbing properties [25]. There is general agreement that the number and acoustical properties of single noise events better reflect the actual degree of nocturnal sleep disturbance in a single night [35]. It is thus questionable whether L_night_ can be used as the only indicator for predicting the effects of noise on sleep and the consequences of noise-induced sleep disturbance, or whether supplemental noise indicators are needed [25].

Subjects exposed to noise usually habituate. For example, the probability that noise causes physiologic reactions is in general higher during the first nights of a laboratory experiment compared to the last nights [28], and exposure-response relationships derived in the field (where subjects have often been exposed to the noise for many years) are usually much shallower than those derived in laboratory settings, which often include exposure to unfamiliar noise events in an unfamiliar environment [35,37]. Habituation is a reasonable mechanism that preserves energy resources. However, habituation is not complete, i.e., subjects continue to react to noise events even after several years of noise exposure. Unfortunately, little is known about individual differences in the ability to habituate to noise and potential predictors. Importantly, activations of the vegetative nervous system habituate to a much lesser degree to noise compared to cortical arousals. They provide biologic plausibility for the observed association between long-term noise exposure and cardiovascular disease [28,38,39]. It is also possible that exposed subjects become more sensitive to the effects of noise on sleep. This sensitization may be related to, e.g., individual changes (like aging, new incident disease), changes in noise exposure, or changes in media coverage. However, scientific knowledge about noise sensitization is currently very limited.

Sensitivity to nocturnal noise exposure varies considerably between individuals. Little is known about characteristics that predict someone’s sensitivity to nocturnal noise-exposure. Men were found to be more sensitive to traffic noise than women [28], and specific features in the electric potentials generated by the brain (so-called sleep spindles) were associated with resiliance to noise-induced sleep disturbance [23]. The elderly, children, shift-workers, and patients with pre-existing (sleep) disorders are considered risk groups for noise-induced sleep disturbance [4]. Hospitals are often required to have additional sound insulation to reflect the increased sensitivity of the patient population.

In conclusion, undisturbed sleep is a prerequisite for high daytime performance, well-being and health. Environmental noise can disturb sleep and impair sleep recuperation. Reliable and up-to-date exposure-response relationships between environmental noise exposure and sleep disturbance are needed to inform political decision making and to help mitigate the effects of environmental noise on sleep. To provide updated recommendations since the last guidelines, we performed a systematic review of the literature on the effects of noise on sleep published in or after the year 2000. We performed a meta-analysis of surveys linking environmental noise exposure to self-reports of troubles falling asleep, awakening during the night, and sleep disturbance, and derived exposure-response relationships. We also performed a pooled analysis of studies on the acute effects of road, rail, and aircraft noise on sleep, and derived exposure-response functions between the maximum sound pressure level of individual noise events and the probability to wake up.

## 2. Methods

### 2.1. Mapping of Identified Reviews

A search for reviews on the effects of environmental noise on sleep was completed by WHO during spring 2014. The purpose was to determine if there were existing systematic reviews that could be used to provide evidence on noise and sleep outcome measures. In the literature search, sixteen reviews were identified. The quality of reviews was evaluated using the AMSTAR criteria [40]. Nine of the reviews were excluded as they did not have an a priori design, did not include a comprehensive literature review, or were on a topic irrelevant for this evidence review [41,42,43,44,45,46,47,48,49]. Of the seven remaining reviews, two examined the effects of noise on sleep in specific geographic regions only [50,51], one review only included studies in which there was a change in noise level [52] (a topic covered within the intervention evidence review), and 1 review only included studies that examined the relationship between sleep outcomes and noise sensitivity not the association with noise level [53]. The three remaining reviews were broader in content and examined the effects of aircraft [54], ambient [55], and wind turbine noise on sleep [56]. Data from individual studies were not pooled in any of the reviews; results from individual studies were presented qualitatively only. Therefore, it was determined that for all sleep outcome measures an updated search and review of individual studies would need to be conducted.

### 2.2. Search for Individual Studies

A search for individual studies was conducted by WHO which resulted in a total of 1159 hits. This search was not restricted by the year of publication. The titles and abstracts of these papers were reviewed by two independent reviewers and 51 were determined to be on relevant topics. The search terms included the study design (prospective, retrospective, cohort, longitudinal, cross-sectional, case control, ecological), type of noise source (environmental, community, traffic, railway, wind, aircraft, leisure, hospital) and outcome measure (insomnia, sleep, cortical awakening and arousal, autonomic arousal). After conducting this initial search, it was determined that several key papers in the field were not identified. Therefore, a second literature search was conducted using the same terms as provided by WHO, except terms that referred to study design were removed as they are not always applicable to studies on the effects of noise on sleep (the exact search term can be found in Section S6 of the supplement). The second literature search resulted in 10,029 hits and 216 additional papers were identified after reviewing titles and abstracts. The databases searched included PSYCINFO, PubMed, Science Direct, Scopus, Web of Science and the TNO Repository. A total of 69 additional papers which were mentioned in the identified literature reviews and in the meta-analysis by Miedema and Vos (2007) [22] were also included. Therefore, the literature search resulted in a total of 336 identified papers. The search also included gray literature, ICBEN and INCE conference proceedings were searched. The two literature searches were conducted in 2014. Additional searches were conducted on 30 July 2015 and 1 December 2015 to identify any additional studies while finalizing this review, two additional reports on transportation noise, one on hospital noise, and three on wind turbine noise were included based on these final searches.

### 2.3. Inclusion and Exclusion Criteria

**Figure 2:**
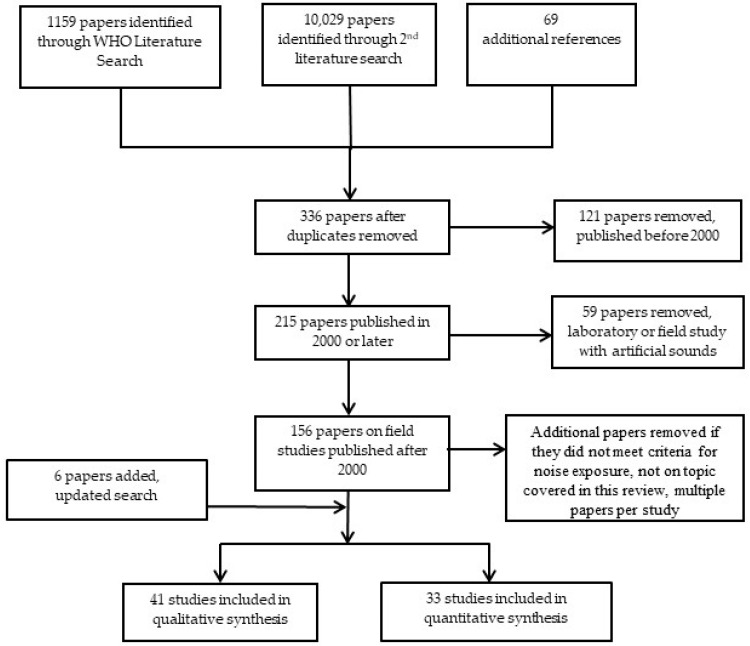
Flow of study selection.

Not all of the individual studies identified in the literature search were included in this evidence review. For all noise sources, studies conducted in the laboratory or those studies in which sounds were played back artificially were excluded due to low ecological validity. Studies conducted in the laboratory or studies that play back artificial sounds have typically found a higher probability of awakening to noise events than field studies [37,57,58]. Intervention studies (except for hospital noise) were excluded as they were covered in the intervention evidence reviews. Also, studies on sleep medication use were not covered in this review, as they initially were supposed to be covered in the mental health evidence review. However, the latter does not specifically cover sleep medication use. This is a limitation of this review, as sleep medication use can be an important indicator for noise-induced sleep disturbance. Sleep medication use is covered in the Night Noise Guidelines for Europe [36], and the reader is referred to those for a relatively recent review. In addition, for road, rail, and aircraft noise, only those studies published in the year 2000 or later were included, as this review is meant to be an update since the last guidelines. All studies on hospital noise were included though as this topic was not covered in detail in the previous guidelines. To be included in the review, studies must also have included measured or predicted noise levels for the participant’s home; those that only included subjective evaluations of the noise or distance to the noise source were excluded. Studies that included noise levels not specific to the participant’s home address were also excluded. Also studies had to have at least 2 different noise level categories examined in the study. In total 74 studies contributed to this review. Studies that did not meet the inclusion criteria and were excluded from the qualitative and quantitative analysis are listed in section S7 of the Supplement. A flow diagram of the selection and elimination of studies is shown in Figure 2.

### 2.4. Risk of Bias and Quality Assessment

Socio-economic status, age and gender were considered important confounders (i.e., variables associated both with the exposure and the outcome), but the use of these variables for adjustment was variable, so we did not exclude studies based on whether or not they adjusted for confounding by these variables.

The risk of bias in the studies reviewed is primarily a consequence of (a) the methodology used to measure sleep and noise-induced sleep disturbance and (b) the willingness of subjects to participate in a study on the effects of noise on sleep. Unfortunately, (a) and (b) are inversely related in such a way that less biased measurement techniques are associated with a higher selection bias and vice versa.

Information bias: Those studies that used polysomnography, or made continuous heart rate and blood pressure measurements during the sleep period were considered to have the lowest risk of information bias. Polysomnography, the simultaneous measurement of the electroencephalogram (EEG—brain activity), electrooculogram (EOG—eye movement), and electromyogram (EMG—skeletal muscle tone), is considered the gold standard for measuring sleep, and evaluating sleep fragmentation and sleep structure. However, electrodes cause some discomfort, may influence sleep (especially during the first measurement night), and thus introduce bias. Heart rate and blood pressure during the night will increase when an individual has brief autonomic or cortical arousals and therefore these measurements also provide a sensitive measure of sleep fragmentation [20].

The risk of information bias for studies that measured motility was considered moderate. Motility is measured typically using wrist worn devices (i.e., actigraphs). While awakenings or arousals during the night often occur together, individuals can be awake without moving which results in misclassification. Comparison studies between awakenings identified using actigraphy and polysomnography have found high sensitivity in identifying sleep epochs during the night but a low specificity (below 0.40) in identifying wake epochs [59]. 

Studies in which self-reported measures of sleep were used were considered to have a high risk of information bias. Subjects are not aware of themselves and their surroundings for most of the night, and relevant physiologic reactions are often not consciously perceived and remembered in the morning. Also, misattributions are possible (e.g., a subject wakes up spontaneously, regains consciousness, and then perceives a noise event). When studies specifically ask about how a particular noise source affects sleep, an individual’s response may (at least partially) reflect his or her attitude or feelings toward nighttime noise rather than disrupted sleep itself.

Information bias could occur not only due to sleep measurement methods, but also could arise from the methods used to quantify environmental noise. Due to variability in traffic across days, noise measurements should be made over a sufficient time period (minimally 1 week). For noise predictions, at a minimum data that is representative of the current traffic patterns should be used in the calculations for a study to have low risk of bias.

Selection bias: While studies using polysomnography for the measurement of sleep may have low information bias, they suffer from high selection bias. These studies often only include healthy individuals without sleep disorders. Due to the high methodological expense, sample sizes are typically low. Therefore the results may not be representative of the effects of noise on sleep in the general population. In addition, response rates for taking part in these studies are low as the instrumentation for measuring sleep requires trained personal to go to participant’s home each night and morning to apply and remove the electrodes, and the equipment that needs to be worn induces discomfort. Compared to studies using PSG, studies relying on self-reported sleep, in which participants are asked to fill out a questionnaire or complete an interview, have in general lower selection bias, higher response rates, and larger sample sizes. The results may therefore be more representative of the general population. However, this methodology also suffers from the highest information bias.

Publication bias: Publication bias refers to the fact that studies with positive findings are more often both submitted to and accepted by scientific journals. This likely biases the published studies to positive findings. It is, however, difficult to assess the consequences of publication bias.

## 3. Polysomnography Measured Cortical Awakenings for Road, Rail, and Aircraft Noise

As described in detail in Section 1, polysomnography is considered the gold standard for measuring sleep, its structure, and related events. Sleep structure varies systematically over the course of the night, with deep sleep (stages 3 or 4, or N3 according to the new classification) dominating the first half of the night and REM sleep and superficial sleep stages 1 and 2 (or N1 and N2) dominating the second half of the night. Field studies (including the four studies discussed in detail below [35,60,61,62]) typically allow subjects to adhere to their normal bed times. Sleep duration thus varies systematically both between subjects but also within subjects (if a subject is measured for multiple nights). This within- and between-subject variability in sleep duration complicates the assessment of the effects of noise on sleep duration and whole night sleep parameters, and introduces substantial non-noise variance to the data. Even sleep architecture (i.e., the distribution of sleep stages) will be affected by fluctuations in sleep period duration (regardless of whether sleep stages are expressed in minutes or % of sleep period time), as sleep stages are not evenly distributed over the course of the night. For these reasons, we concentrated our analysis on the effects of traffic noise on sleep on the reaction of the sleeper to single noise events. Spontaneous and noise-induced awakenings also increase with increasing sleep period time, but it is relatively easy to account for the latter in single event analyses. Furthermore, relationships between whole-night noise exposure descriptors (i.e., L_night_) and single event metric outcomes (i.e., awakenings) have been previously described, and the reader is referred to these [25].

Four studies were identified on study selection for which the effects of road, rail, or aircraft noise on polysomnographically measured sleep was evaluated. Two studies identified in the literature review but not included in the re-analysis include one road traffic and rail noise study and one aircraft noise study. Aasvang et al. [61] conducted a field study examining the effect of railway and road traffic noise on sleep in Oslo, Norway. Twenty of the subjects were exposed to railway noise and twenty to road traffic noise. The subjects participated for two consecutive nights. Several sleep variables were examined in relation to the maximum noise level inside the bedroom for the entire night due to road traffic or rail noise. Wake after sleep onset (WASO) was found to increase with the maximum noise level of train noise with a 30 min increase in WASO found for those subjects exposed to noise levels above 50 dBA compared to those exposed to levels less than 50 dBA. Also a decrease in REM sleep with noise level was found with rail noise, however no significant changes in any sleep parameter was found for road traffic noise. The data from the study by Aasvang et al. [61] was not included in the re-analysis because single transportation noise events and associated awakenings had not been scored. Flindell et al. [62] conducted a study on the effects of aircraft noise around Manchester airport. Eighteen subjects took part for 5 consecutive nights. All subjects were between the ages of 30 to 40 years old. Noise levels were recorded within the bedroom. There was no significant change in sleep between a high noise and a low noise area, but the indoor noise exposure in both areas was similar. The study found increases in the number of awakenings, total durations of stage 1 sleep, number of REM sleep periods and changes in the frequency content of the EEG associated with higher numbers of ANEs occurring during the sleep period. The data from the Flindell et al. [62] study was not available for inclusion in the re-analysis.

Single event based analysis was completed in two studies conducted by the German Aerospace Center (DLR), both of which used similar methodology and were included in the re-analysis. The STRAIN study was conducted to investigate the effect of aircraft noise on sleep [35]. The study was conducted between September 2001 and November 2002 and included 64 residents between the ages of 18 to 61 years (average age 38 years, 55% female) who lived around Cologne-Bonn Airport. The DEUFRAKO study was conducted to investigate the effect of rail noise on polysomnographically measured sleep [60]. The study was conducted between February 2008 and July 2009 and included 33 individuals between the ages of 22 and 68 years (average age 36 years, 67% female) who lived near Cologne and Bonn close to railway lines. In both studies, subjects participated for nine consecutive nights and indoor noise levels were recorded in the bedroom. Physiological reactions to road traffic noise were also measured. The raw data for these two datasets were obtained from DLR and used to derive exposure-response relationships for the probability of a sleep stage change to wake or S1; the STRAIN dataset was used for aircraft noise, the DEUFRAKO dataset was used for train noise, and the STRAIN and DEUFRAKO data were combined for road traffic noise.

### 3.1. Event-Related Analysis

For both studies, sleep stages were scored according to the standard criteria of Rechtschaffen and Kales using 30-s epochs [16]. Epochs scored as Movement Time were re-classified as wake. Individuals who visually scored the polysomnography data were blinded to the occurrence of noise events. For the STRAIN study, data from 61 of the 64 participants contributed to the analysis, two were excluded due to constant snoring and one was excluded due to an intrinsic sleep disorder.

Road, rail, and aircraft events were identified by listening to indoor sound recordings and the start and end of each noise event was scored. For each noise event, the first sleep stage affected by a noise event (first noise epoch) was defined as the first epoch that contained more than 15 s of the event [35]. If the subject was asleep in the epoch prior to the first noise epoch (Stages 2, 3, 4, or REM sleep) then the next three epochs (90 s) were screened for a transition to wake or Stage S1.

During a road, rail, or aircraft event, additional outdoor or indoor noises can occur. In this analysis a noise event was considered ‘undisturbed’ if the following criteria were met: (1) only events from the same noise source could occur one minute before (e.g., the end of a prior noise event) and 1.5 min after the start of the event and (2) sounds made by the subject such as turning over in bed were allowed before and during the noise event of interest as they could be reactions to the noise. Events defined as ‘disturbed’ consisted of those in which any other noise event occurred 60 s prior or up to 1.5 min after the start of the first noise epoch.

### 3.2. Statistical Analysis

For the analysis, each noise event was annotated with its maximum sound pressure level (L_AS,max_), the age and gender of the exposed subject, the day of the week (weekday/weekend), and time from sleep onset. The primary outcome is binary and reflects an awakening or sleep stage change to stage 1 (1) or no such change in sleep structure (0). Random subject effect logistic regression models with the maximum indoor noise level (L_AS,max_) as the only predictor were performed with the NLMIXED procedure in SAS (version 9.3, SAS institute, Cary, NC, USA), based on the event-related data. The non-liner models were calculated to reflect the clustered nature of the data (i.e., that each subject was exposed to multiple noise events). Both unadjusted models and models adjusted for age, gender, weekday, and time from sleep onset were calculated. Point estimates and 95% confidence intervals were generated with estimate statements in Proc NLMIXED. Unadjusted models were used to derive the exposure-response relationships. While additional factors such as prior sleep stage, time of night, duration of the event, age, gender have been found to be important effect moderators [35,60], assumptions have to be made for the values of these parameters when deriving exposure-response relationships between noise level and probability of awakening. Therefore, only the noise level of the event was included when deriving these models.

**Figure 3:**
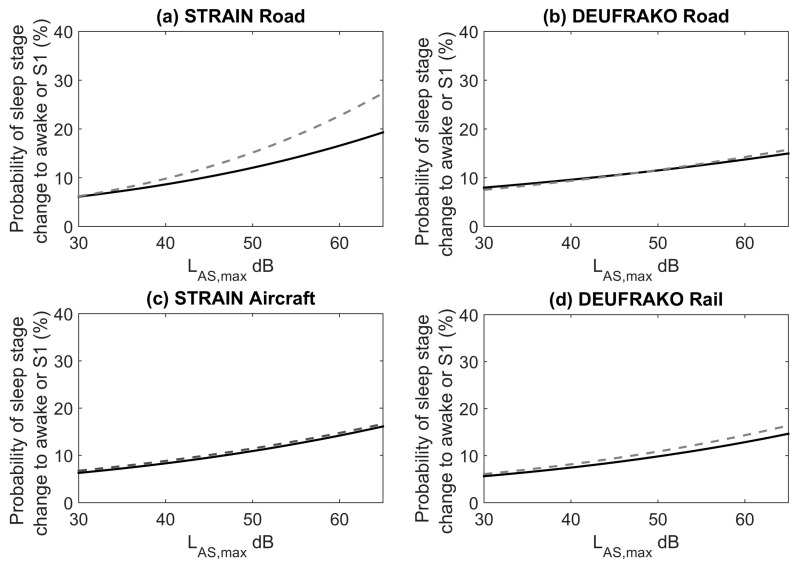
Probability of a sleep stage change to awake or S1 in a 90 second time window following noise event onset depending on the maximum indoor sound pressure level (L_AS,max_) for (**a**) STRAIN road traffic (*N* = 61 subjects); (**b**) DEUFRAKO road traffic (*N* = 33); (**c**) STRAIN aircraft (*N* = 61); and (**d**) DEUFRAKO rail noise events (*N* = 33). Undisturbed events only (black), all events including disturbed and undisturbed events (gray dotted line).

430 subject nights of data from the STRAIN study and 277 subject nights from the DEUFRAKO study contributed to the analysis. Exposure-response relationships for all transportation modes, for only undisturbed events and both disturbed and undisturbed events were calculated and the results are shown in Figure 3. This analysis was completed to examine the potential bias in the exposure-response curves when including or excluding specific noise events. The exposure-response functions are for the probability of a transition to wake and Stage 1 because in the DEUFRAKO study more Stage 1 sleep was scored than in the STRAIN dataset (23.3 min versus 16.6 min), which may be due to inter-rater variability in scoring.

When all events were included in the analysis there was a higher probability of transitions to wake and S1 for road traffic noise in the STRAIN study compared to the probability for transitions for undisturbed noise events. This may be due to simultaneous aircraft noise events that increase awakening probability. However, this was not found for the DEUFRAKO study. For the other noise sources there were only small non-significant changes in the exposure-response relationships when including disturbed noise events. Due to the difference for road traffic noise, however, for the remaining analysis only the undisturbed events were used. The number of noise events contributing to the analysis was 10,546 aircraft events, 7631 train events (including both passenger and freight trains), and 7101 road traffic events in the STRAIN study and 4407 events in the DEUFRAKO study. The road traffic events consisted primarily of single car or truck passings, 843 events consisted of multiple vehicles.

**Figure 4:**
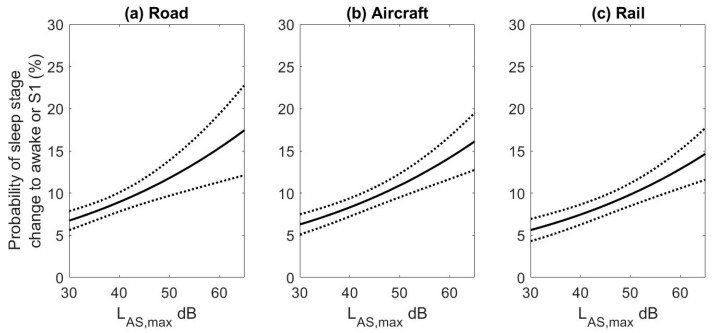
Probability of a sleep stage change to awake or S1 in a 90 s time window following noise event onset depending on the maximum indoor sound pressure level (L_AS,max_) for (**a**) road (STRAIN and DEUFRAKO, *N* = 94 subjects); (**b**) aircraft (STRAIN, *N* = 61); and (**c**) rail noise (DEUFRAKO, *N* = 33). 95% confidence intervals (dashed lines). Results are for the unadjusted model.

The three exposure-response curves for the undisturbed events are shown in Figure 4 for the slow weighted maximum noise level. The road noise data from the STRAIN and DEUFRAKO study were combined as estimates did not differ significantly between studies (OR per 10 dBA 1.45; 95% CI 1.22–1.73 for STRAIN and OR per 10 dBA 1.22; 95% CI 0.98–1.51 for DEUFRAKO, *p* = 0.09). The data for both passenger and freight trains in the DEUFRAKO study were combined as well (OR per 10 dBA 1.40; 95% CI 1.22–1.61 for freight trains and OR per 10 dBA 1.21; 95% CI 0.98–1.50 for passenger trains, *p* = 0.31). While the slow A-weighting is typically used for aircraft noise metrics, the fast weighting is often used for road and rail noise due to the faster temporal profile of the sounds. While not available for the STRAIN study, L_AF,max_ levels were available for the DEUFRAKO study. The mean absolute difference between L_AF,max_ and L_AS,max_ levels was 0.86 dB (2.5–97.5% Range: 0–3.5 dBA) for road traffic, and 0.72 dB (2.5–97.5% Range: 0.0–4.0 dBA) for rail traffic. Overall the average difference in levels was less than 1 dBA and therefore all results are presented using L_AS,max_ levels.

**Figure 5:**
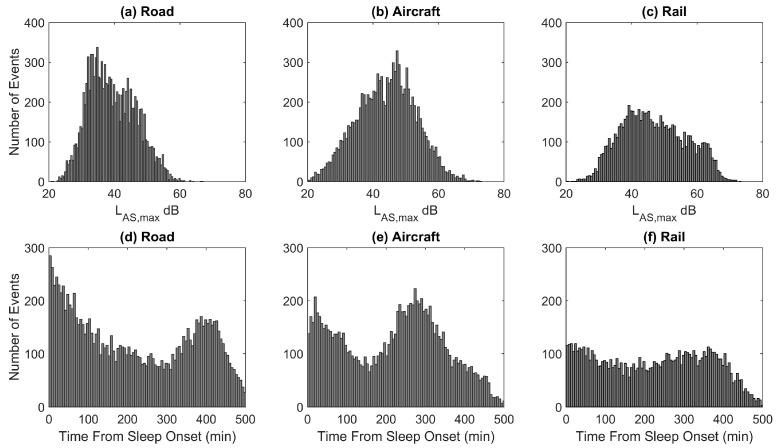
Distribution of indoor noise levels and the time of events relative to sleep onset for (**a**,**d**) road; (**b**,**e**) aircraft; and (**c**,**f**) rail events (all undisturbed noise events from the STRAIN and DEUFRAKO studies used for analysis).

The distribution of indoor noise levels and the timing of events relative to sleep onset for each noise source are shown in Figure 5. The unadjusted odds ratio for sleep stage transitions to wake or Stage 1 for a 10 dBA increase in the slow weighted indoor maximum noise level (L_AS,max_) for all three transportation modes was calculated and the results are shown in Table 1. All odds ratios were statistically significant and differed only marginally between traffic modes. Odds ratios adjusted for age and gender, and odds ratios adjusted for age, gender, day of the week (weekend or weekday), and time from sleep onset were also calculated. Adjusting only marginally reduced the odds ratios, and all estimates were still significantly different from 1. Data for additional confounding variables were not available.

Individuals will not only awaken during the night due to noise events but also spontaneously. It is because of these spontaneous reactions that in Figure 4, even for low noise levels the probability of sleep stage transitions to wake or S1 is greater than 5.0%. The probability of spontaneously awakening during the night was calculated separately for all three transportation sources using virtual events [57]. As each subject was investigated for several nights, the other study nights could be used to determine spontaneous awakening probability. For example, if a noise event occurred in study night #2 two hours after sleep onset, study nights #3–#9 were screened for spontaneous awakenings at the same time from sleep onset as the noise event if this time interval was determined to be free from transportation noise (night #1 was always discarded from the analysis due to a possible first-night effect [63]). The spontaneous awakening rates that were calculated were 6.1% for rail, 7.7% for aircraft, and 8.2% for road noise (all for 90-s intervals relative to onset of the virtual noise event). Three different rates of spontaneous awakening probability were calculated as the value is dependent on the time noise events occurred (as shown in Figure 5d–f the distribution of events during the night varied by noise source). In addition, different spontaneous rates were calculated because for each transportation mode the results are based on data from different subjects.

**Figure 6:**
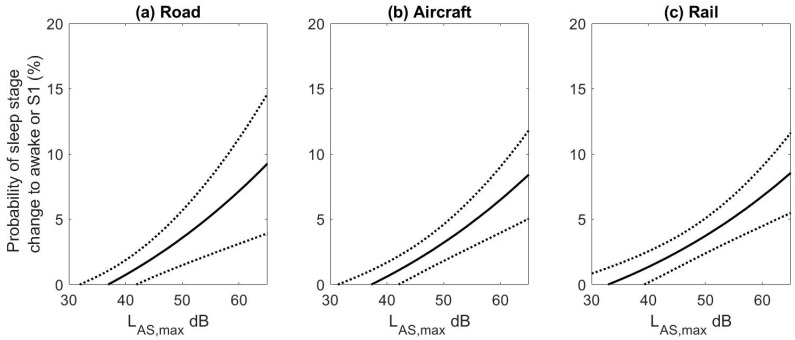
Probability of additional sleep stage changes to awake or S1 in a 90 s time window following noise event onset depending on the maximum indoor sound pressure level (L_AS,max_) for (**a**) road (STRAIN and DEUFRAKO, *N* = 94 subjects); (**b**) aircraft (STRAIN, *N* = 61); and (**c**) rail noise (DEUFRAKO, *N* = 33). 95% confidence intervals (dashed lines). Results are for the three unadjusted models.

The spontaneous awakening probabilities were subtracted from the exposure-response curves, by including the value in the logistic regression equation when deriving the point estimates, to obtain the probability of having an additional awakening attributable to the noise event. Second order polynomials were fit to obtain exposure-response relationships. The exposure-response relationships obtained are shown in Figure 6.

The equations for the probability of additional awakenings due to road, rail, and aircraft noise are:

(1) $$\mathrm{Road}:\;\mathrm{Prob}.\;\mathrm{of}\;\mathrm{Wake}\;\mathrm{or}\;S1=-3.3188-0.0478*L_{\mathrm{AS},\max}+\;0.0037*{\left(L_{\mathrm{AS},\max}\right)}^{2}$$

(2) $$\mathrm{Aircraft}:\;\mathrm{Prob}.\;\mathrm{of}\;\mathrm{Wake}\;\mathrm{or}\;S1=-3.0918-0.0449*L_{\mathrm{AS},\max}+\;0.0034\;*{\left(L_{\mathrm{AS},\max}\right)}^{2}$$

(3) $$\mathrm{Rail}:\;\mathrm{Prob}.\;\mathrm{of}\;\mathrm{Wake}\;\mathrm{or}\;S1=-1.7768-0.0529*L_{\mathrm{AS},\max}+\;0.0033*{\left(L_{\mathrm{AS},\max}\right)}^{2}$$

### 3.3. Conclusions

In the re-analysis conducted, for all transportation modes a significant positive association was found between indoor maximum noise levels of single events and the probability of sleep stage transitions to wake or Stage 1. The noise levels at which the probability of an additional awakening was nonzero varied between transportation modes but was between 33–38 dBA, which is consistent with previous findings [35,64]. While for road traffic noise the odds ratio for awakenings was greater in the STRAIN study than in the DEUFRAKO study, no significant differences were found between the three transportation modes. This finding is in contradiction to the results of a laboratory study conducted by Basner et al. [28] in which road and rail traffic noise resulted in a greater probability of awakening than aircraft noise for events of the same noise level. Also these results are in contradiction to those of Aasvang et al. [61] who found that train noise had a greater effect on sleep than road traffic noise. However, the DEUFRAKO and STRAIN studies were not designed to specifically examine the effect of road traffic noise on sleep. A difference was also not found in awakening probability between train and aircraft noise. However, this comparison was conducted across studies. While polysomnography is a sensitive and objective measure of sleep, sleep stage scoring is performed visually and there can be both high intra- and inter-rater variability in the scoring [65]. Therefore, further studies are still needed in order to determine whether in the field setting the three types of transportation modes have a different effect on awakening probability.

In terms of the applicability of these results to the general population, all four of the studies identified in the review suffer from selection bias. Subjects in these studies were physically healthy and free of intrinsic sleep disorders. The effect of transportation noise on sleep in those with preexisting medical conditions is unknown; the results presented may underestimate the effect of noise on sleep in the general population. We were able to adjust odds ratios for the confounders age and gender, time from sleep onset, and day of the week but did not have access to a more comprehensive set of confounders. The exposure-response functions are based on unadjusted models that contained the maximum sound pressure level as the only predictor. Although the number of noise events that contributed to the exposure-response relationships was large, the latter are nevertheless based on data from a total of *N* = 94 subjects only and these subjects lived in geographically circumscribed regions in Germany. Thus, although the best data set currently available, it is unclear how the exposure-response relationships translate to other populations and regions. More studies with a higher degree in diversity of populations and regions are needed to inform future exposure-response functions.

Finally, it is unclear how the results from single event analyses translate to changes in sleep structure across the whole night, as time in bed is rarely fixed in field studies on the effects of noise on sleep and sleep stages are not evenly distributed across the night (see Section 3 for a discussion). Some research has shown that the body engages in compensatory mechanisms to keep the level of sleep fragmentation low [28]. However, noise-induced awakenings may come at a greater biological cost for recuperation than spontaneous awakenings that are part of the physiologic sleep process [29]. The two studies that did provide whole night sleep estimates also allowed variable individual bed times [61,62]. The limited evidence derived from these two studies does, however, support the notion that nocturnal traffic noise exposure contributes to sleep disturbance on the whole night level.

## 4. Self-Reported Sleep Outcomes for Road, Rail, and Aircraft Noise

After reviewing individual studies in which the effect of road, rail, or aircraft noise on self-reported sleep outcomes was measured, the decision was made to focus on the 3 most common outcomes, the definitions of which are:

- Awakenings from sleep, which refers to the period after sleep onset and before the final awakening. They are defined as events where a subject wakes up from sleep, regains consciousness, and recalls the awakening in the next morning.
- The process of falling asleep, which is defined as the transition from wakefulness into sleep.
- Sleep disturbance refers to internal/external interference with sleep onset or sleep continuity (sleep maintenance).

Results from surveys that contained general questions about sleep and surveys that included questions specifically on how noise affects sleep were included in the review. The results for self-reported sleep disturbance were not reported in the literature in a consistent manner; therefore in order to conduct a meta-analysis, the authors of the individual papers reviewed were contacted in order to obtain the number of participants who reported each response alternative for 5 dB noise categories. This information was obtained for 30 studies, which were used to derive exposure-response relationships for the percent highly sleep disturbed for the different sleep outcome measures. We were unable to obtain data from five studies. Data for confounding variables was not obtained for any of the studies. The number of participants in these studies and sleep questions used are listed in Table 2, Table 3 and Table 4.

### 4.1. Statistical Analysis

For the meta-analysis, the noise metric used was the average outdoor A-weighted noise level (L_night_). All studies used this metric (although relative to different time periods), except for Bodin et al. (2015) who reported the average 24 h noise level (L_Aeq,24hr_) [66]. The L_Aeq,24hr_ was converted to L_night_ using linear equations between the two metrics that were derived based on the Swiss transportation noise map (sonBase). The equations used for road traffic and railway noise are:

(4) $$\mathrm{Road}\;\mathrm{Traffic}:{\;L}_{\mathrm{night}}=L_{\mathrm{Aeq},24h}-6.0\;\mathrm{dB}$$

(5) $$\mathrm{Railway}:{\;L}_{\mathrm{night}}=L_{\mathrm{Aeq},24h}-0.9\;\mathrm{dB}\;$$

For most studies the noise metric was predicted or measured at the most exposed façade of the dwelling, not the bedroom. The L_night_ levels assigned for all studies were the midpoint of the 5 dB categories. For open-ended noise exposure categories (e.g., <50 or >50) the noise level assigned was 2.5 dB above or below the category, for example for <50 dB the assigned value would be 47.5 dB.

The approach used in this meta-analysis is not the same as the approach used by Miedema and Vos (2007) [22], who previously developed exposure-response models relating the percent highly sleep disturbed for road, rail, and aircraft noise based on survey response data. In their analysis, the survey response data used was available at the individual response level. The response scales for the questions on sleep disturbance varied between the studies used in their analysis. In order to derive a combined model, they translated the response categories for each question to a scale of 0 to 100 by dividing 100 by the number of response choices and multiplying by the rank of the response choice. They modeled a cumulative distribution function based on the assigned scores and then calculated the percent of the population that was estimated to have a score of 72% or higher, which was the cutoff point they defined as highly sleep disturbed, for different L_night_ levels.

For this analysis, data was not obtained at the individual level, results were not always obtained for all response categories, and questions were included in which the frequency or the severity of sleep disturbance was reported. Therefore instead of modeling sleep disturbance as a continuous function, the probability of being highly sleep disturbed was modeled. A binary variable was created for highly sleep disturbed. Following previous conventions used for the ICBEN annoyance scale, for questions that used a 5 point or 11 point scale, and referred to the severity of sleep disturbance the top two and top three categories, respectively, were defined as highly sleep disturbed. For the few questions that referred to the frequency of symptoms, such as Halonen et al. (2010) [67], response alternatives for symptoms occurring three times or more per week were considered highly sleep disturbed. This criterion was used, as having difficulty sleeping at least three times per week for at least one month is considered a diagnostic criterion of insomnia [68]. For other response scales, the response alternatives that were considered highly sleep disturbed are highlighted in the tables.

One line of data was created for each study respondent. For example, if a study had 1000 respondents in the noise category with a 47.5 dB L_night_ midpoint, and 20% were classified as highly sleep disturbed, we generated 800 data lines with non-highly sleep disturbed respondents (binary outcome = 0) and 200 data lines with highly-sleep disturbed respondents (binary outcome = 1). Each data line also carried the mid-point of the 5 dB-wide L_night_ exposure category (data were requested from study PIs that way) and a unique identifier for each study. Random study effect logistic regression models with L_night_ as the only explanatory continuous variable were performed with the NLMIXED procedure in SAS (version 9.3, SAS Institute, Cary, NC, USA). This approach takes into account that respondents were clustered within studies, and the weight of a study increases with its sample size and thus precision. The fixed effect estimates reflect the average study (for a detailed discussion of differences in subject specific and population average modeling approaches see Section S3). The models are based on L_night_ levels between 40 and 65 dBA only. The reason for setting a lower limit of 40 dB is due to inaccuracies of predicting lower noise levels, and 65 dB was chosen for comparability between sources as aircraft noise levels did not exceed this level. Point estimates and 95% confidence intervals were generated with estimate statements in Proc NLMIXED. Analyses were performed separately for each noise source, type of sleep disruption, and whether the question referred specifically to how noise affects sleep. The odds ratios for all outcome measures and noise sources are in Table 5 and Table 6. We also calculated a combined estimate of high sleep disturbance across the different survey outcomes (falling asleep, awakenings, sleep disturbance). If a study asked questions on two or three of these outcomes, we averaged the results across outcomes within a study to prevent each subject contributing more than once to the analysis.

**Figure 7:**
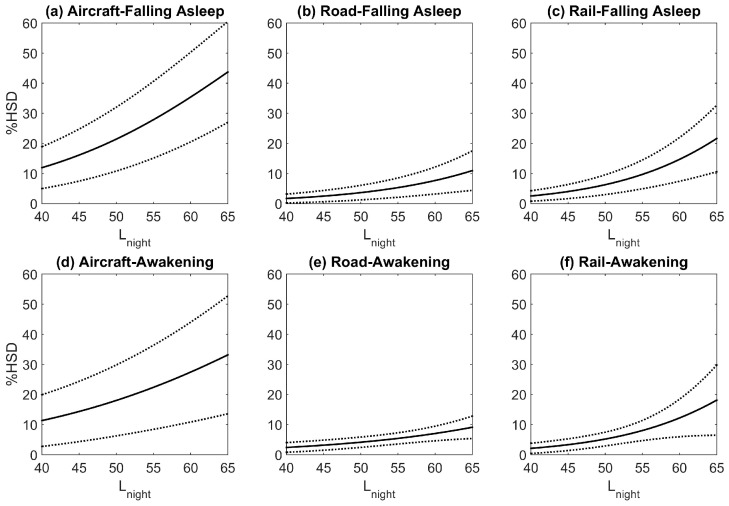
The percent highly sleep disturbed (HSD) based on responses to questions on awakenings or difficulty falling asleep for road, rail, and aircraft noise and for studies that asked about how noise affects sleep (black dashed lines: 95% confidence intervals). The number of studies and subjects contributing to the analyses can be found in Table 2, Table 3 and Table 4.

**Figure 8:**
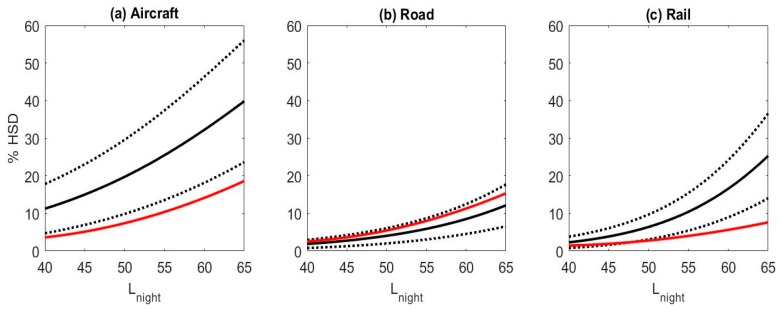
The percent highly sleep disturbed (HSD) based on responses to questions on awakenings, difficulty falling asleep, and sleep disturbance for road, rail, and aircraft noise (black dashed lines: 95% confidence intervals). The number of studies and subjects contributing to the analyses can be found in Table 2, Table 3 and Table 4. Red: Miedema and Vos (2007) [22] highly sleep disturbed exposure-response curves.

**Figure 9:**
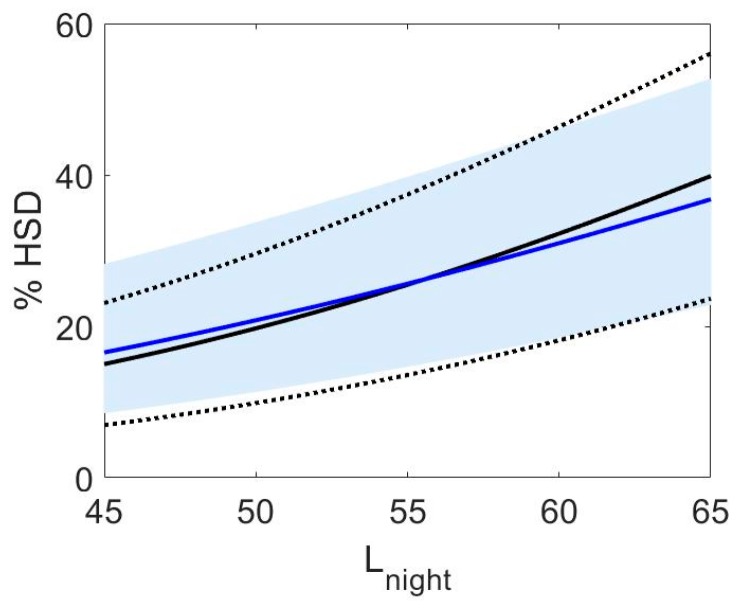
The percent highly sleep disturbed (HSD) based on responses to questions on awakenings, difficulty falling asleep, and sleep disturbance for aircraft noise (black dashed lines: 95% confidence intervals). The number of studies and subjects contributing to the analyses can be found in Table 2. Blue: Janssen and Vos (2009) [87] highly sleep disturbed exposure-response curve.

The exposure-response relationships for falling asleep and awakenings for studies that asked about how noise affects sleep are shown in Figure 7. The relationships are not shown individually for questions on sleep disturbance due to the low number of studies. The percent highly sleep disturbed for questions on difficulty falling asleep were higher than the percent highly sleep disturbed calculated based on questions on awakenings. Results for all questions were averaged within each study, and the exposure-response relationships for the combined estimates are shown in Figure 8. For comparison the Miedema and Vos [22] sleep disturbance exposure-response relationships are also shown in Figure 8. For road and rail noise, the percent of the population that was estimated to be highly sleep disturbed was approximately 2% for L_night_ levels of 40 dB. However for aircraft noise 10% of the population was estimated to be highly sleep disturbed for the same noise level. Janssen and Vos [87] derived an updated exposure response curve for the percent highly sleep disturbed for aircraft noise only. This update included studies used by Miedema and Vos that were conducted in the year 1996 or later, and 4 additional studies, two of which are included in this analysis, Brink et al. [76] and Schreckenberg et al. [75]. The aircraft noise exposure-response relationship developed in this analysis and the one derived by Janssen and Vos [87] is shown in Figure 9.

Second order polynomials were calculated based on the point estimates for the exposure-response relationships for awakenings, difficulty falling asleep, and the combined estimates for questions that asked about the noise source. The equations obtained are as follows (valid for an L_night_ range of 40–65 dB):

For questions on difficulty falling asleep:

(6) $$\mathrm{Aircraft}\;\%\mathrm{HSD}=16.3369-0.9663*L_{\mathrm{night}}+0.0214*\;{\left(L_{\mathrm{night}}\right)}^{2}$$

(7) $$\;\mathrm{Road}\;\%\mathrm{HSD}=19.3767-0.9263*L_{\mathrm{night}}+\;0.0122*{\left(L_{\mathrm{night}}\right)}^{2}$$

(8) $$\;\mathrm{Train}\;\%\mathrm{HSD}=44.4836\;-2.1324*L_{\mathrm{night}}+0.0273*{\left(L_{\mathrm{night}}\right)}^{2}$$

For questions on awakenings:

(9) $$\mathrm{Aircraft}\;\%\mathrm{HSD}=12.0411-0.5646*L_{\mathrm{night}}+0.0137*{\left(L_{\mathrm{night}}\right)}^{2}$$

(10) $$\mathrm{Road}\;\%\mathrm{HSD}=8.8986\;-0.4209*L_{\mathrm{night}}+\;0.0065*{\left(L_{\mathrm{night}}\right)}^{2}$$

(11) $$\mathrm{Train}\;\%\mathrm{HSD}=38.5819-1.8376*L_{\mathrm{night}}+0.0234*{\left(L_{\mathrm{night}}\right)}^{2}$$

For the combined estimates:

(12) $$\mathrm{Aircraft}\;\%\mathrm{HSD}=16.7885\;-0.9293*L_{\mathrm{night}}+0.0198\;*{\left(L_{\mathrm{night}}\right)}^{2}$$

(13) $$\mathrm{Road}\;\%\mathrm{HSD}=19.4312-0.9336*L_{\mathrm{night}}+0.0126\;*{\left(L_{\mathrm{night}}\right)}^{2}$$

(14) $$\mathrm{Train}\;\%\mathrm{HSD}=67.5406-3.1852*L_{\mathrm{night}}+0.0391*{\left(L_{\mathrm{night}}\right)}^{2}$$

**Figure 10:**
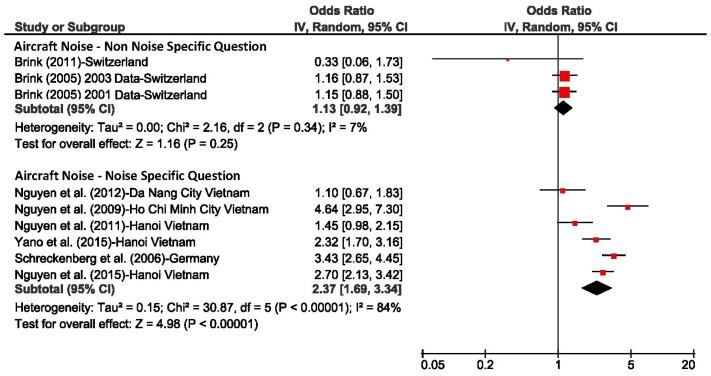
Meta-analysis on the effects of aircraft noise on self-reported sleep disturbance (combined estimate) based on Odds Ratios for a 10 dBA increase in L_night_ level for aircraft noise. The number of studies and subjects contributing to the analyses can be found in Table 2.

**Figure 11:**
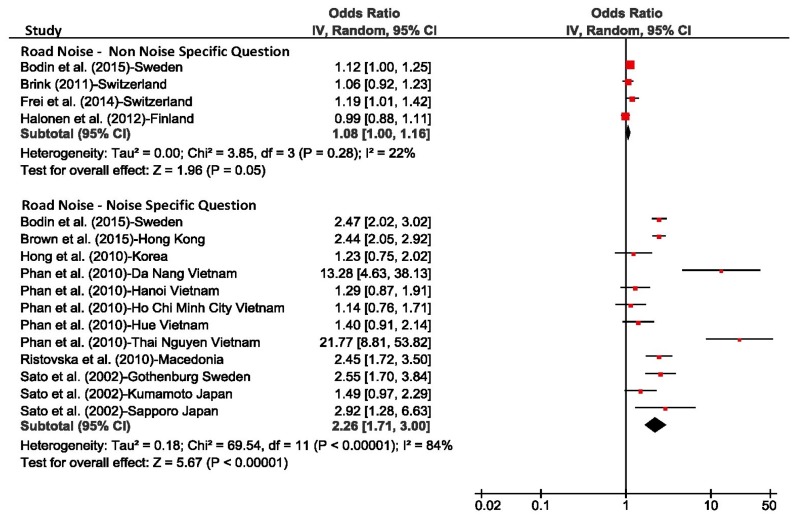
Meta-analysis on the effects of road noise on self-reported sleep disturbance (combined estimate) based on Odds Ratios for a 10 dBA increase in L_night_ level for road noise. The number of studies and subjects contributing to the analyses can be found in Table 3.

**Figure 12:**
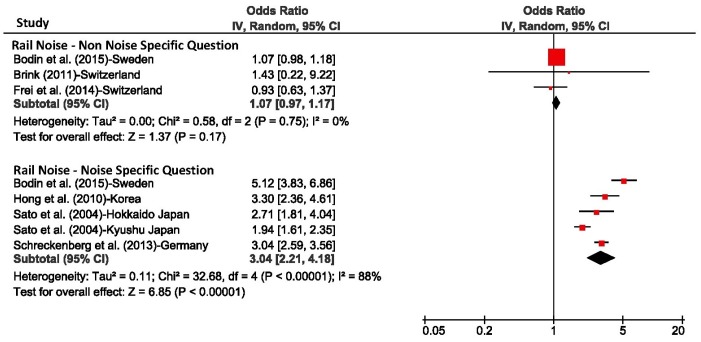
Meta-analysis on the effects of rail noise on self-reported sleep disturbance (combined estimate) based on Odds Ratios for a 10 dBA increase in L_night_ level for rail noise. The number of studies and subjects contributing to the analyses can be found in Table 4.

In addition to the analyses based on individual response data presented above, we also calculated the unadjusted odds ratio per 10 dBA increase in L_night_ for each individual study (using the combined estimate) and derived pooled estimates across studies for each transportation mode with the Review Manager Software (RevMan, Version 5.3, Copenhagen: The Nordic Cochrane Centre, The Cochrane Collaboration, 2014). L_night_ was treated as a continuous variable and its range was not restricted for calculating individual study estimates. The purpose of this analysis was primarily to assess the heterogeneity of the studies. The results are shown in Figure 10, Figure 11 and Figure 12. The small differences between pooled estimates provided in Table 5 and Table 6 and Figure 10, Figure 11 and Figure 12 are expected due to the different underlying methodological approaches (random study effect model estimate based on individual response data versus pooled estimate across individual study estimates).

The *I*^2^ values, a measure of variance across studies, was 84% for road and aircraft noise studies that mentioned the noise source in the sleep question, and was 88% for train noise which indicates there was high heterogeneity between studies. In contrast, for studies that did not refer to the noise source, the *I*^2^ values were 22% or lower, however the number of studies for these meta-analyses were low.

### 4.2. Additional Studies

Results from studies that were not included in the meta-analysis are listed in Table S1. The reason for exclusion of these studies include: the aggregated response data was not available and that the sleep question used had only a binary response choice. Our meta-analysis without these studies is unlikely to be biased in showing a positive association between noise level and percent highly sleep disturbed as only one study by Ohrstrӧm et al. (2010) [88] found no association between self-reported sleep disturbance and train noise. However if these studies were included in the meta-analysis they may have affected the magnitude of the effect that was found.

### 4.3. Conclusions

Noise is only one reason for sleep disturbance. There are many other external (e.g., temperature, humidity, light levels) and internal (e.g., sleep disorders, health conditions, bad dreams) causes. For this reason, odds ratios for sleep disturbance were calculated separately for those studies that did and did not ask about sleep disturbance, awakenings, or problems falling asleep relative to a specific noise-source. The odds ratios calculated for all noise sources and sleep outcomes were greater than 1 but not statistically significant when the noise source was not specifically mentioned in the question except in one case. However, odds ratios were much higher and mostly statistically significantly different from 1 when the noise source was mentioned in the question. This difference could be due to lack of adjustment for confounding factors in the analysis, such as age, gender, socio-economic status, and pre-existing sleep or health conditions. However, the context and wording of the questions can also bias the results. 

The surveys included in this meta-analysis consisted of both noise surveys and general health surveys which contained questions on sleep. Bodin et al. [89] examined whether response to questions on the effects of road traffic and train noise was dependent on the context of the survey, whether the survey was presented as a noise and health survey. The question on sleep asked how often sleep was disturbed. The percent of the population providing response alternatives at the end of the scale (i.e., “Every day” and “Never”) was the same when the questions were presented as a noise survey and when they were presented as a more general survey.

In the studies examined in this meta-analysis the type of questions asked were also different, with some studies referring specifically to how noise affects sleep while other studies contained more general sleep questions. Barker and Tarnopolsky (1978) [90] examined the difference in response to noise specific and non-noise specific questions in two groups of people exposed to high and low levels of aircraft noise. They asked two questions in their study, one question asked if participants had been nervous and irritable and the other asked if aircraft noise made them feel nervous or irritable. When the question did not refer to noise the percent reporting symptoms was not significantly different between the high noise and low noise exposure group. However there was a significant difference between the two exposure groups when the question referred to noise, which is consistent with the findings of our meta-analyses. For the studies used in this review, even when questions referred to the noise source and the same sleep outcome measure, there were additional differences in the specific wording, reference time frame, and response format of the sleep questions. For example, some studies referred to sleep disturbance during the past 12 months, others during the past month, and a few studies referred to single events or no time period at all. These differences could have all contributed to the high heterogeneity found between studies.

Despite the differences in sleep questions used, results were averaged across questions within studies to obtain combined estimates. These estimates were compared to the previous models developed by Miedema and Vos (2007) [22]. In contrast to their analysis our meta-analysis found that the percent highly sleep disturbed was greater for railway noise than for road noise. In addition, for both rail and aircraft noise the percent highly sleep disturbed was higher in this analysis than Miedema and Vos’s. This difference could be due to different methodologies used to derive the model. Also many of the studies included in this meta-analysis were conducted in Japan and Vietnam where the noise exposure and attitude towards noise may be different than in European countries. In addition, in Miedema and Vos’s analysis the questions referred to annoyance that occurred due to sleep disturbance for several of the studies, while in this analysis the questions were on the severity or frequency of sleep disturbance. Also, in the studies included on train noise in this analysis, more nighttime events were reported than in previous studies [86].

Another potential difference for the findings in this analysis and Miedema and Vos’s is that this analysis only contained studies published in the year 2000 or later. Recent updates to annoyance exposure-response curves have found an increase in annoyance although only for aircraft noise [91]. The higher reported sleep disturbance found in this analysis is also consistent with the updated exposure-response curve reported by Janssen and Vos [87] for aircraft noise which only included studies conducted in 1996 or later.

Limitations of the current meta-analysis include that L_night_ was predicted or measured at the most exposed façade only, and thus noise levels at the bedroom façade were unknown. The potential effect on the results is likely dependent on the noise source, and could be more important for the results for road and train noise but less for aircraft noise due to the directionality of the noise. Ultimately, this misclassification could result in a shift in the exposure-response curves for road and rail noise to the left, as noise levels in the bedroom are on average likely lower compared to the most exposed façade. Also two of the studies included in the meta-analysis did occur after a change in noise level. The Nguyen et al. [69] aircraft study occurred after the opening of a new terminal building. The average nighttime noise levels did increase for 9 of the 11 sites. However the mean increase in L_night_ was 2 dB; in addition there was a non-significant difference in the Odds Ratio when compared to the results from the Yano et al. [70] study that was conducted before the new terminal was opened. Therefore we included the data in the analysis. Brink et al. [76] conducted 2 surveys before and after a change in operations at Zurich airport, the results from both studies were included in the evidence review as the odds ratios for an increase in sleep disturbance for the two studies were not significantly different.

## 5. Wind Turbine Noise and Self-Reported and Actigraphy Measured Sleep Outcomes

### 5.1. Literature Review

Six studies were identified in the literature review in which the association between predicted A-weighted sound pressure levels of wind-turbine noise and self-reported measures of sleep disturbance were assessed. For three of the studies the questions asked how noise affects sleep. Two of the studies were conducted in Sweden [92,93] and one in the Netherlands [94]. For the two studies conducted in Sweden sleep disturbance was assessed using a binary question which asked whether sleep was disturbed by any noise source, while the study conducted in the Netherlands asked how often sleep was disturbed by any noise source with a frequency of at least once a month considered sleep disturbance. The odds ratios for sleep disturbance per 1 dB increase in the predicted A-weighted sound pressure level for all three studies was reported in Pedersen 2011 [48], the values transformed for a 10 dBA increase in noise level can be found in Table 7. For two of the studies a significant association was found between wind turbine noise levels and sleep disturbance. In addition, the Dutch study by Bakker et al. (2012) [94] reported a significant Odds Ratio for sleep disturbance when comparing individuals exposed to noise levels above 45 dBA to those exposed to noise levels less than 30 dBA (2.98, 95% CI: 1.35–6.60). However, in their structural equation model, they found that annoyance was the only factor that predicted sleep disturbance.

For the three remaining studies the effect of wind turbine noise on sleep was evaluated using questions that did not refer to noise. Pawlaczyk-Luszcynsa et al. [95] conducted a study in 2011 in Poland which included questions on different aspects of sleep including difficulty falling asleep. They found that the proportion of individuals reporting that they suffer from sleep disturbance at least a few times per week was significantly higher in individuals exposed to wind turbine noise levels of 40–45 dBA compared to those exposed to levels of 35–40 dBA (26% vs. 10.2%, *p* < 0.05). Kuwano et al. [96] examined self-reported insomnia in a study conducted in Japan. This study included both a noise exposed and control group. Insomnia was defined as having difficulty falling asleep, maintaining sleep, prematurely awakening, or having light sleep at least 3 times a week for any reason. The insomnia prevalence rate in the study was low, with 3.1% of participants exposed to 41–45 dB L_night_ and 2.7% of participants exposed to an L_night_ of greater than 45 dB reporting insomnia. Kuwano et al. also stratified their data according to those individuals who were noise sensitive or not noise sensitive and a significant association between insomnia and L_night_ was only found in the noise sensitive population, though this analysis is limited due to the very low insomnia prevalence rate in the study. Also in contradiction to this finding, Pedersen and Persson-Waye [92] found no association between noise sensitivity and reported sleep disturbance. Michaud [97] assessed subjective and objective measures of sleep for those exposed to predicted wind turbine noise levels of up to 46 dB in Canada. In total 1238 households completed subjective assessments which included the Pittsburgh Sleep Quality Index. No association was found between the mean value of PSQI and wind turbine noise levels or between the percent of participants with a score of 5 or higher and the noise levels. Michaud also evaluated whether individuals were highly sleep disturbed, and found no significant association with wind turbine noise levels.

**Figure 13:**
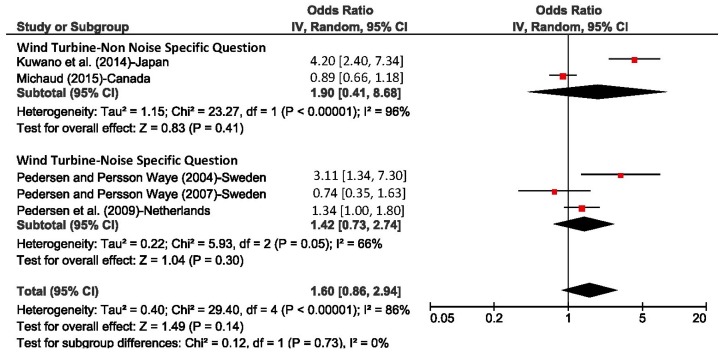
Meta-analysis on the effects of wind turbine noise on self-reported sleep disturbance based on Odds Ratios for a 10 dBA increase in A-weighted SPL level for wind turbine noise. The number of subjects contributing to the analyses can be found in Table 7.

A meta-analysis was conducted for five of the six studies based on the odds ratios for sleep disturbance for a 10 dBA increase in outdoor predicted SPL levels. The results are shown in Figure 13. The analysis was performed separately for questions that did and did not mention noise in the questions on sleep. The pooled odds ratio was 1.60 (95% CI: 0.86–2.94) which was statistically non-significant, there was also high heterogeneity between studies with an *I*^2^ value of 86%.

### 5.2. Conclusions

The results of the six identified studies that measured self-reported sleep disturbance are consistent, four of the studies found an association between wind turbine noise levels and increased sleep disturbance. However the evidence that wind turbine noise affects sleep is still limited. This finding is supported by other recent reviews on wind turbine noise and sleep disturbance [56,98,99]. Three of the studies referred to noise specifically in the questions which could have led to a bias in the results. Also while the results from four out of the six studies suggest that sleep disturbance due to wind turbine may occur when noise levels are above 40 or 45 dBA, for two of the studies less than ten percent of the participants were exposed to these higher noise levels. Therefore, it is difficult to make conclusions on populations exposed to these higher levels. In addition, noise levels were calculated using different methods and different noise metrics were reported in the studies. Pawlaczyk-Luszcynsa et al. [95] reported L_den_ levels which were obtained by adding a +4.7 dBA correction to the predicted sound pressure levels. In the Kuwano et al. [96] study wind turbine noise was measured at select locations, and then a logarithmic regression was performed between the measured noise levels and distance from the wind turbines. Noise levels for each participant were estimated based on the regression which could have led to misclassification. While noise level measurements were made to confirm noise predictions in a few studies, noise levels were never measured inside participant’s bedrooms. The audibility of wind turbine noise in bedrooms particularly when windows are closed is unknown. In the study by Pedersen and Persson Waye [92] all but two of 20 subjects that reported sleep disturbance slept with open windows.

Evidence is also limited as five of the six studies only obtained self-reported measures of sleep disturbance. There have been two studies which used actigraphy to evaluate sleep due to wind turbine noise. In a study by Lane [100] 13 individuals slept for five consecutive nights while wearing actigraphy devices. The sample size was too small to draw significant conclusions. Actigraphy was also used to evaluate sleep for multiple nights in a subsample of 654 participants in a study by Michaud [97]. They found no significant association between wind turbine noise levels and actigraphy measured outcomes, but predicted L_night_ levels did not exceed 46 dBA outside with an arithmetic mean of 35.6 dBA for the study population. Studies using both objective measures of sleep and noise exposure are still needed.

## 6. Hospital Noise

### 6.1. Literature Review

Seventeen studies were identified in which the effects of hospital noise on sleep were examined. Five were intervention studies in which quiet hours were implemented to reduce noise. While intervention studies are covered in another review, we included them here due to the low number of studies on hospital noise and sleep that were identified. Also it may be difficult to observe a wide variance in noise levels within a study in the same hospital ward without implementing an intervention. Of the non-intervention studies, nine examined the effect of noise on sleep in adult patients and three studies examined the effect on young children. Characteristics for all studies reviewed are shown in Table 8, Table 9 and Table 10. The study methodology was too diverse and prohibited us from doing a systematic meta-analysis. Of the studies in adults, four compared arousals measured with polysomnography to peaks in noise level. Aaron et al. (1996) [102] found a significant correlation between arousals and noise events which exceeded 80 dBA (*r* = 0.57, *p* = 0.0001) in a small study of six patients. However, in a study by Elliott et al. (2013) [103] which used a similar methodology but enrolled 53 patients, only a weak non-significant correlation between arousals and noise events was found (daytime measurements: *r* = 0.13; nighttime measurements *r* = 0.19). Freedman et al. (2001) [104] reported that 11.5% ± 11.8% of arousals in patients were due to noise events. Gabor et al. (2003) [105] examined sleep in both patients and healthy individuals who slept in the Intensive Care Unit and found that while 68.4% of arousals in healthy individuals were related to noise events only 17.5% of arousals in patients were. Three of the studies reviewed used actigraphy to evaluate measures of sleep duration and efficiency. Adachi et al. (2013) [106] found no association between hourly minimum noise levels and sleep duration. Missildine et al. (2010) [107] found no association between sleep efficiency and mean noise levels. However, Yoder et al. (2012) [108] did find that those exposed to the loudest tertile of average nighttime noise levels slept significantly less than those exposed to the quietest tertile.

Of the three studies identified that examined sleep in children, two of the studies, Corser (1996) [109] and Cureton-Lane and Fontaine (1997) [110], evaluated sleep subjectively using the Patient Sleep Behavior Observation Tool (Echols, 1968) [111] which describes patient behaviors that are related to 4 levels of cortical activity. Corser (1996) [109] found a small correlation between noise levels and observed sleep state (*r* = −0.20, *p* < 0.05) in infants (mean age 23.3 months). The observed sleep state though was more strongly correlated to behavioral indicators of pain (*r* = −0.27, *p* < 0.05) and caregiver activities (*r* = −0.30, *p* < 0.05). Similar results were found by Cureton-Lane and Fontaine [110]. In a probit analysis, noise was a significant predictor of sleep state in children (mean age 4.7 years). However, light levels and caregiver activity were also identified as significant predictors. Kuhn et al. [112,113] used both subjective and objective measures of sleep; the objective measurements included heart rate, blood pressure and respiration rate. They found that respiration rate significantly decreased during quiet sleep in pre-term infants when a noise event exceeded the background level by 10 dBA (−10.0 ± 12.5 breaths/min, *p* = 0.002).

Several of the studies examined whether interventions to reduce noise resulted in improved sleep. Dennis et al. (2010) [114] implemented a two hour quiet period during the day and night in which telephone volumes were decreased, caregiving activities were reduced, visiting hours were limited, and the staff were encouraged to interact quietly. During the day the implementation of quiet hours resulted in a 9 dB reduction in noise level (71.2 dB prior to the intervention, 62.2 dB during the intervention) while at night only a 1.4 dB reduction occurred. Sleep state was determined based on observation every 30 min. A significant Odds Ratio for being asleep was found when the intervention was implemented during the day (4.04, 97.5% CI 2.24–7.30) however, not when it was implemented during the night (0.96, 97.5% CI 0.41–2.24). Gardner et al. (2009) [115] implemented a quiet period during the daytime only and included both an experimental and control group. While they found a significant correlation between noise levels and the number of patients observed to be awake in the experimental group (*r* = 0.704, *p* ≤ 0.01) the correlation in the control group was weak (*r* = 0.24, *p* < 0.05). Therefore, it is unclear whether it was the reduction in noise level that resulted in more of the patients being observed asleep. Walder et al. (2000) [116] found results that were opposite to the previous studies, they found that sleep duration and the number of awakenings was greater after behavioral rules to reduce noise were implemented. However, the same patients did not take part before and after the intervention was implemented, also the number of patients enrolled was small. Contrary to previous studies, Thomas et al. (2012) [117] did not find an improvement in noise levels when sleep promoting measures were put into practice; however the noise levels were low, below 40 dB before the intervention. One intervention study was conducted with children. Duran et al. (2012) [118] conducted a study to examine whether preterm infants that wore earmuffs while in an incubator, which reduced noise levels by 7–12 dBA, had improved heart rate, respiration rate, and blood pressure, and subjective observations of sleep. They found that more infants were observed in a state of rest when wearing the earmuffs (87.5% with ear muffs, 29.4% without earmuffs). However no difference was found in the physiological measurements. Both subjective and objective measurements were recorded once every two hours.

### 6.2. Conclusions

Sleep quality in hospitals in general is low. Studies have found that sleep primarily consists of Stage 1 and 2 sleep with low or absent amounts of REM and slow-wave sleep [104,119]. In addition average sleep bouts of 20 min duration or less have been measured [107]. Sleep disturbance in hospitals can be caused by many factors including pain, medication, desynchrony with ventilation, care-giving activities, stress, unfamiliar environment, in addition to environmental factors such as light and noise levels. While noise is just one component, the average noise levels in the studies reviewed were high, with L_day_, L_night_, and L_eq,24hr_ primarily above 50 dBA [103,105,110], with several reporting noise levels exceeding 60 dBA [115,120,121].

Despite the high noise levels the quality of the evidence on the effect of noise on sleep is low. The results of 14 studies do indicate that noise is among the factors contributing to sleep disturbance in hospitals. The results from the four studies that used polysomnography indicate there is a weak correlation between EEG arousals and events of high noise level and that 10–20% of all arousals maybe associated with noise events. The results from studies using actigraphy measures of sleep however were contradictory with only one study finding a significant association between noise and sleep duration. In children, the study by Kuhn et al. [112,113] did find that increases in noise level affected physiological measures of pre-term infants. Also in two of the four studies, implementing quiet hours in adults, lower noise levels and improved sleep were found. The relationship between noise levels or signal-to-noise ratios and the likelihood of having a physiological reaction to the noise events though is unclear based on the studies reviewed.

Another limitation of the studies reviewed is that several only examined correlations and confounding factors were not adequately examined. A study by Park et al. (2014) [120] though did include several important confounders in their analysis. They measured subjective sleep quality using the Pittsburgh Sleep Quality Index [122] and found that sleep disturbance scores increased with mean daytime and nighttime noise levels even after controlling for age, gender, severity of disease, medication, and room-type. Additional factors that should be examined include mechanical ventilation and time in unit. The length of time spent in the hospital could be examined as a confounding variable or as an outcome measure as it may increase when there are higher noise levels.

## 7. Additional Sleep Outcome Measures

### 7.1. Cardiac and Blood Pressure Outcome Measures during Sleep in Adults

In this review, while several studies were identified in which electrocardiogram (ECG) measurements were performed [35,60,61,62] only two studies were identified in which the results on the effects of transportation noise on cardiac measures and blood pressure were reported. Haralabidis et al. (2008) [123] examined the effect of road and aircraft noise on heart rate and blood pressure measurements as part of the HYENA study. 140 subjects underwent 24 h ambulatory blood pressure measurements with heart rate, measurements were recorded every 15 min. Noise levels within the bedroom were also recorded. When aircraft events occurred a small but significant increase in both systolic and diastolic blood pressure was found for a 5 dB increase in indoor maximum noise level (systolic: 0.66 mmHg, 95% CI 0.33–0.98 and diastolic: 0.64 mmHg, 95% CI 0.37–0.90). For road traffic noise a small but significant increase in blood pressure was also found (systolic: 0.81 mmHg, 95% CI 0.46–1.16 and diastolic: 0.55 mmHg, 95% CI 0.26–0.83). Graham et al. (2009) [124] examined respiratory sinus arrhythmia and pre-ejection period in 36 subjects exposed to road and rail noise. Respiratory sinus arrhythmia was considered an index for cardiac parasympathetic tone and pre-ejection period was considered an index of cardiac sympathetic tone. No significant association was found between pre-ejection period and the average indoor noise level during the sleep period. A significant decrease of the log of respiratory sinus arrhythmia with noise level was found, with age as a significant covariate. This finding suggests that noise exposure may lead to decreased parasympathetic tone.

### 7.2. Motility Measured Sleep Outcomes in Adults

Eight studies were identified in which motility was measured (see Table 11). Four of the studies examined the probability of having a motility reaction due to single noise events. In a study by Passchier-Vermeer et al. (2002) [64] 418 individuals that lived near Schiphol airport wore actigraphs continuously for 11 days. They found a significant increase in motility reaction with the indoor maximum noise level (L_AS,max_) of aircraft events. The estimated probability of a motility reaction was less than 1% for events of 40 dB, and was greater than 4% for events of 60 dB. In 2007, Passchier-Vermeer et al. conducted a second study to examine the effect of road and rail noise on measures of motility. The study included 262 participants who wore actigraphs for 5 consecutive nights. They found that motility and motility onset increased with noise level, and that railway noise did not have a greater effect on motility than road traffic noise. Hong et al. (2006) [125] also used actigraphs to evaluate sleep in 12 subjects exposed to railway noise. They found slightly higher probability of reaction then found in the Passchier-Vermeer et al. [64] study. Lercher et al. (2010) [126] used seismosomnography [127] to measure movement in individuals exposed to rail noise. In a linear regression, for the probability of motility, the coefficient for L_Amax_ was significant (0.04 per dB, 95% CI 0.01–0.07, *p* < 0.01).

For the remaining 4 studies, actigraphy derived sleep parameters for the entire night were compared to average noise levels. Ohrstrӧm et al. (2006) [128] conducted a study using actigraphy in both children and their parents. No clear exposure-response relationship was found between mean activity, wake episodes, and sleep latency and predicted L_Aeq,24hr_ for the parents. Frei et al. (2014) [84] did not find a significant decrease in sleep duration with predicted outdoor L_night_ levels. However, sleep efficiency was found to decrease with L_night_ even after adjusting for several confounding variables including gender, age, education, and body mass index. Unlike the two previous studies Pirrera et al. (2014) [129] recorded noise levels within the bedroom of participants. The study consisted of two groups, 23 individuals that lived in an area with high levels of road traffic and 22 individuals that lived in a more quiet area. There was a 10 dB difference in the mean outdoor L_Aeq_ (measured during the participant’s time in bed period) between the high and low noise group, however there was not a significant difference in the indoor L_Aeq_ levels between the two groups. Therefore although individuals in the high noise group spent less time in bed (high noise group: 433 min, quiet group: 451 min), there was no significant difference found in sleep onset latency, wake after sleep onset, or sleep efficiency. Griefahn et al. (2000) [130], similar to the other studies mentioned, found no association between road and rail noise levels and motility. The results from motility studies are therefore conflicting in that there is evidence from 4 of the 8 studies that for single-events there is an increase in movement. On the other hand, there is not consistent evidence that sleep parameters descriptive of the entire night are affected by noise.

### 7.3. Sleep Disturbance in Children

The results from sleep studies in children have suggested that they are less likely to awaken to noise events than adults, with a difference in sensitivity of approximately 10 dBA [132]. However, despite being less sensitive, children are still considered a vulnerable group due to their developmental state and also because of the difference in their sleep patterns. Children have earlier bedtimes and longer sleep durations than adults, which may overlap with periods of high traffic not accounted for by metrics such as L_night_. 

Five studies on the effects of road, rail, and aircraft noise on sleep in children published since 2000 were identified as part of this review (see Table 12). Ohrstrӧm et al. (2006) [128] conducted a study to examine the effect of road traffic noise on sleep in both adults and children. They conducted a main study which included a questionnaire and a more detailed study in which subjects filled out sleep logs and wore actigraphs for 4 days. The children in the study were between the ages of 9–12 years. In the main study a small yet significant decrease in self-reported sleep quality with increasing predicted outdoor L_Aeq,24hr_ levels was found. However, no relationship between outdoor noise levels and actigraphy measured sleep parameters was found. Lercher et al. (2013) [133] found a small but significant relationship between road and rail noise (L_den_) and a sleep disturbance index which was based on responses to questions on sleep onset, maintaining sleep, and tiredness in 3rd and 4th grade students. The variance explained by the models though was small. Ising and Ising (2002) [134] obtained self-reported measures of sleep for 56 children between the ages of 7–13. Noise levels were measured in the children’s bedroom. They found that those children exposed to higher C-weighted maximum noise levels were more likely to report problems sleeping. Tiesler et al. (2013) [135] examined the relationship between predicted noise levels and self-reported sleep disturbance in children that were part of a population-based birth-cohort study called LISAplus. Data on sleep was available for 287 children and the mean age of children in the cohort studies was 10 years. They found a significant relationship between noise levels (L_night_) at the least exposed façade and sleeping problems (OR 1.79, 95% CI 1.10–2.92) and difficulty falling asleep (OR 1.96, 95% CI 1.16–3.32) after controlling for a number of confounding variables including gender, age, and parental education. However, a significant relationship was not found for noise levels at the most exposed façade. They also found that those children reporting sleep problems were more likely to report emotional symptoms although this was not significantly related to noise level. Stansfeld et al. (2010) [136] examined whether self-reported sleep disturbance in children in the Munich study mediated the relationship between aircraft noise and cognitive performance. However, they did not find an effect.

The results of four of the studies suggest that noise may lead to poorer self-reported sleep in children. Additional studies are needed though to determine the effect of noise on both subjective and objective measures of sleep in children. Also more studies are needed to examine whether nighttime noise exposure may contribute to attention deficits, emotional or behavioral problems, or reduced cognitive performance.

## 8. Summary of Available Evidence

A summary of the evidence for different noise sources and sleep outcome measures is shown in Table 13. For road, rail, and aircraft noise the focus of this review was to conduct a re-analysis for polysomnography measured awakenings and a meta-analysis for self-reported sleep outcome measures. The quality of the evidence that transportation noise causes cortical awakenings is moderate. The two studies reviewed were conducted using a similar methodology and exposure-response relationships were developed for all three transportation modes. The results from the analysis consistently indicate that a 10 dBA increase in the indoor maximum noise level is associated with an Odds Ratio for awakenings or sleep stage changes to Stage 1 of 1.3 or higher.

For self-reported sleep outcome measures, the quality of the evidence is dependent on the wording of the questions. When individuals were asked whether road, rail, or aircraft noise affected sleep a significant increase in the odds of being highly sleep disturbed was found for a 10 dBA increase in outdoor L_night_ levels for all sources. However no significant increase was found when the noise source was not mentioned. Because the dose-response relationships between Lnight and percentage highly sleep disturbed were statistically significant and showed Odds Ratios > 2, for both road and rail noise, we upgraded our GRADE assessment from very low to moderate quality for studies using questions that did mention noise as the cause (see Table 13, and Tables S3 and S4). However, we downgraded to very low quality for studies using the respondents’ answers to questions that did not mention the noise source, due to inadequate adjustment for confounding and imprecision due to the low number of studies. This suggests that for self-reported measures it is annoyance or attitude to the nighttime noise that may be driving the increase of reported sleep disturbance outcomes with L_night_ level. However, whether or not the question is reflective of sleep disturbance or attitude to nighttime noise both are important endpoints. For the other outcome measures and noise sources, we were not able to derive pooled odds ratios. 

## 9. Conclusions

This review demonstrates effects of traffic noise on objectively measured sleep physiology and on subjectively assessed sleep disturbance (including sleep quality, problems falling asleep, and awakenings during the night). The evidence for other sources of noise (e.g., hospital noise, wind turbine noise) is conflicting or only emerging and did not allow for the derivation of exposure-response functions. There is biologic plausibility that chronic night time exposure to relevant levels of noise can contribute to negative health consequences like cardiovascular disease. Although recent epidemiological studies have shown stronger relationships of nocturnal noise exposure [34] with negative health consequences compared to daytime noise exposure, studies directly investigating the link between acute noise-induced sleep disturbance and long-term health consequences are missing and not an easy undertaking. However, disturbed sleep has immediate next-day consequences (e.g., increased sleepiness, impaired cognitive performance) that may increase the risk for errors and accidents, and thus sleep deserves protection from noise even in the absence of a direct link to long-term health consequences. The exposure-response functions provided in this report can be used to assess the degree of noise-induced sleep disturbance. It is plausible that preventing acute effects of noise on sleep will likely also prevent long-term negative health consequences.

## Figures and Tables

**Table 1:** Odds Ratios and 95% confidence intervals for sleep stage transitions to awake or Stage 1 for road, rail, and aircraft noise for a 10 dBA increase in the indoor maximum noise level (L_AS,max_). Number of subjects contributing to the analysis: Road = 94, Aircraft = 61, Rail = 33.

Odds Ratio per 10 dBA (L_AS,max_)	Road (STRAIN and DEUFRAKO)	Aircraft (STRAIN)	Rail (DEUFRAKO)	Combined Estimate (Based on Road, Rail, and Aircraft)
Unadjusted	1.36 (1.19–1.55)	1.35 (1.22–1.50)	1.35 (1.21–1.52)	1.35 (1.25–1.45)
Adjusted for Age and Gender	1.36 (1.19–1.55)	1.35 (1.21–1.50)	1.34 (1.19–1.50)	1.28 (1.21–1.36)
Adjusted for Age, Gender, Day of the Week, and Time From Sleep Onset	1.32 (1.15–1.50)	1.32 (1.19–1.47)	1.34 (1.19–1.51)	1.29 (1.21–1.36)

**Table 2:** Studies on aircraft noise and self-reported sleep disturbance (* general health survey, ^+^ noise survey). Studies modeled the noise levels except where indicated. Response alternatives contributing to the calculation of the percent Highly Sleep Disturbed are in bold.

Study	*N*	Country	Sleep Disturbance Questions	Noise Metric (Range for Obtained Data)
**Falling Asleep (Total *N* = 6368)**				
^+^ Nguyen et al. (2015) [69]	1095	Hanoi, Vietnam	In daily life, when an airplane passes by, at what degree are you disturbed in the following cases: When it makes it difficult for you to fall asleep? Not at all, Slightly, Moderately, **Very, Extremely**	L_night_, 22:00–6:00, measured (1 week) (37.5–57.5)
^+^ Yano et al. (2015) [70]	780	Hanoi, Vietnam		L_night_, 22:00–6:00, measured (1 week) (37.5–57.5)
^+^ Nguyen et al. (2012) [71]	512	Da Nang City, Vietnam		L_night_, 22:00–6:00, measured (1 week) (37.5–52.5)
^+^ Nguyen et al. (2010) [72] Nguyen et al. (2011) [73]	805	Hanoi, Vietnam		L_night_, 22:00–6:00, measured (1 week) (37.5–52.5)
^+^ Nguyen et al. (2009) [74]	868	Ho Chi Minh City, Vietnam		L_night_, 22:00–6:00, measured (1 week) (42.5–62.5)
^+^ Schreckenberg et al. (2009) [75]	2308	Germany	How much has aircraft noise in the last 12 months disturbed falling asleep? Not at all, Slightly, Moderately, **Very, Extremely**.	L_night_, 22:00–6:00 (37.5–57.5)
**Awakenings (Total *N* = 4054)**				
^+^ Nguyen et al. (2015) [69]	1093	Hanoi, Vietnam	In daily life, when an airplane passes by, to what degree are you disturbed in the following cases: When you are awakened in your sleep? Not at all, Slightly, Moderately, **Very, Extremely**.	L_night_, 22:00–6:00, measured (1 week) (37.5–57.5)
^+^ Yano et al. (2015) [70]	776	Hanoi, Vietnam		L_night_, 22:00–6:00, measured (1 week) (37.5–57.5)
^+^ Nguyen et al. (2012) [71]	511	Da Nang City, Vietnam		L_night_, 22:00–6:00, measured (1 week) (37.5–52.5)
^+^ Nguyen et al. (2010) [72] Nguyen et al. (2011) [73]	804	Hanoi, Vietnam		L_night_, 22:00–6:00, measured (1 week) (37.5–52.5)
^+^ Nguyen et al. (2009) [74]	870	Ho Chi Minh City, Vietnam		L_night_, 22:00–6:00, measured (1 week) (42.5–62.5)
**Sleep Disturbance (Total *N* = 2309)**				
^+^ Schreckenberg et al. (2009) [75]	2309	Germany	How much has aircraft noise in the last 12 months disturbed sleeping during the night? Not at all, Slightly, Moderately, **Very, Extremely**.	L_night_, 22:00–6:00 (37.5–57.5)
**Falling Asleep-Noise source not specified in sleep questions (Total *N* = 2978)**				
^+^ Brink et al. (2005) [76] 2001 Study	1528	Switzerland	How often do you have the following symptoms: Problems falling asleep? Never, Rarely, Sometimes, Often, **Very Often, Always**	L_night_, 22:00–6:00 (27.5–62.5)
^+^ Brink et al. (2005) [76] 2003 Study	1450			L_night_, 22:00–6:00 (27.5–62.5)
**Awakenings-Noise source not specified in sleep questions (Total *N* = 2978)**				
^+^ Brink et al. (2005) [76] 2001 Study	1528	Switzerland	How often do you have the following symptoms: Problems with sleeping through? Never, Rarely, Sometimes, Often, **Very Often, Always**	L_night_, 22:00–6:00 (27.5–62.5)
^+^ Brink et al. (2005) [76] 2003 Study	1450			L_night_, 22:00–6:00 (27.5–62.5)
**Sleep Disturbance-Noise source not specified in sleep questions (Total *N* = 195)**				
* Brink (2011) [77]	195	Switzerland	During the last 4 weeks, have you suffered from any of the following disorders or health problems? Difficulty in sleeping or insomnia? Not at all, Somewhat, **Very Much**.	L_night_, 22:00–6:00 (32.5–52.5)
				L_night_, 22:00–6:00 (32.5–52.5)

**Table 3:** Studies on road noise and self-reported sleep disturbance (* general health survey, ^+^ noise survey). Studies modeled the noise levels except where indicated. Response alternatives contributing to calculation of the percent Highly Sleep Disturbed are in bold.

Study	*N*	Country	Sleep Disturbance Questions	Noise Metric (Range for Obtained Data)
**Falling Asleep (Total *N* = 10,212)**				
^+^ Bodin et al. (2015) [66]	2444	Sweden	Do you experience any of the following because of road traffic noise? Difficulties falling asleep. Never, Sometimes, **Often**.	L_Aeq_, 24 h (37.5–62.5)
^+^ Sato et al. (2002) [78]	1302	Gothenburg, Sweden	Does the road traffic noise cause the following conditions? Difficulty to fall asleep? No, Little Disturbed, Rather Disturbed, **Very Disturbed**.	L_night_, 22:00–7:00, measured (1 night) (42.5–72.5)
	814	Kumamoto, Japan		L_night_, 22:00–7:00, measured (1 night) (47.5–77.5)
	779	Sapporo, Japan		L_night_, 22:00–7:00, measured (1 night) (52.5–67.5)
^+^ Phan et al. (2010) [79] Shimoyama et al. (2014) [80]	1471	Hanoi, Vietnam	How much are you disturbed in falling asleep by road traffic? Not at all, Slightly, Moderately, **Very, Extremely**.	L_night_, 22:00–6:00, measured (1 night) (62.5–77.5)
	1458	Ho Chi Minh City, Vietnam		L_night_, 22:00–6:00, measured (1 night) (67.5–77.5)
	481	Da Nang, Vietnam		L_night_, 22:00–6:00, measured (1 night) (57.5–67.5)
	682	Hue, Vietnam		L_night_, 22:00–6:00, measured (1 night) (52.5–72.5)
	781	Thai Nguyen, Vietnam		L_night_, 22:00–6:00, measured (1 night) (52.5–67.5)
**Awakenings (Total *N* = 10177)**				
^+^ Bodin et al. (2015) [66]	2438	Sweden	Do you experience any of the following because of road traffic noise? You wake up? Never, Sometimes, **Often**.	L_Aeq_, 24 h (37.5–62.5)
^+^ Sato et al. (2002) [78]	1291	Gothenburg, Sweden	Does the road traffic noise cause the following conditions? Awakening? No, Little Disturbed, Rather Disturbed, **Very Disturbed**.	L_night,_ 22:00–7:00, measured (1 night) (42.5–72.5)
	819	Kumamoto, Japan		L_night_, 22:00–7:00, measured (1 night) (47.5–77.5)
	779	Sapporo, Japan		L_night_, 22:00–7:00, measured (1 night) (52.5–67.5)
^+^ Phan et al. (2010) [79] Shimoyama et al. (2014) [80]	1454	Hanoi, Vietnam	How much are you disturbed by awakening during nighttime by road traffic? Not at all, Slightly, Moderately, **Very, Extremely**.	L_night_, 22:00–6:00, measured (1 night) (62.5–77.5)
	1460	Ho Chi Minh City, Vietnam		L_night_, 22:00–6:00, measured (1 night) (67.5–77.5)
	479	Da Nang, Vietnam		L_night_, 22:00–6:00, measured (1 night) (57.5–67.5)
	680	Hue, Vietnam		L_night_, 22:00–6:00, measured (1 night) (52.5–72.5)
	777	Thai Nguyen, Vietnam		L_night_, 22:00–6:00, measured (1 night) (52.5–67.5)
**Sleep Disturbance (Total *N* = 9901)**				
^+^ Brown et al. (2015) [81]	8841	Hong Kong	How much is your sleep disturbed by road traffic noise? 11 point scale used from 0 (not disturbed at all) to 10 (extremely disturbed) **(8, 9, 10 HSD)**	L_night_ (42.5–67.5)
^+^ Hong et al. (2010) [82]	550	Korea	How much have you been disturbed in your sleep by road traffic noise at night when you are sleeping in your house over the last 12 months? 11 point scale used from 0 (not disturbed at all) to 10 (extremely disturbed) **(8, 9, 10 HSD)**	L_night_, 22:00–7:00 (50.0–73.0)
^+^ Ristovska et al. (2009) [83]	510	Macedonia	Do you think that your sleep was disturbed due to night-time noise or noise events during the night in the last twelve months and more? Not at all, Very little, Moderate, **High, Very High**.	L_night_, 23:00–7:00, measured (2 nights) (42.5–62.5)
**Falling Asleep–Noise source not specified in sleep questions (*N* = 10,545)**				
^+^ Bodin et al. (2015) [66]	2520	Sweden	Do you have problems falling asleep? Rarely/never, A few times per month, A few times a week, **Almost every day**	L_Aeq_, 24 h (37.5–62.5)
* Halonen et al. (2012) [67]	6793	Finland	How many times during the past 4 weeks have you had the following symptoms? Difficulty falling asleep? Never, 1 per month, 1 per week, **2–4 per week, 5–6 per week, Nearly every night**.	L_night_, 22:00–7:00 (42.5–57.5)
* Frei et al. (2014) [84]	1232	Switzerland	How often does it happen, that you cannot fall asleep well? Never, Rarely, Sometimes, **Often**.	L_night_, 22:00–6:00 (27.5–62.5)
**Awakenings–Noise source not specified in sleep questions (*N* = 10,603)**				
^+^ Bodin et al. (2015) [66]	2519	Sweden	Do you wake up at night? Rarely/never, A few times per month, A few times a week, **Almost every day**	L_Aeq_, 24 h (37.5–62.5)
* Halonen et al. (2012) [67]	6853	Finland	How many times during the past 4 weeks have you had the following symptoms? Frequently waking up during the night. Never, 1 per month, 1 per week, **2–4 per week, 5–6 per week, nearly every night.**	L_night_, 22:00–7:00 (42.5–57.5)
* Frei et al. (2014) [84]	1231	Switzerland	How often does it happen, that you wake up at night multiple times? Never, Rarely, Sometimes, **Often**.	L_night_, 22:00–6:00 (27.5–62.5)
**Sleep Disturbance-Noise Source not specified in sleep questions (*N* = 9474)**				
* Brink (2011) [77]	8245	Switzerland	During the last 4 weeks, have you suffered from any of the following disorders or health problems? Difficulty in sleeping, or insomnia? Not at all, Somewhat, **Very Much**	L_night_, 22:00–6:00 (32.5–77.5)
* Frei et al. (2014) [84]	1229	Switzerland	How often does it happen that your sleep is restless? Never, Rarely, Sometimes, **Often**	L_night_, 22:00–6:00 (27.5–62.5)

**Table 4:** Studies on railway noise and self-reported sleep disturbance (* general health survey, ^+^ noise survey). Studies modeled the noise levels except where indicated. Response alternatives contributing to calculation of the percent Highly Sleep Disturbed are in bold.

Study	*N*	Country	Sleep Disturbance Questions	Noise Metric (Range for Obtained Data)
**Falling Asleep (Total *N* = 6520)**				
^+^ Bodin et al. (2015) [66]	2342	Sweden	Do you experience any of the following because of railway noise? Difficulties falling asleep? Never, Sometimes, **Often**	L_Aeq_, 24h (37.5–62.5)
^+^ Sato et al. (2004) [85]	1418	Hokkaido, Japan	How much are you disturbed in falling asleep by train passing? Not at all, Slightly, Moderately, **Very, Extremely**.	L_night_, 22:00–7:00, measured (27.5–62.5)
	1562	Kyushu, Japan		L_night_, 22:00-7:00, measured (27.5-72.5)
^+^ Schreckenberg (2013) [86]	1198	Germany	To what extent have the following outcomes of railway noise occurred in the past 12 months? Railway noise disturbs when falling asleep. Not at all, Slightly, Moderately, **Very, Extremely**.	L_night_, 22:00–6:00 (42.5-82.5)
**Awakenings (Total *N* = 5311)**				
^+^ Bodin et al. (2015) [66]	2344	Sweden	Do you experience any of the following because of railway noise? You wake up? Never, Sometimes, **Often**	L_Aeq_, 24h (37.5–62.5)
^+^ Sato et al. (2004) [85]	1418	Hokkaido, Japan	How much are you disturbed by awakening during nighttime by train passing? Not at all, Slightly, Moderately, **Very, Extremely**.	L_night_, 22:00–7:00, measured (27.5–62.5)
	1549	Kyushu, Japan		L_night_, 22:00–7:00, measured (27.5–72.5)
**Sleep Disturbance (Total *N* = 1809)**				
^+^ Hong et al. (2010) [82]	610	Korea	How much have you been disturbed in your sleep by railway noise at night when you are sleeping in your house over the last 12 months? 11 point scale used from 0 (not disturbed at all) to 10 (extremely disturbed) **(HSD 8, 9, 10)**	L_night_, 22:00–7:00 (47.1–70)
^+^ Schreckenberg (2013) [86]	1199	Germany	To what extent have the following outcomes of railway noise occurred in the past 12 months? Railway disturbs when sleeping during the night. Not at all, Slightly, Moderately, **Very, Extremely**.	L_night_, 22:00–6:00 (42.5–82.5)
**Falling Asleep- Noise source not specified in sleep questions (Total *N* = 3808)**				
^+^ Bodin et al. (2015) [66]	2576	Sweden	Do you have problems falling asleep? Rarely/never, A few times per month, A few times a week, **Almost every day**	L_Aeq_, 24 h (37.5–62.5)
* Frei et al. (2014) [84]	1232	Switzerland	How often does it happen, that you cannot fall asleep well? Never, Rarely, Sometimes, **Often**.	L_night_, 22:00–6:00 (27.5–57.5)
**Awakening-Noise source not specified in sleep questions (Total *N* = 3806)**				
^+^ Bodin et al. (2015) [66]	2575	Sweden	Do you wake up at night? Rarely/never, A few times per month, A few times a week, **Almost every day**	L_Aeq_, 24 h (37.5–62.5)
* Frei et al. (2014) [84]	1231	Switzerland	How often does it happen, that you wake up at night multiple times? Never, Rarely, Sometimes, **Often**.	L_night_, 22:00–6:00 (27.5–57.5)
**Sleep Disturbance-Noise source not specified in sleep questions (*N* = 5914)**				
* Brink (2011) [77]	4685	Switzerland	During the last 4 weeks, have you suffered from any of the following disorders or health problems? Difficulty in sleeping, or insomnia? Not at all, Somewhat, **Very Much**	L_night_, 22:00–6:00 (32.5–77.5)
* Frei et al. (2014) [84]	1229	Switzerland	How often does it happen that your sleep is restless? Never, Rarely, Sometimes, **Often**	L_night_, 22:00–6:00 (27.5–57.5)

**Table 5:** Unadjusted Odds Ratio for the percent highly sleep disturbed for road, rail, and aircraft noise for questions on falling asleep, awakenings, and sleep disturbance for a 10 dBA increase in L_night_. L_night_ was treated as a continuous variable from 40 to 65 dBA. Results are for questions that asked how noise affects sleep. Bold font reflects statistically significant results at *p* < 0.05. The combined estimate is based on all sleep questions. The number of subjects contributing to the analyses can be found in Table 2, Table 3 and Table 4.

	Number of Studies	Odds Ratio per 10 dBA	95% Confidence Interval
**Aircraft Noise**			
Falling Asleep	6	**2.00**	**1.68–2.41**
Awakenings	5	**1.72**	**1.31–2.27**
Sleep Disturbance	1	**2.05**	**1.64–2.56**
Combined Estimate	6	**1.94**	**1.61–2.33**
**Road Noise**			
Falling Asleep	8	**2.63**	**1.86–3.73**
Awakening	8	**1.75**	**1.24–2.47**
Sleep Disturbance	3	**2.21**	**1.52–3.20**
Combined Estimate	12	**2.13**	**1.82–2.48**
**Rail Noise**			
Falling Asleep	4	**2.57**	**1.87–3.53**
Awakening	3	**2.54**	**1.49–4.33**
Sleep Disturbance	2	4.10	0.69–24.41
Combined Estimate	5	**3.06**	**2.38–3.93**

**Table 6:** Unadjusted Odds Ratio for the percent highly sleep disturbed for road, rail, and aircraft noise for questions on falling asleep, awakenings, and sleep disturbance for a 10 dBA increase in L_night_. L_night_ was treated as a continuous variable from 40 to 65 dBA. Results are for questions that did not refer to noise in the questions. Bold font reflects statistically significant results at *p* < 0.05. The combined estimate is based on all sleep questions. The number of subjects contributing to the analyses can be found in Table 2, Table 3 and Table 4.

	Number of Studies	Odds Ratio per 10 dBA	95% Confidence Interval
**Aircraft Noise**			
Falling Asleep	2	1.10	0.73–1.57
Awakenings	2	0.89	0.66–1.22
Sleep Disturbance	1	4.70	0.41–53.62
Combined Estimate	3	1.17	0.54–2.53
**Road Noise**			
Falling Asleep	3	1.03	0.77–1.38
Awakenings	3	1.01	0.81–1.25
Sleep Disturbance	2	1.43	0.36–5.59
Combined Estimate	4	1.09	0.94–1.27
**Rail Noise**			
Falling Asleep	2	**2.02**	**1.44–2.83**
Awakenings	2	1.12	0.90–1.39
Sleep Disturbance	2	1.23	0.85–1.80
Combined Estimate	3	1.27	0.89–1.81

**Table 7:** Characteristics of studies on self-reported measures of sleep disturbance and wind turbine noise. Odds ratios for sleep disturbance are listed.

Reference	Country	N	N (>40 dBA)	Noise Exposure	Confounding Variables Adjusted for in the Statistical Analysis	Odds Ratio per 10 dBA (95% CI)	Odds Ratio Relative to Reference (95% CI)
Pedersen and Persson Waye (2004) [92]	Sweden	351	25	Predicted A-weighted SPL	Age, gender	3.11 (1.34–7.30)	Reference: <35 dBA >35 dBA: 4.72 (0.27–82.97)
Pedersen and Persson Waye, (2007) [93]	Sweden	754	20	Predicted A-weighted SPL	Age, gender	0.74 (0.35–1.63)	NA
Pedersen et al. (2009) [101] Bakker et al. (2012) [94]	Netherlands	725	159	Predicted A-weighted SPL	Age, gender, economic benefits	1.34 (1.00–1.80)	Reference <30 dBA >45 dBA: 2.98 (1.35–6.60)
Kuwano et al. (2014) [96]	Japan	747 (332 Controls)	260	L_night_ (22:00-6:00)	Age, gender	4.20 (2.40–7.34)	Reference: <35 dBA 41–45 dBA: 5.55 (1.12–27.47) >46 dBA: 4.79 (0.64–35.70)
Michaud (2015) [97]	Canada	1238	234	Predicted A-weighted SPL	None	0.89 (0.66–1.18)	NA
Pawlaczyk-Luszcynsa et al. (2014) [95]	Poland	156	90	L_den_	None	NA	Reference: 35–40 dBA 40–45 dBA: 2.74 (1.08–6.97)

**Table 8:** Characteristics of studies on hospital noise and sleep in adults.

Reference	N	Age	Hospital Unit	Noise Measurement	Subjective Measure	Objective Measure	Outcome
Aaron et al. 1996 [102]	6	66.8 ± 2.8 years	Intensive and Intermediate Respiratory Care Unit	SPL every minute	NA	Polysomnography	Correlation (*r* = 0.57, *p* = 0.0001) between number of arousals (between 22:00–6:00) and SPL peaks ≥ 80 dB
Adachi et al. 2013 [106]	118	65.0 ± 11.6 years	General Medicine	Hourly L_min_, L_eq_, L_max_	Karolinska Sleep Log	Actigraphy	Multivariate linear and logistic regressions: No significant association between L_min_ tertiles and sleep duration, Karolinska Sleep Quality, or noise complaints
Elliott et al. 2013 [103]	53	60.1 ± 20.0 years	Intensive Care Unit	L_Aeq_ and L_Cpeak_ levels logged every second	Richards Campbell Sleep Questionnaire	Polysomnography	Weak correlation between arousal indices and number of sound peaks > 80 dB (day *r* = 0.13, night *r* = 0.19)
Gabor et al. 2003 [105]	13 Patients: 7 Control: 6	Patients: 56. 7± 19.2 years Controls: 23–65 years	Intensive Care Unit	SPL	NA	Polysomnography	17.5 ± 11.2% (Patients) and 68.4 ± 11.1% (Control Subjects) of arousals were associated with a sound event greater than 10 dB over background
Freedman et al. 2001 [104]	22	61 ± 16 years	Intensive Care Unit	SPL every minute	NA	Polysomnography	11.5 ± 11.8% of arousals and 26.2 ± 24.8% of awakenings was due to environmental noise
Hsu et al. 2010 [121]	40	54. 5± 14.5 years	Cardiac Surgical Unit	SPL every second	Questions on insomnia	Heart rate and blood pressure every 5 min	Correlation between insomnia and noise level, L_eq_ (*r* = 0.09), L_max_ (*r* = 0.24), L_min_ (*r* = −0.03).
Missildine et al. 2010 [107]	48	79 years	Medical Unit	SPL levels (23:00–7:00)	Richards Campbell Sleep Questionnaire	Actigraphy	For those subjects with less than 300 minof sleep, 59% were exposed to nighttime noise levels ≥ 40 dBA. In a multiple regression for sleep efficiency, the coefficient for median noise level was not significant (β = −0.671, *p* = 0.836).
Park et al. 2014 [120]	103	60 ± 14.8 years	Internal Medicine	Leq	Pittsburgh Sleep Quality Index	NA	Sleep disturbance scores increased with mean daytime and nighttime levels (β = 0.2; 95% CI = 0.09–0.53 for daytime; β = 0.12; 95% CI = 0.07–0.36 for nighttime). Controlled for age, gender, severity of disease, medication, and room type.
Yoder et al. 2012 [108]	106	66.0 ± 12 years	General Medicine	L_min_, L_eq_, L_max_	Pittsburgh Sleep Quality Index	Actigraphy	Patients exposed to the loudest tertile of average nighttime noise levels slept significantly less (−76 min, 95% CI −134 to −18 min, *p* = 0.01) than patients exposed to the lowest tertile of noise.

**Table 9:** Characteristics of studies on hospital noise and sleep in children.

Study	N	Age	Hospital Unit	Measure of Noise	Subjective Measure	Objective Measure	Outcome
Corser 1996 [109]	12	23.3 ± 6.1 months	Pediatric Intensive Care Unit	SPL every 5 min	Patient Sleep Behavior Observation Tool used to identify sleep state every 5 min 19:00–7:00	NA	Correlation between observed sleep state and noise (*r* = −0.2043, *p* < 0.05).
Cureton-Lane and Fontaine 1997 [110]	9	4.7 ± 3.5 years	Pediatric Intensive Care Unit	SPL every 5 min	Patient Sleep Behavior Observation Tool used to identify sleep stage every 5 min from 20:00–6:00	NA	Noise was a significant predictor of sleep state in probit analysis (*p* < 0.001). Light levels and contact with staff were also significant predictors.
Kuhn et al. 2013 [112] Kuhn et al. 2012 [113]	26	28 weeks (median)	Neonatal Intensive Care Unit	Classified sound peaks: those exceeding the previous level by more than 5 dBA	Prechtl’s observational rating system for defining arousal states.	Heart Rate, Respiratory Rate and SaO_2_	Average percent awakened due to classified sound peaks was 33.8% (95% CI: 24–37%). For control periods without sound peaks average percent awakened was 11.7% (95% CI: 6.2–17.1%). For sound peaks 10–15 dBA above background a significant decrease in respiration rate (−10 ± 12.5 breath/min, *p* = 0.002) during quiet sleep was found.

**Table 10:** Characteristics of intervention studies on hospital noise and sleep in adults and children.

Study	N	Age	Hospital Unit	Invention	Measure of Noise	Subjective Measure	Objective Measure	Outcome
Dennis et al. 2010 [114]	50 Day: 35 Night: 15	Day: 55.5 ± 14.4 years Night: 52.9 ± 16.3 years	Neuro-Intensive Care Unit	Implemented 2 h quiet period during the day and night	SPL collected 6 times a day over a period of 5 s before, after and during the quiet time hours	Sleep Observation Tool: seven observations made per subject	NA	Odds Ratio (97.5% CI) observed asleep: Day: 4.04 (2.24–7.30) Night: 0.96 (0.41–2.24)
Duran et al. 2012 [118]	20	30.0 ± 2.2 weeks	Neonatal Intensive Care Unit	Infants wore earmuffs that decreased noise levels by 7–12 dBA for 2 days	Measurements made every 2 h during an 8 h period	Anderson Behavioral State Scoring System. Measurements made every 2 h during an 8 h period	Blood pressure, heart rate, respiration rate, body temperature, and oxygen saturation. Measurements made every 2 h during an 8 h period	For the two conditions (with and without earmuffs): No difference was observed in physiological measures. 87.5% of infants with earmuffs observed asleep, 29.4% of infants without earmuffs observed asleep
Gardner et al. 2009 [115]	293 Experimental: 137 Control Group: 156	Experimental Group: 56.4 ± 19.1 years Control Group: 50.5 ± 19.4 years	Orthopedic Unit	Implemented quiet hours	Daily SPL	Observed Sleep State	NA	Correlation between mean SPL levels and patients found to be awake: Experimental: (*r* = 0.704, *p* < 0.01) Control group: *r* = 0.243, *p* < 0.05)
Thomas et al. 2012 [117]	95 Phase 1: 32 Phase 2: 33 Phase 3: 30	Phase 1: 49 ± 1 years Phase 2: 43 ± 3 years Phase 3: 46 ± 3 years	Neurological Unit	Study had 3 phases with measured noise levels Phase 2: Sleep promoting rules	SPL between 20:00–8:00	Questions on sleep quality, sleep quantity	NA	Intervention did not result in a reduction in noise level. The median noise levels were: Phase 1: 38.6 dB, Phase 2: 40.6 dB, Phase 3: 43.5 dB
Walder et al. 2000 [116]	17 Before Guidelines: 9 After Guidelines: 8	Before Guidelines: 62.5 ±16.5 years After Guidelines: 57.8 ±15.9 years	Surgical Intensive Care Unit	Implemented behavioral rules	SPL, every 1 s between 23:00–5:00.	Nurses estimated the patient’s sleep duration and the number of awakenings.	NA	Sleep duration was shorter, and the number of awakenings higher when the behavioral rules were implemented.

**Table 11:** Characteristics of studies that evaluated sleep based on measures of motility.

Reference	*N*	Noise Source	Noise Metric	Outcome
Hong et al. (2006) [125]	12	Rail	L_Amax_ indoor	Exposure-response between probability of motility and indoor L_Amax_. A higher probability of motility than in previous aircraft noise studies was found.
Frei et al. (2014) [84]	119	Road	L_night_, 22:00–6:00, outdoor, most exposed facade	Decrease in sleep efficiency (percent) with outdoor L_night_. Coefficients for random subject intercept linear regression: 30–40 dB: 0.20 (95% CI −1.21, 1.60), 40–55 dB: −0.85 (95% CI −2.42, 0.71), >55 dB: −4.06 (−6.78, −1.35)
Griefahn et al. (2000) [130]	377	Road and Rail	Indoor and outdoor whole night and individual event noise levels	No significant effect of noise on sleep parameters found.
Lercher et al. (2010) [126]	8	Rail	L_Amax_ indoor	Coefficient for L_Amax_, in a linear regression for the probability of motility reaction was significant. (0.04, 95% CI 0.01–0.07, *p* < 0.01)
Ohrstrӧm et al. (2006) [128]	79	Road	L_Aeq,24hr_ outdoor, most exposed facade	No significant effect of noise on sleep parameters was found.
Passchier-Vermeer et al. (2002) [64]	418	Aircraft	L_Amax_ indoor	Exposure-response relationship between motility and indoor L_Amax._
Passchier-Vermeer et al. (2007) [131]	262	Road and Rail	L_Amax_ indoor	Significant noise metric coefficient when comparing probability of motility reaction to an estimated indoor L_Amax_ level. Motility reaction was greater when there was higher levels of background noise.
Pirrera et al. (2014) [129]	45	Road	L_Aeq_ indoor	No significant difference in indoor average noise levels was found despite differences in outdoor noise level. No significant difference in time in bed, total sleep time, sleep latency, wake after sleep onset, or sleep efficiency was found.

**Table 12:** Characteristics of studies that evaluated sleep in children.

Reference	Age	*N*	Confounding Variables Adjusted for in the Statistical Analysis	Noise Source	Noise Metric	Outcome
Ising and Ising (2002) [134]	7–13 years	56	Age, gender, social status	Road	L_Cmax_ Indoors	Significant correlation between L_Cmax_ and awakenings during sleep and problems to fall asleep
Lercher et al. (2013) [133]	8–11 years	1251	Gender, health status, and mother’s education	Road and Rail	L_den_ Outdoor most exposed facade	L_den_ was a significant predictor of self-reported sleep, but not when adjusted for sound perception score
Ohrstrӧm et al. (2006) [128]	Mean 10.9 years (range 9–12.9)	160 (survey) 79 (actigraphy)	None	Road	L_Aeq,24h_ Outdoor most exposed facade	Decrease in self-reported mean sleep quality (0–10) < 55 dB: 8.6, 55–59 dB: 8.2, 60–64 dB: 8.2, >64 dB: 8.1. No association between actigraphy measured sleep parameters and noise level
Tiesler et al. (2013) [135]	10.1 ± 2.2 years	287	Gender, age, parental education level, mother’s age at birth, television/computer usage, single parent status, sleeping alone, and orientation of the window	Road	L_night_ Outdoors, least exposed facade	Reporting any sleep problems: OR: 1.79 (95% CI 1.10–2.92) Reporting problems falling asleep: OR 1.96 (95% CI 1.16–3.32)

**Table 13:** Summary of findings.

Sleep Outcomes	Noise Source	Number of Participants (Studies)	Quality of Evidence	Noise Metric	Odds Ratio per 10 dBA Increase (95% CI)
Cortical Awakenings in Adults	Road	94 (2)	⊕⊕⊕Ο Moderate There was evidence of dose-response	Indoor L_AS,max_	1.36 (1.19–1.55)
	Rail	33 (1)	⊕⊕⊕Ο Moderate There was evidence of dose-response	Indoor L_AS,max_	1.35 (1.21–1.52)
	Aircraft	61 (1)	⊕⊕⊕Ο Moderate There was evidence of dose-response	Indoor L_AS,max_	1.35 (1.22–1.50)
Self-Reported Sleep Disturbance in Adults (Noise Source Specified)	Road	20,120 (12)	⊕⊕⊕Ο Moderate There was evidence of dose-response	Outdoor L_night_	2.13 (1.82–2.48)
	Rail	7133 (5)	⊕⊕⊕Ο Moderate There was evidence of dose-response	Outdoor L_night_	3.06 (2.38–3.93)
	Aircraft	6371 (6)	⊕⊕⊕Ο Moderate There was evidence of dose-response	Outdoor L_night_	1.94 (1.61–2.33)
Self-Reported Sleep Disturbance in Adults (Noise Source Not Specified)	Road	18,850 (4)	⊕ΟΟΟ Very Low Confounding factors not accounted for in analysis, Imprecision low number of studies	Outdoor L_night_	1.09 (0.94–1.27)
	Rail	8493 (3)	⊕ΟΟΟ Very Low Confounding factors not accounted for in analysis, Imprecision low number of studies	Outdoor L_night_	1.27 (0.89–1.81)
	Aircraft	3173 (3)	⊕ΟΟΟ Very Low Confounding factors not accounted for in analysis, Imprecision low number of studies	Outdoor L_night_	1.17 (0.54–2.53)
Motility Measures of Sleep in Adults	Road, Rail, Aircraft	1320 (8)	⊕⊕ΟΟ Low Single event analysis indicates dose-response	L_Amax_ and L_Aeq_	Not estimated
Self-Report and Motility Measured Sleep Disturbance in Children	Road, Rail, Aircraft	1754 (4)	⊕ΟΟΟ Very Low Inconsistency in results, small number of studies	Varied across studies	Not estimated
Self-Reported Sleep Disturbance in Adults	Wind Turbine Noise	3971 (6)	⊕ΟΟΟ Very Low Inconsistency in results and imprecision due to small sample sizes at highest noise levels	Outdoor A-weighted SPL	1.60 (0.86–2.94)
All Sleep Outcome Measures	Hospital Noise	964 Adults/67 Children (13 Adults/4 Children)	⊕ΟΟΟ Very Low Inconsistency in results and imprecision due to small sample sizes	Varied across studies	Not estimated

## Supplementary Materials

The following are available online at http://www.mdpi.com/1660-4601/15/3/519/s1, Table S1: Characteristics and outcomes of studies not included in the meta-analysis for self-reported sleep outcomes, Table S2: GRADE Table for the quality of evidence of noise from road, rail, and aircraft noise and cortical awakenings in adults, Table S3: GRADE Table for the quality of evidence of noise from road, rail, and aircraft noise and self-reported sleep disturbance in adults (noise source specified), Table S4: GRADE Table for the quality of evidence of noise from road, rail, and aircraft noise and self-reported sleep disturbance in adults (noise source not specified), Table S5: GRADE Table for the quality of evidence of noise from road, rail, and aircraft noise and motility measures of sleep in adults, Table S6: GRADE Table for the quality of evidence of noise from road, rail, and aircraft noise and self-report and motility measured sleep disturbance in children, Table S7: GRADE Table for the quality of evidence of noise from wind turbines associated with effects on sleep, Table S8: GRADE Table for the quality of evidence of noise from hospitals associated with effects on sleep, Table S9: Model coefficients for the random study effect logistic regression model (Mixed) and the GEE model for the percent Highly Sleep Disturbed due to Aircraft noise, Table S10: Model coefficients for the random study effect logistic regression model (Mixed) and the GEE model for the percent Highly Sleep Disturbed due to Road noise, Table S11: Model coefficients for the random study effect logistic regression model (Mixed) and the GEE model for the percent Highly Sleep Disturbed due to Train noise, Table S12: Model coefficients for the random subject effect logistic regression model (Mixed) and the GEE model for the probability of a sleep stage change to wake or S1 for Aircraft noise, Table S13: Model coefficients for the random subject effect logistic regression model (Mixed) and the GEE model for the probability of a sleep stage change to wake or S1 for Road noise, Table S14: Model coefficients for the random subject effect logistic regression model (Mixed) and the GEE model for the probability of a sleep stage change to wake or S1 for Train noise. Table S15: Percent Highly Sleep Disturbed for road, rail, and aircraft noise for the logistic regression models shown in Figure 8. Figure S1: Percent Highly Sleep Disturbed. Random study effect logistic regression (gray) and GEE regression (black) with 95% confidence intervals (dashed lines); Figure S2: Probability of a sleep stage change to wake or S1. Random subject effect logistic regression (gray) and GEE regression (black) with 95% confidence intervals (dashed lines); Table S16: Criteria used to rate the bias of individual studies; Table S17: Bias ratings for studies on noise from road, rail, and aircraft noise and cortical awakenings in adults; Table S18: Bias ratings for studies on road, rail, and aircraft noise and self-report sleep disturbance; Table S19: Bias ratings for studies on wind turbine noise; Table S20: Bias ratings for studies on hospital noise and sleep in adults; Table S21: Bias ratings for studies on hospital noise and sleep in children; Table S22: Bias ratings for studies on hospital noise studies that had interventions; Table S23: Bias ratings for studies on noise from road, rail, and aircraft noise and cardiac and blood pressure outcomes; Table S24: Bias ratings for studies on noise from road, rail, and aircraft noise and actigraphy outcomes; Table S25: Bias ratings for studies on noise from road, rail, and aircraft noise and children’s sleep; Table S26: Bias ratings for studies that were not included in the meta-analysis of self-reported sleep outcomes for road, rail, and aircraft noise.
