# Supplementary material for: WHO Environmental Noise Guidelines for the European Region: A Systematic Review on Environmental Noise and Effects on Sleep
Source: Int J Environ Res Public Health. 2018 Mar 14;15(3):519. doi: 10.3390/ijerph15030519 (PMC5877064; doi:10.3390/ijerph15030519)
Supplement: Supplementary file 1 [file ijerph-15-00519-s001.pdf]

International Journal of Environmental Research and Public Health

Supplemental Material

WHO Environmental Noise Guidelines for the European Region: A Systematic Review on Environmental Noise and Effects on Sleep

Mathias Basner * and Sarah McGuire

S1. Excluded Studies from the Meta-analysis of Self-Reported Sleep Outcomes for Road, Rail, and Aircraft Noise

The studies listed in Table S1 were excluded from the analysis performed in section 4 either because the question had a binary scale only, or because data was not available for inclusion in the review.

Table S1. Characteristics and outcomes of studies not included in the meta-analysis for self-reported sleep outcomes.

Study	N	Country	Noise Source	Sleep Questions	Confounding Variables Adjusted for in the Statistical Analysis	Noise Metric (Outdoor)	Outcome	Reason for Exclusion
Aasvang et al. (2008) [1]	1349	Norway	Rail	Awakenings due to rail noise. Yes/No. Difficulties falling asleep due to rail noise. Yes/No	Age, gender, income, education, duration of residence, noise sensitivity, type of bedroom window, duration of residence, pass by frequency of trains	L_{night}, bedroom façade	Increase in OR with noise level. Falling Asleep OR-Reference <40 dBA:<br>≥65 dBA 13.75 (95% CI 1.60-118.1)<br>Awakenings: OR-Reference 40-44 dBA:<br>60-64 dB: 3.6 (95% CI 1.69-7.63)<br>≥65 dB: 7.13 (95% CI 3.1-16.37)	Binary response choice
Bluhm et al. (2004) [2]	657	Sweden	Road	Does traffic noise lead to any of the following nuisances/disturbances. Difficulties falling asleep? Waking up? There is no noise, Yes often, Yes Sometimes, No never.	None	$L_{Aeq,24hr}$	Report of sleep disturbance often<br>3.9% > 50 dBA, 1.2% <50 dBA<br>Report of sleep disturbance sometimes<br>23.6 % > 50 dBA, 12.8% <50 dBA	Data not available
Bristow and Wardman (2003) [3]	187	UK	Aircraft	Sleep disturbed by Aircraft? Yes/No	None	L_{night} (22:00-6:00)	OR for 10 dBA increase: 1.515 (95% CI 0.979-2.343)	Binary response choice
Wardman et al. (2012) [4]	562	UK	Aircraft	Does noise from aircraft wake you up? Yes/No.	None	L_{night} (22:00-6:00)	OR for 10 dBA increase: 2.355 (95% CI 1.830-2.030)	Binary response choice

Fyhri and Aasvang (2010) [5]	3117	Norway	Road	Awakenings due to traffic noise. Yes/No.<br>Difficulties falling asleep due to traffic noise. Yes/No	Gender, age, noise sensitivity, annoyance, education	L_{night}	In a structural equation model annoyance was a strong predictor for individuals reporting problems sleeping (path estimate 0.94).	Binary response choice
Griefahn et al. (2000) [6]	1600	Germany	Road<br>Rail	Questions not specified in report.	None	L_{night} (22:00-6:00)	Reported sleep disturbance for road noise was approximately 0.5-1 point higher on a 5 point scale than rail noise for the same noise level.	Data not available
Jakoljević et al. (2006) [7]	339	Serbia	Road	Difficulty falling asleep: Not at all, Mostly not, Mostly yes, Very much<br>Sleep quality: Very bad, Bad, Variable, Good, Excellent	Age, gender, noise sensitivity, extroversion, neuroticism	L_{eq} (based on measurements)	OR for participants >65 dB (Reference <55 dB):<br>Difficulty falling asleep: 2.7 (95% CI 1.3-5.8)<br>Poor sleep quality: 3.0 (95% CI 1.1-7.9)	Noise measurements and low sample size may affect comparability to other studies.
Ohrström, Skånberg et al. (2006) [8]	956	Sweden	Road	Difficulty falling asleep, awakenings, and disturbed sleep quality were evaluated in terms of how often (never, sometimes, often) and how much (slightly, moderately, much)	Window position	L_{night}, bedroom façade	Difficulties in falling asleep, awakening, sleep quality showed a similar increase with noise level. Difficulties in falling sleep increased from 7% (37-41 dB) to 31 % (57-61 dB) when windows were closed. When windows were open at the highest noise level, sleep disturbance increased by 10-15%.	Data not available
Ohrström et al. (2010) [9]	974	Sweden	Road and Rail	Difficulty falling asleep, awakenings, and disturbed sleep quality were evaluated in terms of how often (never, sometimes, often) and how much (slightly, moderately, much)	Window location and position	L_{night}	Reported sleep disturbance was greater for road than railway noise. For windows closed,<br>Road noise: % reporting disturbed sleep increased from 9% (<45 dB) to 30 % (55-59 dB).<br>No increase with noise level for railway noise was found.	Data not available
Stošić et al. (2009) [10]	911	Serbia	Road	Difficulty falling asleep. Not at all, Generally no, Generally yes, Very much.	NA	L_{eq} (based on measurements)	Significant difference in difficulty falling asleep (%) (p <0.001):<br>>45 dB: 36.90 %<br>≤45 dB: 7.40 %<br>Significant difference in reports of awakenings (%) (p <0.001):<br>>45 dB: 27.6 %<br>≤45 dB: 6.9%	Data not available

S2. Grade Tables

Table S2. GRADE Table for the quality of evidence of noise from road, rail, and aircraft noise and cortical awakenings in adults.

**Domains**	**Criterion**	**Assessment**	**Downgrading**
**Start Level**	**Longitudinal = high; others = low**	All cross-sectional	**Low quality**
1. Study Limitations	Majority of studies low quality	Low number of studies but of high quality. Risk of selection bias.	No downgrade
2. Inconsistency	Conflicting results; high I^2	Consistent results, I^2 not assessed.	No downgrade
3. Directness	Direct comparison; same PECO	Yes, same PECO	No downgrade
4. Precision	Confidence interval contains 25% harm or benefit	Confidence intervals contain 25% harm	No downgrade
5. Publication Bias	Funnel plot indicates	Not able to assess	No downgrade
**Overall judgment**			**Low quality**
6. Dose-response	Significant trend	Yes	Upgrade
7. Magnitude of effect	RR > 2	Not observed	No upgrading
8. Confounding adjusted	Effect in spite of confounding working towards the nil	Adjusted for Age, Gender, Day of the Week, and Time From Sleep Onset.	No upgrading
**Overall Judgement**			**Moderate quality**

Table S3. GRADE Table for the quality of evidence of noise from road, rail, and aircraft noise and self-reported sleep disturbance in adults (noise source specified).

**Domains**	**Criterion**	**Assessment**	**Downgrading**
**Start Level**	**Longitudinal = high; others = low**	Majority cross-sectional studies	**Low quality**
1. Study Limitations	Majority of studies low quality	All with high risk of information bias	Downgrade one level
2. Inconsistency	Conflicting results; high I^2	High I^2 between studies (84-88%)	Downgrade one level
3. Directness	Direct comparison; same PECO	Yes, same PECO	No downgrade
4. Precision	Confidence interval contains 25% harm or benefit	All CI narrower than 25%	No downgrade
5. Publication Bias	Funnel plot indicates	Not assessed	No downgrade
**Overall judgment**			**Very low quality**
6. Dose-response	Significant trend	Yes	Upgrade
7. Magnitude of effect	RR > 2	OR > 2 for road and rail	Upgrade
8. Confounding adjusted	Effect in spite of confounding working towards the nil	Not observed	No upgrading
**Overall Judgement**			**Moderate quality**

Table S4. GRADE Table for the quality of evidence of noise from road, rail, and aircraft noise and self-reported sleep disturbance in adults (noise source not specified).

**Domains**	**Criterion**	**Assessment**	**Downgrading**
**Start Level**	**Longitudinal = high; others = low**	Majority cross-sectional studies	**Low quality**
1. Study Limitations	Majority of studies low quality	All with high risk of information bias	Downgrade one level
2. Inconsistency	Conflicting results; high I^2	I^2 between studies (0-22%)<br>(Low number of studies)	No downgrade
3. Directness	Direct comparison; same PECO	Yes, same PECO	No downgrade
4. Precision	Confidence interval contains 25% harm or benefit	CI wider than 25%	Downgrade one level
5. Publication Bias	Funnel plot indicates	Not assessed	No downgrade
**Overall judgment**			**Very low quality**
6. Dose-response	Significant trend	Non-significant	No upgrading
7. Magnitude of effect	RR > 2	Not observed	No upgrading
8. Confounding adjusted	Effect in spite of confounding working towards the nil	Not observed	No upgrading
**Overall Judgement**			**Very low quality**

Table S5. GRADE Table for the quality of evidence of noise from road, rail, and aircraft noise and motility measures of sleep in adults.

**Domains**	**Criterion**	**Assessment**	**Downgrading**
**Start Level**	**Longitudinal = high; others = low**	All cross-sectional studies	**Low quality**
1. Study Limitations	Majority of studies low quality	Medium risk of selection and information bias.	No downgrade
2. Inconsistency	Conflicting results; high I^2	I^2 not assessed, conflicting results between studies that examined single event reactions and whole night sleep outcome measures.	No downgrade
3. Directness	Direct comparison; same PECO	Yes, same PECO	No downgrade
4. Precision	Confidence interval contains 25% harm or benefit	Unable to assess for narrative review	No downgrade
5. Publication Bias	Funnel plot indicates	Unable to assess for narrative review	No downgrade
**Overall judgment**			**Low quality**
6. Dose-response	Significant trend	Significant trends found in literature for single event reaction analysis	No upgrading
7. Magnitude of effect	RR > 2	Unable to assess for narrative review	No upgrading
8. Confounding adjusted	Effect in spite of confounding working towards the nil	Not observed	No upgrading
**Overall Judgement**			**Low quality**

Table S6. GRADE Table for the quality of evidence of noise from road, rail, and aircraft noise and self-report and motility measured sleep disturbance in children.

**Domains**	**Criterion**	**Assessment**	**Downgrading**
**Start Level**	**Longitudinal = high; others = low**	Majority cross-sectional	**Low quality**
1. Study Limitations	Majority of studies low quality	Majority of studies used questionnaires, studies suffer from information bias.	Downgrade one level
2. Inconsistency	Conflicting results; high I^2	I^2 not assessed, conflicting results, small number of studies.	Downgrade one level
3. Directness	Direct comparison; same PECO	Yes, same PECO	No downgrade
4. Precision	Confidence interval contains 25% harm or benefit	Unable to assess for narrative review	No downgrade
5. Publication Bias	Funnel plot indicates	Unable to assess for narrative review	No downgrade
**Overall judgment**			**Very low quality**
6. Dose-response	Significant trend	Not observed	No upgrading
7. Magnitude of effect	RR > 2	Unable to assess for narrative review	No upgrading
8. Confounding adjusted	Effect in spite of confounding working towards the nil	Not observed	No upgrading
**Overall Judgement**			**Very low quality**

Table S7. GRADE Table for the quality of evidence of noise from wind turbines associated with effects on sleep.

**Domains**	**Criterion**	**Assessment**	**Downgrading**
**Start Level**	**Longitudinal = high; others = low**	All cross-sectional studies	**Low quality**
1. Study Limitations	Majority of studies low quality	High risk of bias	Downgrade one level
2. Inconsistency	Conflicting results; high I^2	High I^2 between studies (86%)	Downgrade one level
3. Directness	Direct comparison; same PECO	Yes, same PECO	No downgrade
4. Precision	Confidence interval contains 25% harm or benefit	CI wider than 25%	Downgrade one level
5. Publication Bias	Funnel plot indicates	Not assessed	No downgrade
**Overall judgment**			**Very low quality**
6. Dose-response	Significant trend	Non-significant	No upgrading
7. Magnitude of effect	RR > 2	Not observed	No upgrading
8. Confounding adjusted	Effect in spite of confounding working towards the nil	Not observed	No upgrading
**Overall Judgement**			**Very low quality**

Table S8. GRADE Table for the quality of evidence of noise from hospitals associated with effects on sleep.

Domains	Criterion	Assessment	Downgrading
**Start Level**	**Longitudinal = high; others = low**	Majority cross-sectional	**Low quality**
1. Study Limitations	Majority of studies low quality	High risk of bias. In 8 out of 15 studies sleep state was subjectively observed.	Downgrade one level
2. Inconsistency	Conflicting results; high I^2	I^2 not assessed, narrative review only	No downgrade
3. Directness	Direct comparison; same PECO	Yes, same PECO	No downgrade
4. Precision	Confidence interval contains 25% harm or benefit	Unable to assess for narrative review	No downgrade
5. Publication Bias	Funnel plot indicates	Unable to assess for narrative review	No downgrade
**Overall judgment**			**Very low quality**
6. Dose-response	Significant trend	Not observed	No upgrading
7. Magnitude of effect	RR > 2	Unable to assess for narrative review	No upgrading
8. Confounding adjusted	Effect in spite of confounding working towards the nil	Not observed	No upgrading
**Overall Judgement**			**Very low quality**

S3. Logistic model coefficients for self-reported sleep disturbance and polysomnography measured transitions to wake and S1

In Sections 3.2 and 4.1 the logistic regression models for the probability of a sleep stage transition to wake or S1 and the percent highly sleep disturbed calculated based on self-reported survey data were estimated using random effects logistic regression models which were calculated using the NLMIXED procedure in SAS. Generalized estimating equation (GEE) models were also calculated for the same outcomes using the GENMOD procedure in SAS. For the GEE models, an exchangeable working correlation matrix was used. The coefficients of the models are in Tables S9 through S14. The GEE models describe the average response of the participants. The random subject effects logistic regression models for the probability of transitions to wake or S1 describe the response of the average individual. The random study effects logistic regression models for the percent highly sleep disturbed describe the average study [11]. A more detailed discussion of the differences between the population average (PA, GEE model) and subject specific (SS, non-linear mixed model) approach can be found in Neuhaus et al. [12] and Schaffer et al. [13]. Point estimates for the logistic regressions and 95% confidence intervals were calculated for both types of models and the results are shown in Figure S1 and S2. The difference between the results obtained using the two modelling approaches was small and the confidence intervals strongly overlap. Therefore, although the interpretation of the results of the two types of models is different the strength of the effects reported within this evidence review is not significantly affected by the statistical model used.

Table S9. Model coefficients for the random study effect logistic regression model (Mixed) and the GEE model for the percent Highly Sleep Disturbed due to Aircraft noise.

Parameter	Mixed Model			GEE Model		
	**Coeff.**	**SE**	***p***	**Coeff.**	**SE**	***p***
Intercept	-4.7077	0.4401	0.0001	-4.4477	0.8028	<0.0001
L_{Night}	0.0661	0.0072	0.0003	0.0629	0.0132	<0.0001
Random Intercept (variance)	0.3426	0.2045				

Table S10. Model coefficients for the random study effect logistic regression model (Mixed) and the GEE model for the percent Highly Sleep Disturbed due to Road noise.

Parameter	Mixed Model			GEE Model		
	**Coeff.**	**SE**	***p***	**Coeff.**	**SE**	***p***
Intercept	-6.8968	0.4603	<0.0001	-6.2396	0.6993	<0.0001
L_{Night}	0.0754	0.0070	<0.0001	0.0666	0.0096	<0.0001
Random Intercept (variance)	0.5130					

Table S11. Model coefficients for the random study effect logistic regression model (Mixed) and the GEE model for the percent Highly Sleep Disturbed due to Train noise.

Parameter	Mixed Model			GEE Model		
	**Coeff.**	**SE**	***p***	**Coeff.**	**SE**	***p***
Intercept	-8.2977	0.5343	0.0001	-8.1181	1.0995	<0.0001
L_{Night}	0.1118	0.0091	0.0002	0.1092	0.0185	<0.0001
Random Intercept (variance)	0.1609	0.1099				

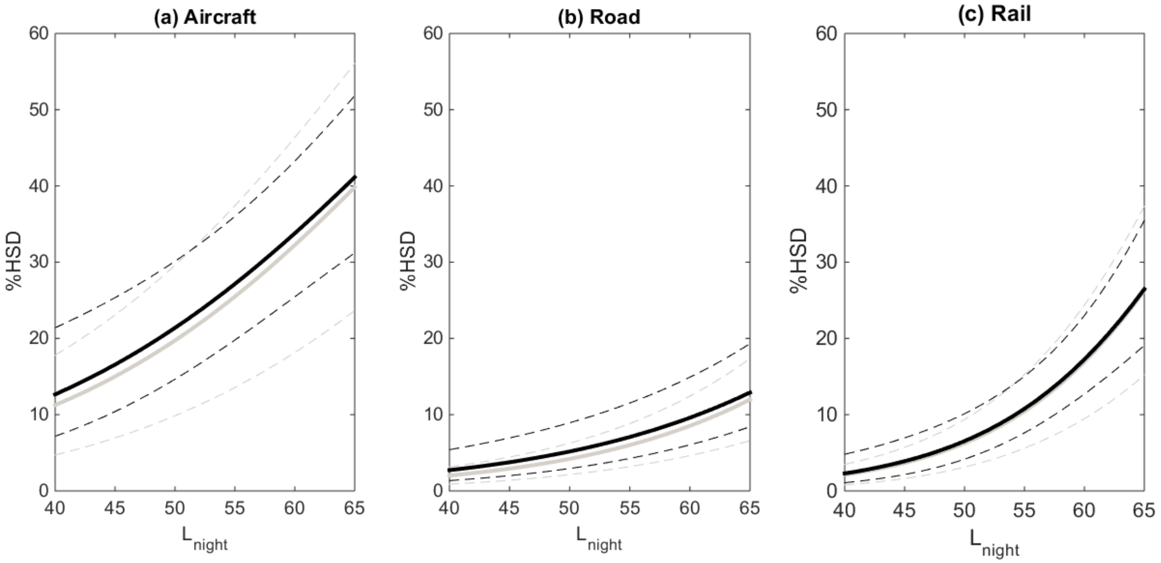

Figure S1. Percent Highly Sleep Disturbed. Random study effect logistic regression (gray) and GEE regression (black) with 95% confidence intervals (dashed lines).

Table S12. Model coefficients for the random subject effect logistic regression model (Mixed) and the GEE model for the probability of a sleep stage change to wake or S1 for Aircraft noise.

**Parameter**	**Mixed Model**			**GEE Model**		
	**Coeff.**	**SE**	***p***	**Coeff.**	**SE**	***p***
Intercept	-3.6052	0.2416	<0.0001	-3.4100	0.3484	<0.0001
$L_{AS,max}$	0.0301	0.0052	<0.0001	0.0269	0.0078	0.0006
Random Intercept (variance)	0.1603					

Table S13. Model coefficients for the random subject effect logistic regression model (Mixed) and the GEE model for the probability of a sleep stage change to wake or S1 for Road noise.

**Parameter**	**Mixed Model**			**GEE Model**		
	**Coeff.**	**SE**	***p***	**Coeff.**	**SE**	***p***
Intercept	-3.5495	0.2652	<0.0001	-3.4813	0.2463	<0.0001
$L_{AS,max}$	0.0307	0.0066	<0.0001	0.0307	0.0064	<0.0001
Random Intercept (variance)	0.1629					

Table S14. Model coefficients for the random subject effect logistic regression model (Mixed) and the GEE model for the probability of a sleep stage change to wake or S1 for Train noise.

**Parameter**	**Mixed Model**			**GEE Model**		
	**Coeff.**	**SE**	***p***	**Coeff.**	**SE**	***p***
Intercept	-3.7303	0.2744	<0.0001	-3.5741	0.2765	<0.0001
$L_{AS,max}$	0.0303	0.0055	<0.0001	0.0279	0.0057	<0.0001
Random Intercept (variance)	0.1056					

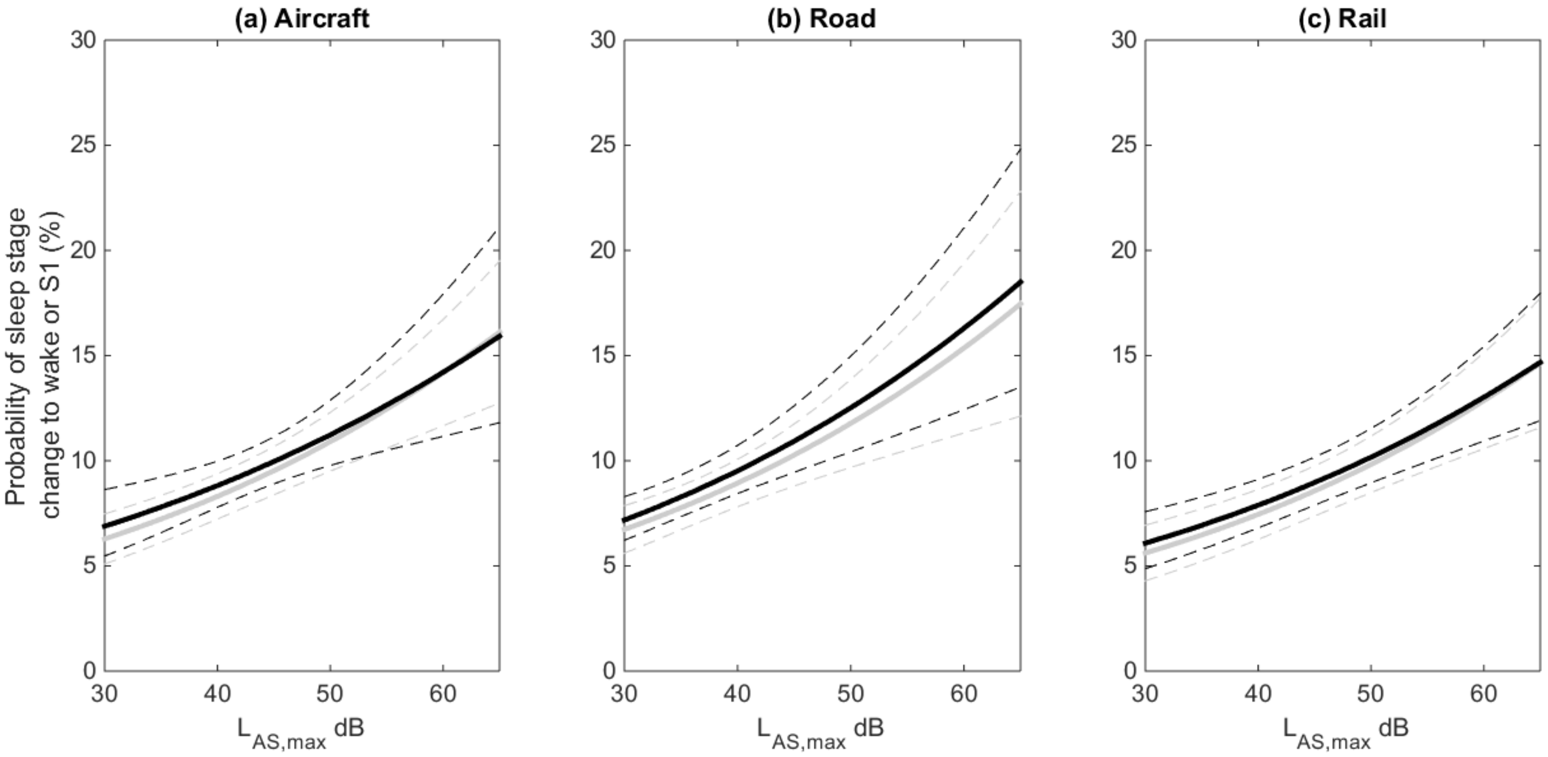

Figure S2. Probability of a sleep stage change to wake or S1. Random subject effect logistic regression (gray) and GEE regression (black) with 95% confidence intervals (dashed lines).

S4. Percent Highly Sleep Disturbed for 5 dB Intervals

Table S15. Percent Highly Sleep Disturbed for road, rail, and aircraft noise for the logistic regression models shown in Figure 8.

L_{Night}	AIR		ROAD		RAIL	
	Estimate [%]	95% CI [%]	Estimate [%]	95% CI [%]	Estimate [%]	95% CI [%]
40	11.26	4.72-17.81	2.02	0.90-3.15	2.13	0.79-3.48
45	15.01	6.95-23.08	2.92	1.40-4.44	3.67	1.63-5.71
50	19.73	9.87-29.60	4.21	2.14-6.27	6.25	3.12-9.37
55	25.49	13.57-37.41	6.02	3.19-8.84	10.43	5.61-15.26
60	32.25	18.15-46.36	8.54	4.64-12.43	16.92	9.48-24.37
65	39.85	23.65-56.05	11.98	6.59-17.36	26.27	15.20-37.33

S5. Assessment of the Risk of Bias of Individual Studies

Table S16. Criteria used to rate the bias of individual studies.

**Bias Due to Selection of Participants**	
Random sampling, Areas selected based on noise exposure, greater than 60% response rate, inclusion criteria not based on sleep and health criteria	Low
Response rate less than 60% or non-random sampling or sampling not based on noise exposure or individuals were excluded based on sleep and health criteria	High
Insufficient information to make a judgement	Unclear
**Bias Due to Noise Exposure Evaluation**	
For single event analysis: measured continuously in bedroom	Low
For long term noise level:<br>A. Based on measurements for at least 1week OR<br>B. Based on a noise map which was verified by noise measurements OR<br>C. Based on a noise map which was based on actual traffic data	Low
For long term noise level:<br>A. Based on measurements of less than 1 week or measurements were not continuous OR<br>B. Based on a noise map which was not verified by noise measurements or the predictions were not based on actual traffic data	High
Insufficient information to make a judgement	Unclear
**Bias Due to Sleep Measurement Outcome**	
Sleep questionnaire	High

Heart Rate or Blood Pressure	Low
Actigraphy	Low
Polysomnography	Low
Any other objective physiological measure	Low
Insufficient information to make a judgement	Unclear
**Bias Due to Confounding**	
All-important confounders taken into account in the analysis	Low
No adjustment for important confounders	High
Insufficient information to make a judgement	Unclear
**Overall Rating of Bias**	
All low ratings of bias	Low
1 or more high ratings of bias	High
All bias ratings of unclear or 1 or more unclear rating with all other ratings being low	Unclear

Table S17. Bias ratings for studies on noise from road, rail, and aircraft noise and cortical awakenings in adults.

Study	Bias Due to Participant Selection			Information Bias Due to Sleep Assessment Methodology		Information Bias Due to Exposure Assessment			Bias Due to Confounding Factors		Overall Bias Rating
	Response Rate	Inclusion/Exclusion Criteria	Bias Rating	Method	Bias Rating	Definition	Measurement Method	Bias Rating	Included in Analysis	Bias Rating	
Aasvang et al. (2011) [14]	25.6%	20-60 years old, good health, free of sleep disorders and cardiovascular disease.	High	PSG	Low	L_{Aeq}, night, L_{AFmax},night	Measured in bedroom	Low	Age	Low	High
Basner et al. (2006) [15]	Not specified	Free of existing sleep, chronic health, and mental illnesses.	High	PSG	Low	L_{Amax} indoors	Measured in bedroom	Low	Situational variables including elapsed sleep time and prior sleep stage.	Low	High
Elmenhorst et al. (2012) [16]	Not specified	Free of existing sleep, chronic health, and mental illnesses.	High	PSG	Low	L_{Amax} indoors	Measured in bedroom	Low	Age, gender, prior sleep stage, etc.	Low	High
Flindell et al. (2000) [17]	Approx. 5%	30-40 years old, noise sensitive, free of sleep and health disorders	High	PSG	Low	L_{Amax} indoors	Measured in bedroom	Low	Included noise condition, day of the week, and number of events.	Low	High

Table S18. Bias ratings for studies on road, rail, and aircraft noise and self-reported sleep disturbance.

Study	Bias Due to Participant Selection			Information Bias Due to Sleep Assessment Methodology		Information Bias Due to Exposure Assessment			Bias Due to Confounding Factors		Overall Bias Rating
	Response Rate	Inclusion/ Exclusion Criteria	Bias Rating	Method	Bias Rating	Definition	Measurement	Bias Rating	Included in Analysis	Bias Rating	
Nguyen et al. (2009) [18] -Ho chi Minh	88%	Adults 18 year or older were included.	Low	Question-naire	High	L_{night}, outdoors	Measured for 7 consecutive days	Low	Not in the reported analysis	High	High
Nguyen et al. (2010) , Nguyen et al. (2011) - Hanoi	91.6%	Adults 18 year or older were included.	Low	Question-naire	High	L_{night}, outdoors	Measured for 7 consecutive days.	Low	Not in the reported analysis	High	High
Nguyen et al. (2012) - Da Nang	84%	Sites were selected north and to the south of airport.	Low	Question-naire	High	L_{night}, outdoors	Measured for 7 consecutive days.	Low	Not in the reported analysis	High	High
Nguyen et al. (2015) - Hanoi	90%	Obtained responses at 11 survey sites and 2 control sites	Low	Question-naire	High	L_{night}, outdoors	Measured for 7 consecutive days.	Low	Not in the reported analysis	High	High
Phan et al. (2010) [19]- Hanoi	50%	In the two cities, 8 sites were selected based on traffic volume and residential and commercial characteristics.	High	Question-naire	High	L_{night}, outdoors	24-hour measurements were conducted at select sites.	High	Not in the reported analysis	High	High
Phan et al. (2010) [19]- Ho Chi Minh City	61%	In the two cities, 8 sites were selected based on traffic volume and residential and	Low	Question-naire	High	L_{night}, outdoors	24-hour measurements were conducted at select sites.	High	Not in the reported analysis	High	High

		commercial characteristics.									
Ristovska et al. (2009) [20]	72%	Sample was randomly selected from population living in Skopje. Inclusion criteria included age (18-65 years) and 1 year of residence at current living address.	Low	Question-naire	High	$L_{night,}$ outdoors	Performed short term measurements of 5 minutes in various locations within the city.	High	Adjusted for employment, educational level, residential period, time spent at home during working days and on the weekend.	Low	High
Sato et al. (2004) [21]	70.2% and 66.6%	Respondents were between 20-75 years old and were randomly selected from voter lists	Low	Question-naire	High	$L_{night,}$ outdoors	Measurements were made close to the railway. Then measurements were made at 5, 10, 20, and 40 m from the train line and equations for estimating the decay of the noise with distance was calculated and used to estimate the level at each house.	High	Not in the reported analysis	High	High
Bodin et al. (2015) [22]	54%	Participants were randomly sampled from 6 different noise strata	High	Question-naire	High	$L_{eq, 24 hr,}$ outdoors	Data in modelling included geometries of roads, buildings, elevation, ground types, noise barriers and railways.	Low	Adjusted for age, gender, BMI, smoking, marital status, education, hearing, and quiet side	Low	High
Brink et al. (2005) [23]	Unclear	Unclear	Unclea r	Question-naire	High	$L_{night,}$ outdoors	Unclear	Unclear	Unclear	Unclear	High
Brink (2011) [24]	Approx. 68%	Random selection of residents throughout Switzerland	Low	Question-naire	High	$L_{night,}$ outdoors	SonBase, noise levels at the most exposed façade.	High	Age, gender, BMI, socioeconomic	Low	High

									status, financial satisfaction		
Brown et al. (2015) [25]	75%	Random sample of individuals in Hong Kong. Individuals had to be 18 years or older to participate.	Low	Question-naire	High	$L_{night,}$ outdoors	Predicted for the most exposed façade, accounted for the height of the building	Low	Not in the reported analysis	High	High
Frei et al. (2014) [26]	31.4%	Questionnaire was sent to randomly selected residents from Basal who were between 30 and 60 years old. Participants were selected from a cohort on electromagnetic field exposure.	High	Epworth Sleepiness Scale and standardiz-ed questions from Swiss Health Survey	High	$L_{night,}$ outdoors	Modeled at the most exposed façade for the most exposed floor, reflections, absorptions, and noise protection walls are accounted for in the model.	Low	Models were adjusted for sex, age, education level, marital status, average daily physical activity, smoking status, average alcohol intake, body mass index, and a stress score.	Low	High
Halonen et al. (2012) [27]	Not specified	Participants were from the Finish Public Sector Study. The participants were selected among working employees in 10 towns and 6 hospital districts.	High	Question-naire	High	$L_{night,}$ outdoors	Noise levels were modeled for highways and main streets.	High	Adjusted for age, gender, occupational status, residence size, marital status, chronic disease, trait anxiety, and neighborhood socioeconomic disadvantage and population density.	Low	High
Hong et al. (2010) [28]	Approx. 65%	Convenience sample, recruited people that were going in and out of buildings within the sample regions.	High	Question-naire	High	$L_{night,}$ outdoors	3 nights of measurements at the most exposed façade of a building	High	Not in the reported analysis	High	High
Schreckenberg et al. (2009) [29]	61%	Random sample based on stratification of $L_{Aeq, 16h}$	Low	Question-naire-Including Pittsburgh Sleep	High	$L_{Aeq, 16 hr}$ and L_{night} outdoors	Noise levels were predicted	Low	Not in the reported analysis	High	High

				Quality Index							
Schreckenberg (2013) [30]	41%	Random sample	High	Question-naire	High	L_{night}, outdoors	Railway noise was predicted using the German railway noise model. The calculated noise levels were validated by comparing them to measured noise levels from a monitoring station.	Low	Not in the reported analysis	High	High
Shimoyama et al. (2014) [31]-Hanoi	50%	Not specified	High	Question-naire	High	L_{night}, outdoors	24-hour noise measurements were performed at survey locations.	High	Not in the reported analysis	High	High
Shimoyama et al. (2014) [31]- Ho Chi Minh	61%	Not specified	Low	Question-naire	High	L_{night}, outdoors	24-hour noise measurements were performed at survey locations.	High	Not in the reported analysis	High	High
Shimoyama et al. (2014) [31]- Da Nang	82%	Not specified	Low	Question-naire	High	L_{night}, outdoors	24-hour noise measurements were performed at survey locations.	High	Not in the reported analysis	High	High
Shimoyama et al. (2014) [31]- Hue	98%	Not specified	Low	Question-naire	High	L_{night}, outdoors	24-hour noise measurements were performed at survey locations.	High	Not in the reported analysis	High	High
Shimoyama et al. (2014) [31]- Thai Nguyen	81%	Not specified	Low	Question-naire	High	L_{night}, outdoors	24-hour noise measurements were performed at survey locations.	High	Not in the reported analysis	High	High
Yano et al. (2015) - Hanoi	68.5%	13 survey sites were selected based on their location relative to the runways.	Low	Question-naire	High	L_{night}, outdoors	Measured for 7 consecutive days.	Low	Not in the reported analysis	High	High

Table S19. Bias ratings for studies on wind turbine noise.

Study	Bias Due to Participant Selection			Information Bias Due to Sleep Assessment Methodology		Information Bias Due to Exposure Assessment			Bias Due to Confounding Factors		Overall Bias Rating
	Response Rate	Inclusion/Exclusion Criteria	Bias Rating	Method	Bias Rating	Definition	Measurement	Bias Rating	Included in Analysis	Bias Rating	
Aaron et al. (1996) [32]	NA-Volunteers	Inclusion criteria included being free of central nervous system and acute psychiatric illnesses.	High	PSG	Low	SPL	Measured in patient rooms	Low	None	High	High
Adachi et al. (2013) [33]	57.1% of eligible patients	Inclusion criteria included being 50 years and over, and having no cognitive impairment or pre-existing sleep disorders	Low	Survey-Karolinska Sleep Log,	High	L_{min}, L_{eq}, L_{max}	Measured in patient room and averaged over 1 hour intervals	Low	Age and Gender	Low	High
Elliott et al. (2013) [34]	8.7% of eligible patients	Exclusion criteria included sleep disorders, psychiatric illness, dementia or neurological impairment, and being in ICU for less than 24 hours	High	PSG	Low	L_{Aeq} and L_{Cpeak}	Measured in patient rooms	Low	None	High	High
Gabor et al. (2003) [35]	Not specified	Patients had to have endotracheal intubation and be mechanically ventilated for at least 24 hours. Healthy volunteers were excluded based on sleep disorders, medical history and history of being in ICU.	High	PSG	Low	SPL	Measured in patient rooms	Low	None	High	High
Freedman et al. (2013) [36]	Not specified	Exclusion criteria included receiving heavy sedation and having dementia	Unclear	PSG	Low	SPL	Measured in patient rooms	Low	Age, duration of ICU stay, and APACHE III	Low	Unclear
Hsu et al. (2010) [37]	Not specified	Inclusion criteria included that this was their first cardiac surgery, able to communicate verbally, pain under control, not using a respirator, no psychiatric illness or cognitive impairment	High	Questions on insomnia, heart rate and blood pressure measurements every 5 minutes.	High	SPL every second	Measured in patient room	Low	None	High	High
Missildine et al. (2010) [38]	Not specified, convenience sample	Inclusion criteria included aged >70 years, length of stay of 72 hours or longer. Patients were excluded for dementia, tremors or paralysis, poor vision or hearing, and sleep disorders.	High	Sleep Questionnaire and Actigraphy	Low	Median night time level from 11:00 pm to 7:00 am	Measured in patient rooms	Low	Age, mean lux	Low	High
Park et al. (2014) [39]	Not specified, 103 patients in 29 rooms	Exclusion criteria included hospitalization of less than 3 days, hearing problems, dementia, and psychiatric disorders.	High	Pittsburgh Sleep Quality Index	High	$L_{eq, 24 hr}$, and $L_{eq, day}$ (7am- 7 pm) and $L_{eq,night}$ (7pm-7am).	Measured in patient rooms	Low	Age, gender, severity of patient's disease, sleep medication use, and type of room	Low	High
Yoder et al. (2012) [40]	Of 145 eligible patients, 106 consented	Inclusion criteria included age 50 years and over, ambulatory, not cognitively impaired, no sleep disorders, and not transferred from ICU within 72 hours.	High	Pittsburgh Sleep Quality Index and Actigraphy	Low	L_{min}, L_{eq}, L_{max}	Measured in patient rooms	Low	Age, gender	Low	High

Table S20. Bias ratings for studies on hospital noise and sleep in adults.

Study	Bias Due to Participant Selection			Information Bias Due to Sleep Assessment Methodology		Information Bias Due to Exposure Assessment			Bias Due to Confounding Factors		Overall Bias Rating
	Response Rate	Inclusion/ Exclusion Criteria	Bias Rating	Method	Bias Rating	Definition	Measurement	Bias Rating	Included in Analysis	Bias Rating	
Pedersen and Persson Waye (2004) [41]	68.4%	5 areas were selected that represented a range of exposure to wind turbine noise. The participants had to be between the ages of 18 and 75.	Low	Questionnaire	High	SPL (A-weighted)	Calculated using the sound propagation model of the Swedish Environmental Protection Agency. Sound measurements were made to verify the predictions.	Low	Age, gender	Low	High
Pedersen and Persson Waye (2007) [42]	57.6%	7 areas were selected for the study. They were selected based on terrain and level of urbanization. Half of households with SPLs < 35 dBA were excluded to avoid high mailing cost.	High	Questionnaire	High	SPL (A-weighted)	Calculated using the sound propagation model of the Swedish Environmental Protection Agency.	Low	Age, gender	Low	High
Pedersen et al. (2009) [43]/Bakker (2012) [44]	37%	Representative sample of individuals exposed to wind turbine noise	High	Questionnaire	High	SPL (A-weighted)	Calculated sound propagation using a model similar to the ISO9613.2 sound propagation model.	Low	Age, gender, economic benefits	Low	High
Kuwano et. al. (2014) [45]	49% at the wind turbine sites and 45% at the control sites	The survey was conducted at 34 sites near wind turbines and at 16 control sites which were selected to have similar characteristics as the wind turbine sites.	High	Questionnaire	High	L_{Aeqn}	Measurements were completed at wind turbine sites for 5 consecutive days. Noise levels for individual respondents' houses were estimated from the results of the field measurements using a logarithmic regression.	High	Age, gender	Low	High
Michaud (2015) [46]	78.9% for the survey	All households within 600m of a wind turbine were selected. Households between 600 m and 10 km were randomly selected. Participants were between 18 and 79. Actigraphs were given to all interested individuals that completed the survey.	Low	Questionnaire and Actigraphy	Low	L_{den}	Predicted noise levels for each participant based on ISO standards and manufacturer provided A-weighted sound power levels.	Low	Variables included in the model were province, personal benefit, employment, audible rail noise, annoyed by snoring, migraines, dizziness, chronic pain, asthma, arthritis, diagnosed sleep.	Low	Low
Pawlaczyk-Luszcynsa et al. (2014) [47]	71%	Participants lived near 3 wind farms located in the central and north-western parts of Poland. The participants were age 15 to 82. There was no exclusion criteria applied.	Low	Questionnaire	High	L_{den}	Predicted noise levels for each participant was based on ISO standards and manufacturer provided A-weighted sound power levels. A correction factor of +4.7 dB was added to obtain L_{den} levels.	Low	None	High	High

Table S21. Bias ratings for studies on hospital noise and sleep in children.

Study	Bias Due to Participant Selection			Information Bias Due to Sleep Assessment Methodology		Information Bias Due to Exposure Assessment			Bias Due to Confounding Factors		Overall Bias Rating
	Response Rate	Inclusion/ Exclusion Criteria	Bias Rating	Method	Bias Rating	Definition	Measurement	Bias Rating	Included in Analysis	Bias Rating	
Corser (1996)[48]	Convenience sample	Inclusion criteria were children between 13 and 35 months, no neurological trauma, coma, seizures, and not receiving neuromuscular blocking agents.	High	Patient Sleep Behavior Observation Tool	High	SPL every 5 minutes	Not Specified	Unclear	None	High	High
Cureton-Lane and Fontaine (1997) [49]	Convenience sample	Inclusion criteria was children between 1 and 12 years old, in the PICU for ≥ 24 hours, not receiving neuromuscular blocking agents, no neurological dysfunction.	High	Patient Sleep Behavior Observation Tool	High	SPL every 5 minutes	Measured in patient rooms	Low	Noise, light, contact with caregivers, parental presence, and severity of illness.	Low	High
Kuhn et al. (2012), Kuhn et al. (2013) [50]	Convenience sample	The infants could not have severe brain injuries or received sedatives during the previous 48 hours.	High	Observational rating system for defining arousal states. Heart rate, respiratory rate, and SaO2.	Low	1 second L_{Aeq}	Placed near the blanket within the incubator	Low	None	High	High

Table S22. Bias ratings for studies on hospital noise studies that had interventions.

Study	Bias Due to Participant Selection			Information Bias Due to Sleep Assessment Methodology		Information Bias Due to Exposure Assessment			Bias Due to Confounding Factors		Overall Bias Rating
	**Response Rate**	**Inclusion/ Exclusion Criteria**	**Bias Rating**	**Method**	**Bias Rating**	**Definitio n**	**Measurement**	**Bias Rating**	**Included in Analysis**	**Bias Rating**	
Dennis et al. (2010) [51]	Convenience sample	Inclusion criteria included patients that were not sedated, and at least 18 years of age.	High	Observations of sleep	High	Average dBA	Noise levels were only recorded for 5 second time periods six times a day at the center of nurse's station, door of room and head of bed.	High	Each person observed before during and after quiet hours	Low	High
Duran et al. (2012) [52]	Convenience sample	Inclusion criteria included infants older than 7 days, weighing less than 1500 g, and in a closed incubator. Infants were excluded if they had congenital abnormalities, or unstable medical conditions.	High	Observed behavioral state and physiological measures including blood pressure, heart rate, respiration, body temperature, and oxygen saturation.	Low	Min, Max, and Mean values inside incubator	Inside and outside the incubator	Low	Each infant was observed with and without earmuffs	Low	High
Gardner et al. (2009) [53]	Convenience sample	Non-randomized sampling of patients from 2 hospitals.	High	Observed sleep state	High	SPL-daily	Measured in the patient rooms and the corridor	Low	None	High	High
Thomas et al. (2012) [54]	Convenience sample, all patients on the floor were screened daily for eligibility.	Eligible patients had to be medically stable, able to give verbal consent, and at least 16 years old.	High	Questionnaire	High	SPL	Measured in the patient rooms	Low	Compared across conditions, however not same subjects across conditions	High	High
Walder et al. (2000) [55]	Convenience sample	Different patients were enrolled pre and post intervention. Patients had a wide range of diagnosis and complications.	High	Nurses estimated the patient's sleep duration and the number of awakenings	High	SPL	Measured in the patient rooms	Low	Compared across conditions, however not same subjects across conditions	High	High

Table S23: Bias ratings for studies on noise from road, rail, and aircraft noise and cardiac and blood pressure outcomes.

Study	Bias Due to Participant Selection			Information Bias Due to Sleep Assessment Methodology		Information Bias Due to Exposure Assessment			Bias Due to Confounding Factors		Overall Bias Rating
	Response Rate	Inclusion/Exclusion Criteria	Bias Rating	Method	Bias Rating	Definition	Measurement	Bias Rating	Included in Analysis	Bias Rating	
Haralabidis et al. (2008) [56]	Approx. 30% in Italy and the UK, 56% in Greece and 78% in Sweden	Exclusion criteria included taking antihypertensive medication, diabetes, sleep apnea syndrome, and diagnosis of hearing impairment.	High	HR and Blood Pressure	Low	L_{Amax} indoor	Measured in bedroom	Low	No adjustment but calculated models with random subject intercept and with random coefficients	Low	High
Graham et al. (2009) [57]	Approx. 7%	Exclusion criteria was having cardiovascular disease	High	Respiratory sinus arrhythmia and pre-ejection period	Low	L_{Spt} indoor	Measured in bedroom	Low	Examined a range of variables including gender, age, BMI, education, resident years, medication, source, caffeine, alcohol, and cigarettes use.	Low	High

Table S24. Bias ratings for studies on noise from road, rail, and aircraft noise and actigraphy measured outcomes.

Study	Bias Due to Participant Selection			Information Bias Due to Sleep Assessment Methodology		Information Bias Due to Exposure Assessment			Bias Due to Confounding Factors		Overall Bias Rating
	Response Rate	Inclusion/Exclusion Criteria	Bias Rating	Method	Bias Rating	Definition	Measurement	Bias Rating	Included in Analysis	Bias Rating	
Hong et al. (2006) [58]	Not specified	Not specified	Unclear	Actigraphy	Low	L_{Amax} indoor	Indoor levels were measured.	Low	None	High	High
Frei et al. (2014) [26]	NA	Selection was based on RF-EMF sources. Exclusion criteria included young children and recent long-distance flights.	High	Actigraphy	Low	L_{night} outdoors	Modeled at the most exposed façade for the most exposed floor	Low	Adjusted for many variables including presence of bed partner, window closing habits, age, gender, BMI	Low	High
Griefahn et al. (2000) [6]	Not specified	Selected equally across noise exposure and gender from those that completed a survey. Exclusion criteria included having a chronic illness that impaired sleep, and hearing loss.	Unclear	Actigraphy	Low	L_{eq}	Levels were measured each night at the dominant noise source (rail track or road), during one night in the bedroom and outdoors in front of bedroom window.	Low	Not specified	Unclear	Unclear
Lercher et al. (2010) [59]	Not specified	Eight volunteers who agreed to installation of equipment	High	Seismo-somnography	Low	L_{Amax} indoor	Measured in bedroom at half-open window.	Low	Adjusted for variables including rise time, duration of event and time from sleep onset	Low	High
Ohrstrom et al. (2006) [60]	Not specified	Stratified sample based on $L_{Aeq,\ 24\ hour}$ noise levels. Could not work night shifts.	Unclear	Questionnaire and Actigraphy	Low	$L_{Aeq,\ 24h}$ outdoors	Modeled at the most exposed façade	Low	None	High	High
Passchier-Vermeer et al. (2002) [61]	18%	Exclusion criteria included taking care of family members at night, and taking sleeping pills.	High	Actigraphy	Low	L_{Amax} indoor	Measured in bedroom	Low	Not for the individual event models	High	High
Passchier-Vermeer et al. (2007) [62]	7%	Exclusion criteria included taking care of family members at night, taking sleeping pills	High	Actigraphy	Low	L_{Amax} indoor	Measured in bedroom	Low	Not for the individual event models	High	High
Pirrera et al. (2014) [63]	Less than 4% for mailed letters	Selected based on quiet/noisy area. Inclusion criteria included regular sleep schedule, no young children, and duration of residence of >1 year.	High	Actigraphy	Low	L_{Aeq}, L_{Amax} (TIB) indoor	Measured in bedroom	Low	None	High	High

Table S25. Bias ratings for studies on noise from road, rail, and aircraft noise and children's sleep.

Study	Bias Due to Participant Selection			Information Bias Due to Sleep Assessment Methodology		Information Bias Due to Exposure Assessment			Bias Due to Confounding Factors		Overall Bias Rating
	Response Rate	Inclusion/Exclusion Criteria	Bias Rating	Method	Bias Rating	Definition	Measurement	Bias Rating	Included in Analysis	Bias Rating	
Ising and Ising (2002) [64]	NA	Participants of a specific village were asked to a meeting on noise induced health effects	High	Questionnaire	High	L_{Cmax} indoors	Measured in bedroom	Low	Age, gender, social status	Low	High
Lercher et al. (2013) [65]	85.5%	3rd and 4th graders from 49 schools	Low	Questionnaire	High	L_{den} outdoors	Modeled at the most exposed façade	Low	Gender, health status, and mother's education	Low	High
Ohrström et al. (2006) [60]	Not specified	Stratified sample based on $L_{Aeq,\ 24\ hour}$ noise levels. Children had to have normal hearing.	Low	Questionnaire and Actigraphy	Low	$L_{Aeq,\ 24h}$ outdoors	Modeled at the most exposed façade	Low	None	High	High
Tiesler et al. (2013) [66]	NA	Data from ongoing population based birth-cohort studies. Inclusion criteria was participation in a 10 year follow-up, availability of noise exposure data, and information available on behavioral problems	High	Questionnaire	High	L_{night} outdoors	Modeled at the most and least exposed façade	Low	Gender, age, parental education level, mother's age at birth, television/computer usage, single parent status, sleeping alone, and orientation of the window	Low	High

Table S26. Bias ratings for studies that were not included in the meta-analysis of self-reported sleep outcomes for road, rail, and aircraft noise.

Study	Bias Due to Participant Selection			Information Bias Due to Sleep Assessment Methodology		Information Bias Due to Exposure Assessment			Bias Due to Confounding Factors		Overall Bias Rating
	Response Rate	Inclusion/ Exclusion Criteria	Bias Rating	Method	Bias Rating	Definition	Measurement	Bias Rating	Included in Analysis	Bias Rating	
Aasvang et al. (2008) [1]	63.7%	Sample was age and gender stratified.	Low	Question-naire	High	L_{night} bedroom façade	Predicted noise levels included sound propagation effects such as distance from receiver to railway line, air absorption, ground properties, etc.	Low	Included covariates including age, gender, household income, education, noise sensitivity, type of bedroom window, duration of residence, and number of trains	Low	High
Bluhm et al. (2004) [2]	76%	19-80 years of age	Low	Question-naire	High	$L_{eq,\ 24\ hour}$ outdoors	Predicted noise levels, unclear on methods used to predict levels	Un-clear	Not in the reported analysis	High	High
Bristow and Wardman (2003) [3]	73%	No exclusion criteria	Low	Question-naire	High	L_{night} outdoors	Predicted noise levels	Low	Models with various quality of life parameters	Low	High
Wardman et al. (2012) [4]	Unclear	Unclear	Unclear	Question-naire	High	L_{night} outdoors	Unclear	Un-clear	Unclear	Un-clear	High
Fyhri and Aasvang (2010) [5]	60.5%	No exclusion criteria	Low	Question-naire	High	$L_{night,}$ outdoors	Noise levels were calculated at the bedroom façade	Low	Included age, gender, noise sensitivity, annoyance, education	Low	High
Griefahn et al. (2000) [6]	Not Specified	18-70 years, residential time of at least 12 months, no chronic diseases usually accompanied with sleep disturbance, no regular intake of remedies which influence sleep, no significant hearing loss, no shift work	High	Question-naire	High	$L_{night,}$ outdoors	Predicted noise levels, method for prediction not described	Un-clear	Not reported	Un-clear	High

Jakoljevic et al. (2006) [7]	77%	Inclusion criteria included living at the present address for more than 10 years, bedroom window had to face the street.	Low	Question-naire	High	L_{eq} outdoors	Measurements were made at 2 sites for each of 6 streets. The measurements were made for 15-minute periods at several times of day.	High	Adjusted for age, sex, noise sensitivity, neuroticism, and extroversion.	Low	High
Ohrstrom, Skanberg et al. (2006) [8]	59%	Study sites were selected to have noise levels between 45 -65. Sites were selected to have specific levels at the most and least exposed façade.	High	Question-naire	High	$L_{night,}$ outdoors	Predicted noise levels based on traffic. 1 week long-term measurements and 30 minute short term measurements were made at representative locations.	Low	None	High	High
Ohrstrom et al. (2010) [9]	49%	Two study sites were selected in areas with railway traffic and 2 sites were selected in areas with road traffic noise	High	Questionnair e	High	$L_{night,}$ outdoors	Predicted for the most exposed façade	High	Examined windows open versus closed and whether bedroom window was facing towards the road or railway line.	High	High
Stosic et al. (2009) [10]	35.4%	Distributed questionnaires to residents of 3 busy streets and 3 quiet side streets. Inclusion criteria included living at current address for over a year, bedroom windows had to face the street. Individuals were excluded if they had chronic diseases that might cause sleep disturbance and hearing loss.	High	Question-naire	High	$L_{night,}$ outdoors	L_{eq} levels were measured at 6 sites.	High	Not in the reported analysis	High	High

S6. Literature Review Search Terms

(((TITLE-ABS-KEY(environmental* AND noise*) OR TITLE-ABS-KEY(communit* AND noise*) OR TITLE-ABS-KEY(traffic* AND noise*) OR TITLE-ABS-KEY(wind* AND turbine* AND noise*) OR TITLE-ABS-KEY(wind* AND turbine* AND sound*) OR TITLE-ABS-KEY(wind* AND farm* AND sound*) OR TITLE-ABS-KEY(wind* AND farm* AND noise*) OR TITLE-ABS-KEY(airport* AND noise*) OR TITLE-ABS-KEY(aircraft* AND noise*) OR TITLE-ABS-KEY(railway* AND noise*) OR TITLE-ABS-KEY(road* AND traffic* AND noise*) OR TITLE-ABS-KEY(transportation* AND noise*) OR TITLE-ABS-KEY(train* AND noise*) OR TITLE-ABS-KEY(leisure* AND noise*) OR TITLE-ABS-KEY(neighbourhood* AND noise*) OR TITLE-ABS-KEY(neighborhood* AND noise*) OR TITLE-ABS-KEY(household* AND noise*) OR TITLE-ABS-KEY(low* AND frequency* AND noise*) OR TITLE-ABS-KEY(classroom* AND noise*) OR TITLE-ABS-KEY(school* AND noise*) OR TITLE-ABS-KEY(high* AND volume* AND music*) OR TITLE-ABS-KEY(high* AND volume* AND noise*) OR TITLE-ABS-KEY(personal* AND electronic* AND device* AND noise*) OR TITLE-ABS-KEY(mp3* AND player* AND noise*) OR TITLE-ABS-KEY(toy* AND noise*) OR TITLE-ABS-KEY(hospital* AND noise*) OR TITLE-ABS-KEY(combined* AND exposure* AND noise*) OR TITLE-ABS-KEY(nuisance* AND noise*) OR TITLE-ABS-KEY(expos* AND noise*) OR TITLE-ABS-KEY(truck* AND noise*) OR TITLE-ABS-KEY(motor* AND vehicle* AND noise*) OR TITLE-ABS-KEY(motorcycle* AND noise*) OR TITLE-ABS-KEY(social* AND noise*) OR TITLE-ABS-KEY(load* AND noise*)) OR (TITLE-ABS-KEY(entertainment AND noise*) OR TITLE-ABS-KEY(noise AND mobile AND phone*) OR TITLE-ABS-KEY(noise AND audio AND device*) OR TITLE-ABS-KEY(noise AND music* AND player*) OR TITLE-ABS-KEY(combin* AND expos* AND noise*) OR TITLE-ABS-KEY(combin* AND expos* AND air* AND pollution*))))
AND
((TITLE-ABS-KEY(insomnia*) OR TITLE-ABS-KEY(sleep*) OR TITLE-ABS-KEY(sub-cortical* AND arous*) OR TITLE-ABS-KEY(autonomic* AND arous*) OR TITLE-ABS-KEY(awaken*) OR TITLE-ABS-KEY(waking) OR TITLE-ABS-KEY(wake*) OR TITLE-ABS-KEY(day* AND cognit* AND performanc*) OR TITLE-ABS-KEY(tired*) OR TITLE-ABS-KEY(fatig*) OR TITLE-ABS-KEY(perceiv* AND wellbeing*) OR TITLE-ABS-KEY(mood* AND change*) OR TITLE-ABS-KEY(injur*)))

S7. Studies Excluded from the Qualitative and Quantitative Review

Studies excluded from the qualitative and quantitative review studies are listed as references 67-115 below.

References

1. Aasvang, G.M.; Moum, T.; Engdahl, B. Self-reported sleep disturbances due to railway noise: Exposure-response relationships for nighttime equivalent and maximum noise levels. *J. Acoust. Soc. Am.* **2008**, *124*, 257–268.
2. Bluhm, G.; Nordling, E.; Berglind, N. Road traffic noise and annoyance—An increasing environmental health problem. *Noise Health* **2004**, *6*, 43–49.
3. Bristow, A.; Wardman, M. Attitudes Towards and Values of Aircraft Annoyance and Noise Nuisance. Attitudes to Aircraft Annoyance around Airports (5a) Survey Report. EEC/SEE/2003/002. EUROCONTROL Experimental Centre, France. 2003. Available online: http://www.sea-acustica.es/fileadmin/publicaciones/Guimaraes04_ID116.pdf (accessed on 7 March 2018).
4. Wardman, M.; Bristow, A.; Tight, M.; Guehnemann, A.; Shires, J. Inter-temporal variations in the valuation of aircraft noise nuisance. In Proceedings of the Transportation Research Board 91st Annual Meeting, Washington, DC, USA, 22–26 January 2012.
5. Fyhri, A.; Aasvang, G.M. Noise, sleep and poor health: Modeling the relationship between road traffic noise and cardiovascular problems. *Sci. Total Environ.* **2010**, *408*, 4935–4942.
6. Griefahn, B.; Schuemer-Kohrs, A.; Schuemer, R.; Mohler, U.; Mehnert, P. Physiological, subjective, and behavioural responses to noise from rail and road traffic. *Noise & Health* **2000**, *3*, 59-71.
7. Jakovljević, B.; Belojević, G.; Paunović, K.; Stojanov, V. Road traffic noise and sleep disturbances in an urban population: Cross-sectional study. *Croat. Med. J.* **2006**, *47*, 125–133.
8. Ohrstrom, E.; Skanberg, A.; Svensson, H.; Gidlof-Gunnarsson, A. Effects of road traffic noise and the benefit of access to quietness. *J. Sound Vib.* **2006**, *295*, 40–59.
9. Öhrström, E.; Gidlöf-Gunnarsson, A.; Ögren, M.; Jerson, T. In *Comparative field studies on the effects of railway and road traffic noise*, 39th International Congress and Exposition on Noise Control Engineering, InterNoise Lisbon, Portugal, June 15-16, **2010**; Lisbon, Portugal, pp 526-534
10. Stošić, L.; Belojević, G.; Milutinović, S. Effects of traffic noise on sleep in an urban population. *Arh. Hig. Rada Toksikol.* **2009**, *60*, 335–342.
11. Hu, F.B.; Goldberg, J.; Hedeker, D.; Flay, B.R.; Pentz, M.A. Comparison of population-averaged and subject-specific approaches for analyzing repeated binary outcomes. *Am. J. Epidemiol.* **1998**, *147*, 694–703.
12. Neuhaus, J.M., Kalbfleisch, J.D., Hauck, W.W. A comparison of cluster-specific and population average approaches for analyzing correlated binary data. *Int Stat Rev* **1991**, 59, 25-35.
13. Schaffer, B., Pieren, R., Mendolia, F., Basner, M., Brink, M. Noise exposure-response relationships established from repeated binary observations: Modeling approaches and applications. *J Acoust Soc Am* **2017**, 141, 3175-85.
14. Aasvang, G.M.; Overland, B.; Ursin, R.; Moum, T. A field study of effects of road traffic and railway noise on polysomnographic sleep parameters. *J. Acoust. Soc. Am.* **2011**, 129, 3716–3726.
15. Basner, M.; Isermann, U.; Samel, A. Aircraft noise effects on sleep: Application of the results of a large polysomnographic field study. J. Acoust. Soc. Am. **2006**, 119, 2772–2784.
16. Elmenhorst, E.M.; Pennig, S.; Rolny, V.; Quehl, J.; Mueller, U.; Maass, H.; Basner, M. Examining nocturnal railway noise and aircraft noise in the field: Sleep, psychomotor performance, and annoyance. *Sci. Total Environ.* **2012**, 424, 48–56.
17. Flindell, I.H.; Bullmore, A.J.; Robertson, K.A.; Wright, N.A.; Turner, C.; Birch, C.L.; Jiggins, M.; Berry, B.F.; Davison, M.; Dix, N. Aircraft Noise and Sleep, 1999 UK Trial Methodology Study; ISVR Consultancy Services; Institute of Sound and Vibration Research, University of Southampton: Southampton, UK, 2000.
18. Nguyen, T.L.; Yano, T.; Nishimura, T.; Sato, T. Social survey on community response to aircraft noise in Ho Chi Minh city. In Proceedings of the 38th International Congress and Exposition on Noise Control Engineering, InterNoise, Ottawa, ON, Canada, 23–26 August 2009; pp. 1146–1154.
19. Phan, H.Y.T.; Yano, T.; Phan, H.A.T.; Nishimura, T.; Sato, T.; Hashimoto, Y. Community responses to road traffic noise in Hanoi and Ho Chi Minh City. *Appl. Acoust.* **2010**, 71, 107–114.

20. Ristovska, G.; Gjorgjev, D.; Stikova, E.; Petrova, V.; Cakar, M.D. Noise induced sleep disturbance in adult population: Cross sectional study in Skopje urban centre. *Maced. J. Med. Sci.* **2009**, 2, 255–260.
21. Sato, T.; Yano, T.; Morihara, T.; Masden, K. Relationships between rating scales, question stem wording, and community responses to railway noise. *J. Sound Vib.* **2004**, 277, 609–616.
22. Bodin, T.; Björk, J.; Ardö, J.; Albin, M. Annoyance, sleep and concentration problems due to combined traffic noise and the benefit of quiet side. *Int. J. Environ. Res. Public Health* **2015**, 12, 1612–1628.
23. Brink, M.; Wirth, K.; Rometsch, R.; Schierz, C. *Lärmstudie 2000 Zusammenfassung. ETH Zürich, Zentrum für Organisations- und Arbeitswissenschaften*; Switzerland, E.Z., Ed.; Institute for Organizational and Occupational Sciences (ZOA): Zurich, Seitzerland, 2005.
24. Brink, M. Parameters of well-being and subjective health and their relationship with residential traffic noise exposure—A representative evaluation in Switzerland. *Environ. Int.* **2011**, 37, 723–733.
25. Brown, A.L.; Lam, K.C.; van Kamp, I. Quantification of the exposure and effects of road traffic noise in a dense Asian city: A comparison with western cities. *Environ. Health* **2015**, 14, 22.
26. Frei, P.; Mohler, E.; Röösli, M. Effect of nocturnal road traffic noise exposure and annoyance on objective and subjective sleep quality. Int. J. Hyg. *Environ. Health* **2014**, 217, 188–195.
27. Halonen, J.I.; Vahtera, J.; Stansfeld, S.; Yli-Tuomi, T.; Salo, P.; Pentti, J.; Kivimaki, M.; Lanki, T. Associations between Nighttime Traffic Noise and Sleep: The Finnish Public Sector Study. *Environ. Health Perspect*. **2012**, 120, 1391–1396.
28. Hong, J.; Kim, J.; Lim, C.; Kim, K.; Lee, S. The effects of long-term exposure to railway and road traffic noise on subjective sleep disturbance. *J. Acoust. Soc. Am.* **2010**, 128, 2829–2835.
29. Schreckenberg, D.; Heudorf, U.; Eikmann, T.; Meis, M. Aircraft noise and health of residents living in the vicinity of Frankfurt airport. In Proceedings of the Euronoise, Edinburgh, Scotland, 26–28 October 2009.
30. Schreckenberg, D. Exposure-response relationship for railway noise annoyance in the Middle Rhine Valley. In Proceedings of the 42th Interational Congress and Exposition on Noise Control Engineering, InterNoise, Innsbruck, Austria, 15–18 September 2013; pp. 4997–5006.
31. Shimoyama, K.; Nguyen, T.L.; Yano, T.; Morihara, T. Social surveys on community response to road traffic in five cities in Vietnam. In Proceedings of the 43th International Congress and Exposition on Noise Control Engineering, InterNoise, Melbourne, Australia, 16–19 November 2014; pp. 815–822.
32. Aaron, J.N.; Carlisle, C.C.; Carskadon, M.A.; Meyer, T.J.; Hill, N.S.; Millman, R.P. Environmental noise as a cause of sleep disruption in an intermediate respiratory care unit. *Sleep* **1996**, 19, 707–710.
33. Adachi, M.; Staisiunas, P.G.; Knutson, K.L.; Beveridge, C.; Meltzer, D.O.; Arora, V.M. Perceived control and sleep in hospitalized older adults: A sound hypothesis? *J. Hosp. Med.* **2013**, 8, 184–190.
34. Elliott, R.; McKinley, S.; Cistulli, P.; Fien, M. Characterisation of sleep in intensive care using 24-hour polysomnography: An observational study. *Crit. Care* **2013**, 17, R46.
35. Gabor, J.Y.; Cooper, A.B.; Crombach, S.A.; Lee, B.; Kadikar, N.; Bettger, H.E.; Hanly, P.J. Contribution of the intensive care unit environment to sleep disruption in mechanically ventilated patients and healthy subjects. Am. J. Respir. *Crit. Care Med.* **2003**, 167, 708–715.
36. Freedman, N.S.; Gazendam, J.; Levan, L.; Pack, A.I.; Schwab, R.J. Abnormal sleep/wake cycles and the effect of environmental noise on sleep disruption in the intensive cave unit. *Am. J. Respir. Crit. Care Med.* **2001**, 163, 451–457.
37. Hsu, S.M.; Ko, W.J.; Liao, W.C.; Huang, S.J.; Chen, R.J.; Li, C.Y.; Hwang, S.L. Associations of exposure to noise with physiological and psychological outcomes among post-cardiac surgery patients in ICUs. *Clinics* **2010**, 65, 985–989.
38. Missildine, K.; Bergstrom, N.; Meininger, J.; Richards, K.; Foreman, M.D. Sleep in Hospitalized Elders: A Pilot Study. *Geriatr. Nurs.* **2010**, 31, 263–271.
39. Park, M.J.; Yoo, J.H.; Cho, B.W.; Kim, K.T.; Jeong, W.C.; Ha, M. Noise in hospital rooms and sleep disturbance in hospitalized medical patients. *Environ. Health Toxicol.* **2014**, 29, e2014006.
40. Yoder, J.C.; Staisiunas, P.G.; Meltzer, D.O.; Knutson, K.L.; Arora, V.M. Noise and sleep among adult medical inpatients: Far from a quiet night. *Arch. Intern. Med.* **2012**, 172, 68–70.

41. Pedersen, E.; Persson Waye, K. Perception and annoyance due to wind turbine noise—A dose-response relationship. *J. Acoust. Soc. Am.* **2004**, 116, 3460–3470.
42. Pedersen, E.; Persson Waye, K. Wind turbine noise, annoyance and self-reported health and well-being in different living environments. *Occup. Environ. Med.* **2007**, 64, 480–486.
43. Pedersen, E.; van den Berg, F.; Bakker, R.; Bouma, J. Response to noise from modern wind farms in The Netherlands. *J. Acoust. Soc. Am.* **2009**, 126, 634–643.
44. Bakker, R.H.; Pedersen, E.; van den Berg, G.P.; Stewart, R.E.; Lok, W.; Bouma, J. Impact of wind turbine sound on annoyance, self-reported sleep disturbance and psychological distress. *Sci. Total Environ.* **2012**, 425, 42–51.
45. Kuwano, S.; Yano, T.; Kageyama, T.; Sueoka, S.; Tachibana, H. Social survey on wind turbine noise in Japan. *Noise Control Eng. J.* **2014**, 62, 503–520.
46. Michaud, D.S. Self-reported and objectively measured outcomes assessed in the Health Canada Wind Turbine Noise and Health Sttudy: Results support an increase in community annoyance. In Proceedings of the 44nd International Congress and Exposition on Noise Control Engineering, InterNoise, San Francisco, CA, USA, 9–12 August 2015.
47. Pawlaczyk-Luszczynska, M.; Dudarewicz, A.; Zaborowski, K.; Zamojska-Daniszewska, M.; Waszkowska, M. Evaluation of annoyance from the wind turbine noise: A pilot study. *Int. J. Occup. Med. Environ. Health* **2014**, 27, 364–388.
48. Corser, N.C. Sleep of 1- and 2-year-old children in intensive care. *Issues Compr. Pediatr. Nurs.* **1996**, 19, 17–31.
49. Cureton-Lane, R.A.; Fontaine, D.K. Sleep in the pediatric ICU: An empirical investigation. *Am. J. Crit. Care* **1997**, 6, 56–63.
50. Kuhn, P.; Zores, C.; Langlet, C.; Escande, B.; Astruc, D.; Dufour, A. Moderate acoustic changes can disrupt the sleep of very preterm infants in their incubators. *Acta Paediatr.* **2013**, 102, 949–954.
51. Dennis, C.M.; Lee, R.; Woodard, E.K.; Szalaj, J.J.; Walker, C.A. Benefits of quiet time for neuro-intensive care patients. *J. Neurosci. Nurs.* **2010**, 42, 217–224.
52. Duran, R.; Ciftdemir, N.A.; Ozbek, U.V.; Berberoglu, U.; Durankus, F.; Sut, N.; Acunas, B. The effects of noise reduction by earmuffs on the physiologic and behavioral responses in very low birth weight preterm infants. *Int. J. Pediatr. Otorhinolaryngol.* **2012**, 76, 1490–1493.
53. Gardner, G.; Collins, C.; Osborne, S.; Henderson, A.; Eastwood, M. Creating a therapeutic environment: A non-randomised controlled trial of a quiet time intervention for patients in acute care. *Int. J. Nurs. Stud.* **2009**, 46, 778–786.
54. Thomas, K.P.; Salas, R.E.; Gamaldo, C.; Chik, Y.; Huffman, L.; Rasquinha, R.; Hoesch, R.E. Sleep rounds: A multidisciplinary approach to optimize sleep quality and satisfaction in hospitalized patients. *J. Hosp. Med.* **2012**, 7, 508–512.
55. Walder, B.; Francioli, D.; Meyer, J.J.; Lancon, M.; Romand, J.A. Effects of guidelines implementation in a surgical intensive care unit to control nighttime light and noise levels. *Crit. Care Med.* **2000**, 28, 2242–2247.
56. Haralabidis, A.S.; Dimakopoulou, K.; Vigna-Taglianti, F.; Giampaolo, M.; Borgini, A.; Dudley, M.L.; Pershagen, G.; Bluhm, G.; Houthuijs, D.; Babisch, W.; et al. Acute effects of night-time noise exposure on blood pressure in populations living near airports. *Eur. Heart J.* **2008**, 29, 658–664.
57. Graham, J.M.A.; Janssen, S.A.; Vos, H.; Miedema, H.M.E. Habitual traffic noise at home reduces cardiac parasympathetic tone during sleep. *Int. J. Psychophysiol.* **2009**, 72, 179–186.
58. Hong, J.; Lim, C.; Kim, J.; Lee, S. Assessment of sleep disturbance on night-time railway noise from the field survey. In Proceedings of the 35th International Congress and Exposition on Noise Control Engineering, InterNoise, Honolulu, HI, USA, 3–6 December 2006; pp. 4648–4656.
59. Lercher, P.; Brink, M.; Rudisser, J.; Van Renterghem, T.; Botteldooren, D.; Baulac, M.; Defrance, J. The effec ts of railway noise on sleep medication intake: Results from the ALPNAP-study. *Noise Health* **2010**, 12, 110–119.
60. Öhrström, E.; Hadzibajramovic, E.; Holmes, M.; Svensson, H. Effects of road traffic noise on sleep: Studies on children and adults. *J. Environ. Psychol.* **2006**, 26, 116–126.

61. Passchier-Vermeer, W.; Vos, H.; Steenbekkers, J.H.M.; Van der Ploeg, F.D.; Groothuis-Oudshoorn, K. Sleep Disturbance and Aircraft Noise Exposure-Exposure Effect Relationships; TNO: The Hague, The Netherlands, 2002.
62. Passchier-Vermeer, W.; Vos, H.; Janssen, S.A.; Miedema, H.M. Sleep and Traffic Noise, Summary Report; TNO: Delft, The Netherlands, 2007.
63. Pirrera, S.; De Valck, E.; Cluydts, R. Field study on the impact of nocturnal road traffic noise on sleep: The importance of in- and outdoor noise assessment, the bedroom location and nighttime noise disturbances. *Sci. Total Environ.* **2014**, 500–501, 84–90.
64. Ising, H.; Ising, M. Chronic Cortisol Increases in the First Half of the Night Caused by Road Traffic Noise. *Noise Health* **2002**, 4, 13–21.
65. Lercher, P.; Eisenmann, A.; Dekonick, L.; Botteldooren, D. The relation between disturbed sleep in children and traffic noise exposure in alpine valleys. In Proceedings of the 42nd International Congress and Exposition on Noise Control Engineering, InterNoise, Innsbruck, Austria, 15–18 September 2013.
66. Tiesler, C.M.T.; Birk, M.; Thiering, E.; Kohlboeck, G.; Koletzko, S.; Bauer, C.-P.; Berdel, D.; von Berg, A.; Babisch, W.; Heinrich, J.; et al. Exposure to road traffic noise and children's behavioural problems and sleep disturbance: Results from the GINIplus and LISAplus studies. *Environ. Res.* **2013**, 123, 1–8.
67. Aasvang, G.M.; Moum, T.; Engdahl, B. Self-reported sleep disturbances due to railway noise: Exposure-response relationships for nighttime equivalent and maximum noise levels. *J. Acoust. Soc. Am.* **2008**, *124*, 257–268.
68. Bluhm, G.; Nordling, E.; Berglind, N. Road traffic noise and annoyance—An increasing environmental health problem. *Noise Health* **2004**, *6*, 43–49.
69. Bristow, A.; Wardman, M. Attitudes Towards and Values of Aircraft Annoyance and Noise Nuisance. Attitudes to Aircraft Annoyance around Airports (5a) Survey Report. EEC/SEE/2003/002. EUROCONTROL Experimental Centre, France. 2003. Available online: https://www.eurocontrol.int/eec/gallery/content/public/document/eec/report/2003/012a.5A_Aircraft_Annoyance_and_Noise_Nuisance.pdf (accessed on 13 March 2018).
70. Wardman, M.; Bristow, A.; Tight, M.; Guehnemann, A.; Shires, J. Inter-temporal variations in the valuation of aircraft noise nuisance. In Proceedings of the Transportation Research Board 91st Annual Meeting, Washington, DC, USA, 22–26 January 2012.
71. Fyhri, A.; Aasvang, G.M. Noise, sleep and poor health: Modeling the relationship between road traffic noise and cardiovascular problems. *Sci. Total Environ.* **2010**, *408*, 4935–4942.
72. Jakovljević, B.; Belojević, G.; Paunović, K.; Stojanov, V. Road traffic noise and sleep disturbances in an urban population: Cross-sectional study. *Croat. Med. J.* **2006**, *47*, 125–133.
73. Ohrstrom, E.; Skanberg, A.; Svensson, H.; Gidlof-Gunnarsson, A. Effects of road traffic noise and the benefit of access to quietness. *J. Sound Vib.* **2006**, *295*, 40–59.
74. Stošić, L.; Belojević, G.; Milutinović, S. Effects of traffic noise on sleep in an urban population. *Arh. Hig. Rada Toksikol.* **2009**, *60*, 335–342.
75. Blockmans, D.; Vandeputte, A.; Masschelein, R. Enquiry about health problems in a population living near an international airport. *Tijdschr. Geneeskd.* **2002**, *58*, 1398–1406.
76. Boes, S.; Nuesch, S.; Stillman, S. Aircraft noise, health, and residential sorting: Evidence from two quasi-experiments. *Health Econ.* **2013**, *22*, 1037–1051.
77. De Carvalho, E.B., Jr.; Garavelli, S.L.; Maroja, A.M. Analysis of the effects of aircraft noise in residential areas surrounding the Brasilia International Airport. In Proceedings of the 18th International Congress on Sound and Vibration, Rio de Janeiro, Brazil, 10–14 July 2011; pp. 413–420.
78. Magari, S.R.; Smith, C.E.; Schiff, M.; Rohr, A.C. Evaluation of community response to wind turbine-related noise in Western New York State. *Noise Health* **2014**, *16*, 228–239.
79. Zannin, P.H.T.; Bunn, F. Noise annoyance through railway traffic—A case study. *J. Environ. Health Sci. Eng.* **2014**, *12*, 14.
80. De Kluizenaar, Y.; Janssen, S.A.; van Lenthe, F.J.; Miedema, H.M.; Mackenbach, J.P. Long-term road traffic noise exposure is associated with an increase in morning tiredness. *J. Acoust. Soc. Am.* **2009**, *126*, 626–633.

81. Amundsen, A.H.; Klaeboe, R.; Aasvang, G.M. The Norwegian Facade Insulation Study: The efficacy of facade insulation in reducing noise annoyance due to road traffic. *J. Acoust. Soc. Am.* **2011**, *129*, 1381–1389.
82. Amundsen, A.H.; Klaeboe, R.; Aasvang, G.M. Long-term effects of noise reduction measures on noise annoyance and sleep disturbance: The Norwegian facade insulation study. *J. Acoust. Soc. Am.* **2013**, *133*, 3921–3928.
83. Anh, P.T.H.; Yen, P.T.H.; Cuong, T.D.; Dang, P.N.; Nai, L.V.; Nishimura, T.; Sato, T.; Hashimoto, Y.; Yano, T. Characteristics of road traffic noise in hanoi and community response to noise. In Proceedings of the 12th International Congress on Sound and Vibration, Lisbon, Portugal, 11–14 July 2005; pp. 2013–2020.
84. Banerjee, D.; Chakraborty, S.K.; Bhattacharyya, S.; Gangopadhyay, A. Attitudinal response towards road traffic noise in the town of Asansol, India. *Environ. Monit. Assess.* **2009**, *151*, 37–44.
85. Fidell, S.; Silvati, L.; Haboly, E. Social survey of community response to a step change in aircraft noise exposure. *J. Acoust. Soc. Am.* **2002**, *111*, 200–209.
86. Floud, S.; Vigna-Taglianti, F.; Hansell, A.; Blangiardo, M.; Houthuijs, D.; Breugelmans, O.; Cadum, E.; Babisch, W.; Selander, J.; Pershagen, G.; et al. Medication use in relation to noise from aircraft and road traffic in six European countries: Results of the HYENA study. *Occup. Environ. Med.* **2011**, *68*, 518–524.
87. Fooladi, M.M. Involuntary and persistent environmental noise influences health and hearing in Beirut, Lebanon. *J. Environ. Public Health* **2012**, *2012*, 235618.
88. Goswami, S. Road traffic noise: A case study of Balasore town, Orissa, India. *Int. J. Environ. Res.* **2009**, *3*, 309–316.
89. Goswami, S.; Nayak, S.K.; Pradhan, A.C.; Dey, S.K. A study on traffic noise of two campuses of University, Balasore, India. *J. Environ. Biol.* **2011**, *32*, 105–109.
90. Kim, S.J.; Chai, S.K.; Lee, K.W.; Park, J.B.; Min, K.B.; Kil, H.G.; Lee, C.; Lee, K.J. Exposure-response relationship between aircraft noise and sleep quality: A community-based cross-sectional study. *Osong Public Health Res. Perspect.* **2014**, *5*, 108–114.
91. Nilsson, M.E.; Berglund, B. Noise annoyance and activity disturbance before and after the erection of a roadside noise barrier. *J. Acoust. Soc. Am.* **2006**, *119*, 2178–2188.
92. Yokoshima, S.; Morihara, T.; Sano, Y.; Ota, A.; Tamura, A. Community response to Shinkansen Railway vibration. In Proceedings of the 40th International Congress and Exposition on Noise Control Engineering, InterNoise, Osaka, Japan, 4–7 September 2011; pp. 929–936.
93. Mohammadi, G. An investigation of community response to urban traffic noise. *Iran. J. Environ. Health Sci. Eng.* **2009**, *6*, 137–142.
94. Öhrström, E.; Skånberg, A. Longitudinal surveys on effects of road traffic noise: Substudy on sleep assessed by wrist actigraphs and sleep logs. *J. Sound Vib.* **2004**, *272*, 1097–1109.
95. Kishikawa, H.; Matsui, T.; Uchiyama, I.; Miyakawa, M.; Hiramatsu, K.; Stansfeld, S.A. Noise sensitivity and subjective health: Questionnaire study conducted along trunk roads in Kusatsu, Japan. *Noise Health* **2009**, *11*, 111–117.
96. Kawada, T.; Yosiaki, S.; Yasuo, K.; Suzuki, S. Population study on the prevalence of insomnia and insomnia-related factors among Japanese women. *Sleep Med.* **2003**, *4*, 563–567.
97. Kiani Sadr, M.; Nassiri, P.; Sekhavatjo, M.; Abbaspour, M. Noise pollution assessment in khoramabad to presenting executive strategies to control or reduce it. *J. Environ. Stud.* **2009**, *35*, 83–96.
98. Keefe, M.R. Comparison of neonatal nighttime sleep-wake patterns in nursery versus rooming-in environments. *Nurs. Res.* **1987**, *36*, 140–144.
99. Smith, A.; Nutt, D.; Wilson, S.; Rich, N. *Noise and Insomnia: A Study of Community Noise Exposure, Sleep Disturbance, Noise Sensitivity*; Institute for Environment and Health: Leicester, UK, 2002.
100. Salavitabar, A.; Haidet, K.K.; Adkins, C.S.; Susman, E.J.; Palmer, C.; Storm, H. Preterm infants' sympathetic arousal and associated behavioral responses to sound stimuli in the neonatal intensive care unit. *Adv. Neonatal Care* **2010**, *10*, 158–166.
101. Aasvang, G.M.; Engdahl, B.; Rothschild, K. Annoyance and self-reported sleep disturbances due to structurally radiated noise from railway tunnels. *Appl. Acoust.* **2007**, *68*, 970–981.
102. Agarwal, S.; Swami, B.L. Road traffic noise, annoyance and community health survey—A case study for an Indian city. *Noise Health* **2011**, *13*, 272–276.

103. Banerjee, D. Road traffic noise and self-reported sleep disturbance: Results from a cross-sectional study in western India. *Noise Vib. Worldw.* **2013**, *44*, 10–17.
104. Bocquier, A.; Cortaredona, S.; Boutin, C.; David, A.; Bigot, A.; Sciortino, V.; Nauleau, S.; Gaudart, J.; Giorgi, R.; Verger, P. Is exposure to night-time traffic noise a risk factor for purchase of anxiolytic-hypnotic medication? A cohort study. *Eur. J. Public Health* **2014**, *24*, 298–303.
105. Fidell, S.; Pearsons, K.; Tabachnick, B.G.; Howe, R. Effects on sleep disturbance of changes in aircraft noise near three airports. *J. Acoust. Soc. Am.* **2000**, *107*, 2535–2547.
106. Franssen, E.A.M.; van Wiechen, C.M.A.G.; Nagelkerke, N.J.D.; Lebret, E. Aircraft noise around a large international airport and its impact on general health and medication use. *Occup. Environ. Med.* **2004**, *61*, 405–413.
107. Han, J.W.; Ji, H.Y.; Son, J.H.; Chang, S.I.; Kim, J.H. Health effect study of metropolitan railway noise in Seoul, Korea. In Proceedings of the 39th International Congress and Exposition on Noise Control Engineering, InterNoise, Lisbon, Portugal, 13–16 June 2010; pp. 3055–3060.
108. Koushki, P.A.; Al-Rukaibi, F. Airport noise and its impact on exposed urban population in Kuwait. *Kuwait J. Sci. Eng.* **2009**, *36*, 53–78.
109. Kristiansen, J.; Persson, R.; Bjork, J.; Albin, M.; Jakobsson, K.; Ostergren, P.-O.; Ardo, J. Work stress, worries, and pain interact synergistically with modelled traffic noise on cross-sectional associations with self-reported sleep problems. *Int. Arch. Occup. Environ. Health* **2011**, *84*, 211–224.
110. Nathanail, C. Urban environmental noise in Greece: A social survey. In Proceedings of the International Congress and Exposition on Noise Control Engineering, InterNoise, Rio de Janeiro, Brazil, 7–10 August 2005; pp. 1402–1411.
111. Niemann, H.; Bonnefoy, X.; Braubach, M.; Hecht, K.; Maschke, C.; Rodrigues, C.; Robbel, N. Noise-induced annoyance and morbidity results from the pan-European LARES study. *Noise Health* **2006**, *8*, 63–79.
112. Sobotová, L.; Jurkovičová, J.; Voleková, J.; Aghová, L. Community noise annoyance risk in two surveys. *Int. J. Occup. Med. Environ. Health* **2001**, *14*, 197–200.
113. Sobotová, L.; Aghová, L.; Jurkovičová, J.; Voleková, J. Evaluation of the risk of exposure to noise in a group of university students. *Hygiena* **2000**, *45*, 109–118.
114. Van Renterghem, T.; Botteldooren, D. Focused Study on the Quiet Side Effect in Dwellings Highly Exposed to Road Traffic Noise. *Int. J. Environ. Res. Public Health* **2012**, *9*, 4292–4310.
115. Hu, F.B.; Goldberg, J.; Hedeker, D.; Flay, B.R.; Pentz, M.A. Comparison of population-averaged and subject-specific approaches for analyzing repeated binary outcomes. *Am. J. Epidemiol.* **1998**, *147*, 694–703.
